# Comprehensive Lineage Tracing Maps the Landscape of Cell Fate Decisions in Mouse Embryogenesis

**DOI:** 10.64898/2026.05.07.722278

**Published:** 2026-07-17

**Authors:** William N. Colgan, Luke W. Koblan, JoAnne Villagrana, Tien-Chi Jason Hou, Minming Wang, Gokul Gowri, Whitney Chandler, Leonardo A. Sepúlveda, Didar Ciftci, Karina Smolyar, Alicia Young, Lars Wittler, Styliani Markoulaki, Kyle Loh, Xiaowei Zhuang, Nir Yosef, Zachary D. Smith, Jonathan S. Weissman

**Affiliations:** 1 Whitehead Institute for Biomedical Research; Cambridge MA, 02142, USA; 2 Howard Hughes Medical Institute; Chevy Chase MD, 20815, USA; 3 David H. Koch Center for Integrative Cancer Research, Massachusetts Institute of Technology, Cambridge, MA 02139, USA; 4 Department of Biology, Massachusetts Institute of Technology, Cambridge MA 02139 USA.; 5 Department of Genetics, Yale Stem Cell Center, Yale School of Medicine; New Haven CT 06519, USA; 6 Laboratory for Information and Decision Systems, Massachusetts Institute of Technology, Cambridge, MA, 02139, USA; 7 Schwarzman College of Computing, Massachusetts Institute of Technology, Cambridge, MA 02139, United States; 8 Department of Chemistry and Chemical Biology, Harvard University; Cambridge MA, 02138, USA; 9 Department of Physics, Harvard University; Cambridge MA, 02138, USA; 10 Max Planck Institute of Molecular Genetics, Max Planck Society, Berlin DE, 14195; 11 Current address: Targeted Mutagenesis Laboratory, Feinberg School of Medicine, Northwestern University; Chicago IL, 60611, USA; 12 Department of Developmental Biology and Institute for Stem Cell Biology and Regenerative Medicine, Stanford University, Stanford CA, 94305, USA.; 13 Department of Systems Immunology, Weizmann Institute of Science; Rehovot, 7610001, Israel

## Abstract

A comprehensive cell fate map of mammalian embryogenesis has remained out of reach due to the scale, cellular diversity, and non-deterministic nature of development *in utero*. Here, we use PEtracer to continuously install heritable genetic marks as cells divide, reconstructing lineage trees that resolve ~75% of cell divisions across >1.5 million cells from 16 mouse embryos collected at half-day intervals from E7.5-E10.0. We pair these trees with deep transcriptional profiling to chart the landscape of cell fate decisions during gastrulation and early organogenesis. Using these data, we quantify cell fate biases, restriction timing, progenitor pool sizes, and lineage relationships across the embryo, revealing strikingly reproducible lineage architecture across replicate embryos despite the regulative flexibility of mammalian development. We further show how lineage, spatial position, and signaling jointly determine fate outcomes and timing, with their relative influence varying by tissue. This dataset provides a quantitative framework for understanding cell fate specification and a lineage-resolved reference for generating and contextualizing developmental hypotheses at organismal scale.

## Introduction

Mammalian development is a non-deterministic process that reliably transforms a single fertilized cell into hundreds of distinct cell types, which are collectively organized into a conserved body plan^1^. A comprehensive cell fate map–the complete record of every progenitor-progeny relationship through which cellular diversity and morphogenetic complexity arise–would transform our understanding of this process^2^. Such a record would reveal how robust developmental outcomes emerge from probabilistic, context-dependent differentiation trajectories, define how cellular form and function are specified across successive divisions, and provide a quantitative blueprint for tissue assembly and patterning^3^.

The complete lineage map of *C. elegans*, reconstructed through direct observation of every cell division from zygote to adult, represents a landmark achievement in developmental biology^4^. Similar approaches have been extended to zebrafish and other transparent organisms, where live cell tracking has been used to reconstruct the reproducible cell movements and lineage relationships underlying early embryogenesis^5–8^. However, in mammals, obligate *in utero* development–combined with the scale, regulative complexity, and diversity of cell types and states–has limited the application of comparable approaches^8,9^. As such, quantitative descriptions of how single-cell behaviors give rise to the reproducible outcomes of mammalian development have remained challenging to fully realize.

Decades of technical innovation have provided increasingly resolved spatial and temporal views of cell fate relationships for key steps throughout mammalian embryogenesis. Classical fate-mapping studies used vital dyes to label cells in early mouse embryos and track their progeny throughout development^10,11^. Subsequent advances including recombinase-based genetic labeling and static DNA barcoding strategies expanded the repertoire of heritable markers and enabled cell type-specific control over their activation^2,12–17^ providing insights into how progenitor populations contribute to tissue patterning and general descriptions of lineage potentials. In parallel, advances in scalable single-cell profiling revealed the rich diversity of cell types and states across embryogenesis, permitting an increasingly detailed molecular view of the developmental landscape^18–26^. Together, these approaches establish a powerful framework for understanding the cellular, molecular, and spatial organization of mammalian embryogenesis, but resolving the full set of fate restriction events underlying mouse embryogenesis has remained out of reach.

Reconstructing complete cell fate restriction trajectories in mouse development requires continuous marking of dividing cells, sufficient recording capacity to resolve millions of cell divisions, and the ability to jointly read out lineage information and cell state at single-cell resolution. Genome editing-based lineage tracing systems offer a framework for achieving this by installing heritable marks as cells divide^27–40^; however, combining the necessary recording density and continuous cell marking across mammalian development has remained challenging. Recently described prime editing-based lineage recording systems meet many of these requirements^41,42^. In particular, we recently developed PEtracer, which continuously installs pre-defined, heritable lineage marks (LMs) at genomically integrated lineage tracing cassettes (LTCs) without relying on the formation of cytotoxic double-stranded DNA breaks (DSBs)^42–44^. PEtracer editing rates are fast enough to resolve individual cell divisions in rapidly dividing embryonic cells and can be tuned to capture lineage dynamics over longer timescales. Further, this system is compatible with both dissociated single-cell sequencing and imaging-based spatial readouts (*e.g.*, Multiplexed, error-robust fluorescence in situ hybridization [MERFISH])^45^, enabling scalable, joint measurement of lineage and cell state across modalities.

Here, we use PEtracer to chart cell state and lineage dynamics in early mouse embryogenesis. We generated high-grade chimeric embryos (up to 99% chimerism) by introducing engineered mouse embryonic stem cells (mESCs) carrying the full PEtracer system, including more than 100 editable sites, into host morulae. We then performed comprehensive single-cell RNA-seq (scRNA-seq) profiling of entire embryos at half-day intervals from E7.5 to E10.0. This allowed us to determine each cell’s current state, as measured by mRNA levels, together with its lineage history. In total, we recovered >1.7 million cells across 16 embryos, with triplicate sampling at all but the final timepoint. By sampling approximately half of all cells and resolving ~75% of divisions along recovered branches, we generate high-confidence trees that resolve the landscape of cellular fate decisions across the developing embryo. This sampling approach also allows us to capture late cell divisions and fate commitment events that are systematically missed in subsampled datasets.

These data reveal how cell fate unfolds across mouse gastrulation and early organogenesis. We resolve progenitor populations, fate restriction timing, and clonal outputs. These features are strikingly reproducible across embryos, despite the regulative flexibility of mammalian development. Detailed analyses across specific cell types and tissues–including notochord specification, heart field separation, neural crest fate restriction, endothelial origins, and axial progenitors–illustrate how cell fates are jointly instructed by lineage, spatial position, and local signaling. Together, these data serve as a foundational resource for interpreting developmental experiments against a shared lineage-resolved reference, as well as generating new hypotheses about how developmental programs unfold in space and time. As such, this study represents an important step toward a comprehensive fate map of mammalian development.

## Results

### Enabling PEtracer Studies of Mouse Development

**Fig. 1.**
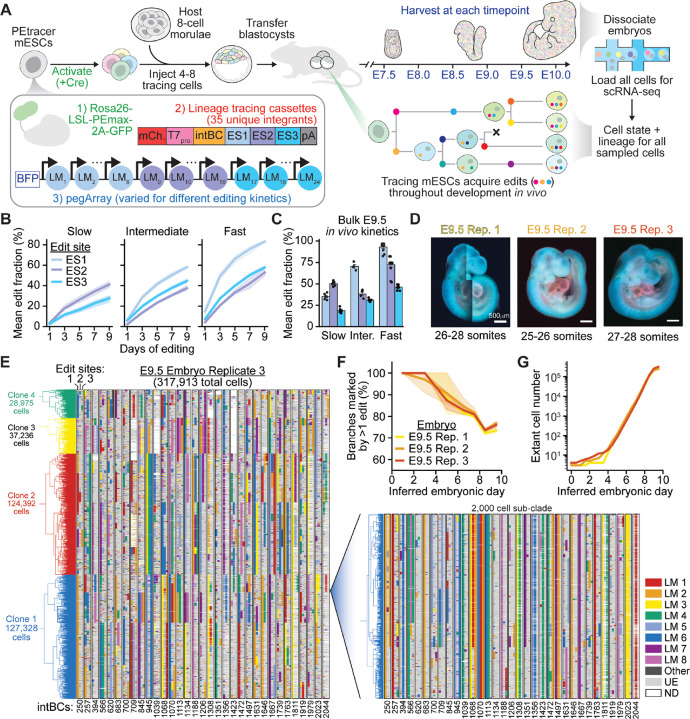
Cell fate mapping of mouse development at embryonic scale with PEtracer. (**A**) Mouse embryonic stem cells (mESCs) were engineered with the three core PEtracer components: a Rosa26 knock-in of Cre-activatable PEmax-T2A-GFP, genomically integrated lineage tracing cassettes (LTCs) each containing a unique integration barcode (intBC) and three distinct edit sites (ESs), and pegRNA arrays (pegArrays) encoding 24 lineage marks (LMs; eight per ES). Following Cre-mediated PEmax activation, 4–8 donor mESCs were introduced into host 8-cell morulae and transferred into pseudopregnant recipients at the blastocyst stage. As cells divide, LMs accumulate at LTCs and can be read out by dissociative scRNA-seq, enabling phylogenetic reconstruction of developmental lineage relationships at embryonic scale. (**B**) Editing kinetics assayed by bulk DNA sequencing for slow, intermediate, and fast pegArray lines at edit sites 1–3 in cultured mESCs and (**C**) in chimeric E9.5 embryos. Bars represent the mean of at least four biological replicates ± standard error of the mean. (**D**) Images of E9.5-stage embryos prior to dissociation and scRNA-seq. Colors represent an overlay of the three core PEtracer components (PEmax-T2A-GFP, mCherry:LTC, and BFP-linked pegArray). Rep. = Replicate. (**E**) Cellular lineage tree and character matrix for 317,931 cells across four mESC clones comprising E9.5 embryo replicate 3, with a zoom-in on a 2,000-cell sub-clade from clone 1. intBCs are listed below the character matrix which is colored by LM identity for each ES; UE = unedited, ND = not detected. (**F**) Mean fraction of branches marked by at least one edit across three E9.5 embryo trees as a function of inferred embryonic day with ribbons indicating 95% confidence interval. (**G**) Estimated number of extant cells over inferred embryonic day based on branch length estimates for E9.5 replicate embryos.

To enable joint profiling of an embryonic cell’s transcriptional state and lineage history, we engineered the PEtracer components into mouse embryonic stem cells (mESCs) (Fig. 1A). This system comprises three core components: 1) the PEmax editor, which installs LMs at target sites; 2) multiple genomically integrated LTCs, each containing a unique integration barcode (intBC) and three editable sites (ESs); and 3) pegRNA arrays (pegArrays), comprising 24 pegRNAs that install distinct 5-nucleotide lineage marks (LMs) (8 per ES). PEtracer uses prime editing to continuously install these pre-defined LMs at LTCs throughout development. Individual cells accumulate unique combinations of LMs over time, encoding their division history in a form that can be read out by scRNA-seq of polyadenylated LTC mRNAs or imaging-based spatial profiling (e.g., MERFISH).

We first generated a karyotypically normal mESC line harboring a homozygous *Rosa26* knock-in of Cre-activatable PEmax-T2A-GFP, and subsequently introduced 35 unique LTCs throughout the genome, providing 105 editable sites for lineage recording (figs. S1A,B; Methods). *In silico* simulations indicate that, with appropriately tuned tracing kinetics, >100 editable sites are sufficient to generate high-resolution phylogenies for cell populations exceeding 10^9^ cells–a scale encompassing the entirety of mouse development^42^. To achieve an optimal rate of LM accumulation over time, we generated a series of mESC lines with pegArrays tuned to produce distinct LM installation rates using engineered pegRNA protospacer mismatches. *In vitro* characterization confirmed the expected differences in lineage tracing kinetics between slow, intermediate, and fast kinetics lines and showed well-balanced LM installation frequencies across edit sites (Fig. 1B, fig. S1C).

To apply PEtracer to mouse embryogenesis *in vivo*, we generated chimeric embryos by introducing multiple Cre-recombinase-activated mESCs into host embryos at the 4-to-8 cell stage (~E2-E2.5) (Fig. 1A), which promotes robust contribution of the introduced mESCs to the inner cell mass^46^. Because all donor cells derive from a common engineered starting population, this approach enables highly reproducible lineage tracing across replicates. Importantly, each of the multiple founding donor cells provides independent lineage trajectories that can be compared, while host-derived wild-type, euploid cells serve as an internal control for developmental competence and lineage contribution. We generated chimeric embryos from slow, intermediate, and fast pegArray lines and profiled them at E9.5. All three lines produced embryos with normal morphologies and high (mean 69%, range 31–94%) estimated chimerism rates (figs. S1D,E). Importantly, *in vivo* lineage tracing kinetics were remarkably consistent with *in vitro* rates (Fig. 1C, figs. S1F,G).

Based on these data, we selected the intermediate kinetics line as best suited for high-resolution lineage reconstruction across multiple early- and mid-gestation timepoints, as it provided substantial information content without saturation during this interval. To validate this selection, we performed targeted profiling of two intermediate kinetics E9.5 embryos, confirming expected contributions of tracing cells to inner cell mass (ICM)-derived cell types and minimal bias between mESC clones comprising these embryos (figs. S1E, S1H–J). We further validated our system by injecting activated PEtracer mESCs into tetraploid host morulae, which confirmed no obvious adverse effects of lineage tracing on embryogenesis both during our collection window and to later timepoints (figs. S1K,L).

These results motivated our efforts to perform near-complete cellular sampling at embryonic scale. We profiled three chimeric E9.5 embryos in their entirety by scRNA-seq and recovered between 342,421 and 382,281 high-quality cells per embryo (an estimated 55.9% of total cells, on average) (Figs. 1D,E; Table S1). We reconstructed lineage trees using a scalable heuristic neighbor-joining approach (Methods), revealing robust lineage recording, with an average of 75% of branches marked by at least one edit across early development (Figs. 1E,F). By inferring division timing from the number of edits accumulated along each branch–where more edits indicate longer intervals between divisions–we obtained highly reproducible estimates of developmental time (Fig. 1G). These estimates revealed division rates consistent with known patterns of embryonic growth across all three embryos, recapitulating the expected transition from slower early divisions during ICM specification and epiblast maturation to rapid growth as gastrulation and organogenesis unfold (Fig. 1G)^47,48^. The resolution and completeness of these trees motivated comprehensive profiling across the full window of gastrulation and early organogenesis.

### Comprehensive Cell State and Lineage Profiling of Early Mouse Embryogenesis

To generate a time-resolved atlas of cell state and lineage dynamics across early mouse development, we sampled embryos from E7.5 to E10.0 at half-day intervals by dissociating the whole embryo and performing scRNA-seq. This window captures the later stages of gastrulation, as well as axis elongation and early organogenesis, with embryonic cell number increasing nearly three orders of magnitude from a few thousand to approximately one million cells^49,47,50,25^. We collected triplicate samples for the E7.5-E9.5 timepoints and profiled all dissociated cells from each embryo, with an average representation of 44%. This sampling depth was necessary to resolve late cell fate restriction events that would otherwise be lost in subsampled lineage trees, enabling reconstruction of a comprehensive atlas spanning the full cellular diversity of early mouse embryogenesis.

**Fig. 2.**
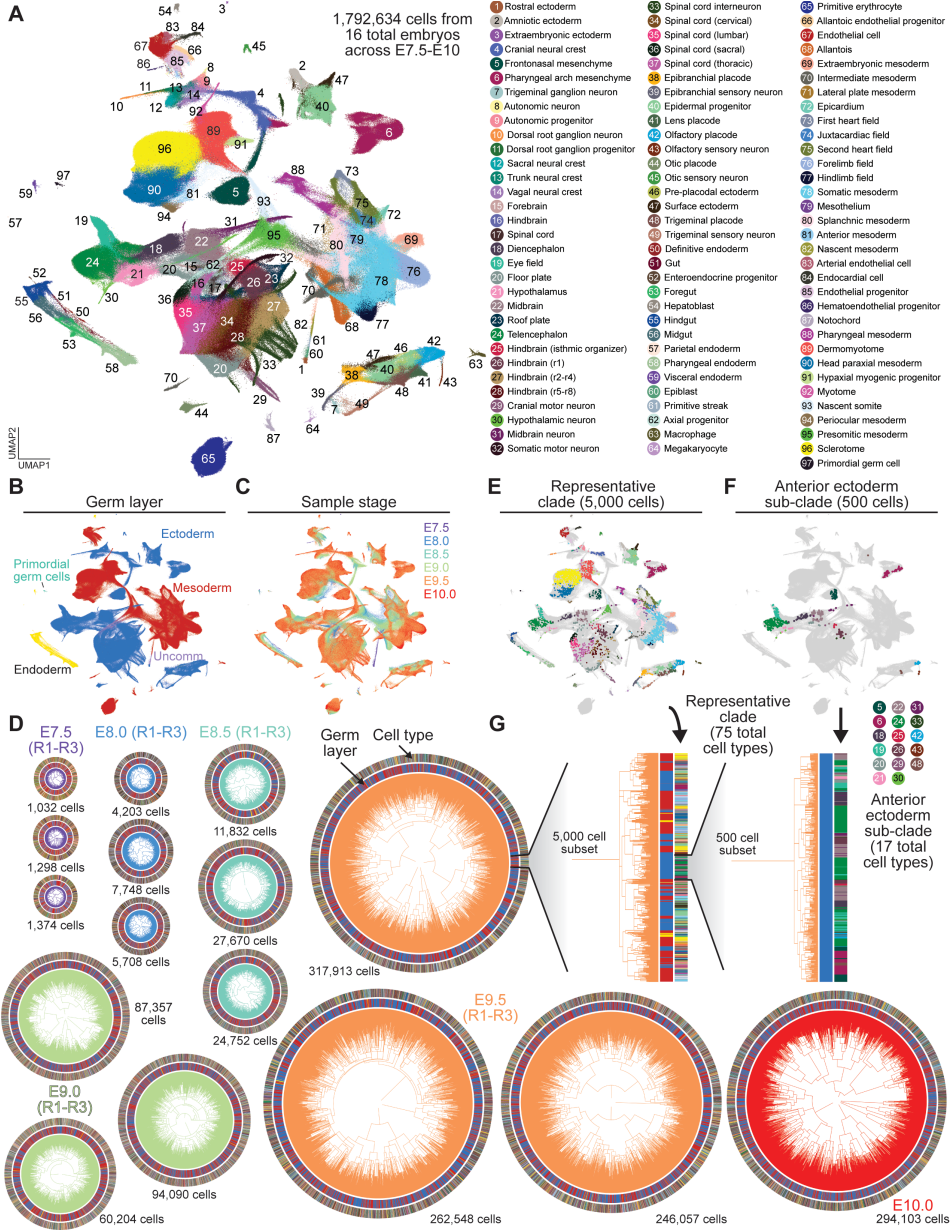
Whole-embryo joint cell state and lineage atlas of early mouse embryogenesis. UMAP embedding of 1,792,634 cells from 16 embryos spanning E7.5 to E10.0, colored by: (**A**) cell type, resolving 97 distinct cell types, see figs. S4 and S5 for 140 sub-clusters; (**B**) broad germ layer assignment: ectoderm, mesoderm, endoderm, primordial germ cells, and uncommitted; and (**C**) sampled embryonic stage. (**D**) Lineage trees for 16 comprehensively sampled embryos, colored by sample stage (1,447,907 cells). Outer rings indicate cell type and germ layer assignment as in (A) and (B); cell number is indicated for each tree. R=Replicate. (**E**) UMAP embedding colored by cell type for a representative 5,000-cell clade from the E9.5 replicate 3 tree and (**F**) a 500-cell anterior ectoderm restricted sub-clade. (**G**) Zoom-ins of the cellular lineage tree corresponding to the 5,000- and 500-cell clades shown in (E) and (F).

Across 16 embryos, we recovered 1,792,634 high-quality cells at a mean depth of 16,847 UMIs per cell (Fig. 2A; Table S1). Transcriptional state distributions were highly consistent across embryos collected at the same timepoint, with minimal batch effects (figs. S2A, B). Integration with existing atlases confirmed expected cell type distributions and enabled precise transcriptional staging of each embryo alongside our own morphological assessment (figs. S2C–I, fig. S3)^25^. Together, these approaches revealed natural variation of up to a few hours in developmental progression among embryos collected at the same sampled timepoint, consistent with expected variation in normal development^25,51^.

From these data, we resolved 97 transcriptionally-distinct cell types and 140 sub-types spanning embryonic and extraembryonic tissues (Figs. 2A–C, fig. S4, fig. S5; Table S2). The breadth of sampling and sequencing depth enabled detection of rare cell populations such as primordial germ cells, representing <0.02% of cells at E9.5, and the resolution of 24 distinct, post-mitotic neuron subtypes (figs. S5A,D,E, fig. S6A). Across timepoints, chimerism rates–the fraction of tracing donor cells per embryo–ranged from 52–99%, with near total mESC contributions at later timepoints (fig. S6B; Table S1). Within each chimeric embryo, host and donor cells reproducibly populate near-identical transcriptional spaces across replicates and timepoints, indicating that PEtracer does not perturb normal cell fate specification (fig. S6C). As expected, donor PEtracer mESCs showed minimal contribution to extraembryonic endoderm and extraembryonic ectoderm (figs. S6C–E)^52,53^.

To link the transcriptional identities of profiled cells to their division histories, we reconstructed cellular lineage trees for each sampled embryo (Fig. 2D). The average LM detection rate was 94%, with the majority of missing data being driven by infrequent, heritable epigenetic silencing of individual barcodes^54–57^ (Fig. 1E, fig. S6F). In total, we reconstructed trees for 1,447,907 donor cells, ranging from 1,032 to 317,931 cells per embryo (Table S1). Across reconstructed trees, 98% of cells had a unique edit profile and approximately 75% of cell divisions were marked by at least one edit, enabling high-confidence tree reconstructions (figs. S6G,H). For all stages, the inferred number of extant cells over time closely tracked the actual number of cells observed in the corresponding dissociated embryos, further validating the accuracy of branch length estimates (fig. S6I). The plateaus observed at the end of each growth curve are consistent with cell loss due to experimental constraints (*e.g.*, dissociation and single-cell capture). Notably, PEmax expression and LM installation rates were consistent across cell types, confirming unbiased lineage recording and enabling faithful resolution of developmental dynamics throughout the embryo (fig. S6J). These phylogenies capture both pre- and post-gastrulation dynamics, with broad germ layer structure evident at whole-embryo scale and progressively finer sub-clades resolving to increasingly lineage-restricted groups (Figs. 2E–G).

### Quantifying germ layer cell fate restriction dynamics

By pairing high-resolution developmental lineages with transcriptional state information across >1.4 million cells, we sought to dissect the dynamics of cell fate restriction during mouse embryogenesis. Here, we define cell fate operationally as the observed distribution of descendant cell types, rather than as an inference of developmental potential^23,58,3^. Examining ancestral nodes within our reconstructed trees, we asked when fates became restricted, whether biases emerged prior to full specification, and how the number and output of fate-restricted clades change over time. We used germ layer specification as an initial framework for these analyses.

**Fig. 3.**
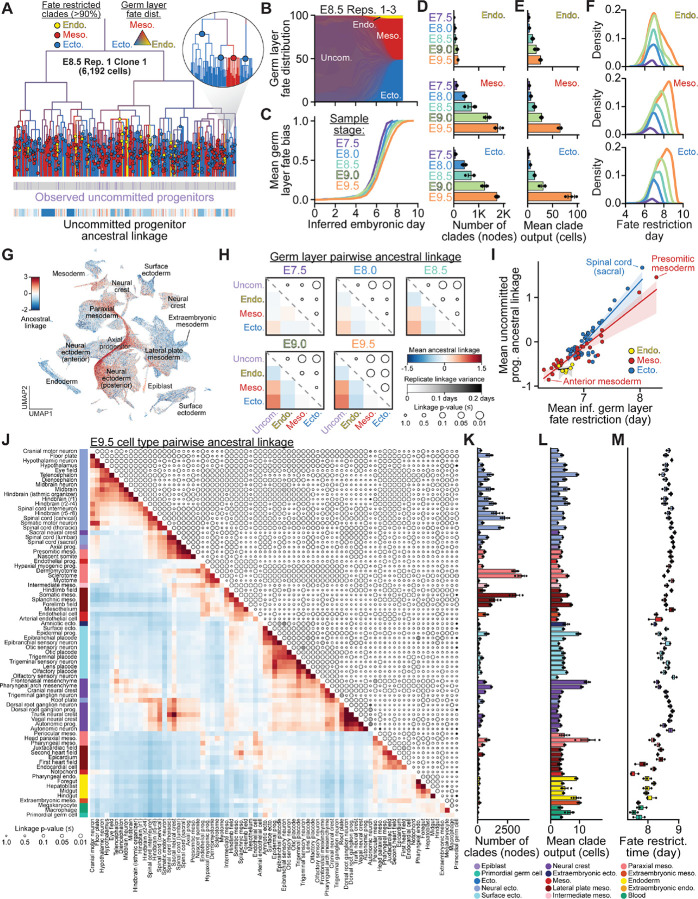
Cell fate bias, pool size, and fate restriction timing by germ layer and cell type. (**A**) Representative cellular lineage tree for E8.5 replicate 1 clone 1 (E8.5-R1-C1), comprising 6,192 cells. Fate-restricted nodes (>90% representation from a single germ layer) are colored on the tree: yellow = endoderm (Endo.), red = mesoderm (Meso.), blue = ectoderm (Ecto.). Branches are colored by the fate distribution of descendant cells; triangle inset shows the edge color scheme. Inset sub-tree highlights these metrics at higher resolution. Color bars below the tree indicate uncommitted progenitors recovered at the time of sampling and their ancestral linkage to all other sampled cells in the tree. (**B**) Relative abundance of germ layer fate distributions over time across triplicate E8.5 embryos. (**C**) Mean germ layer fate bias for triplicate-sampled embryos from E7.5 to E9.5. Bias was calculated as one minus the normalized entropy of the descendant fate distributions at each node, relative to the entropy at the root of the same tree. (**D**) Number of fate-restricted clades for each germ layer by sampled timepoint (E7.5-E9.5). (**E**) Mean number of descendant cells per fate-restricted clade for each germ layer. For (D) and (E), bars represent the mean of three biological replicate embryos ± standard error of the mean. (**F**) Distribution of inferred germ layer fate restriction timing across sampled developmental stages. (**G**) UMAP of all sampled cells colored by ancestral linkage to uncommitted progenitors. Ancestral linkage was calculated as the distance to the most recent common ancestor (MRCA) of a given cell type minus the permuted background expectation. (**H**) Mean pairwise ancestral linkage between cells assigned to each germ layer or uncommitted progenitors by sampled timepoint. Below the diagonal, heatmap is colored by ancestral linkage values; above the diagonal, circles are colored by variance across three replicate embryos and sized by p-value (calculated by permutation test; larger circles = smaller p-values). (**I**) Mean ancestral linkage of each cell type to uncommitted progenitors at E9.5 versus mean inferred (inf.) germ layer commitment timing. Points are colored by germ layer assignment. Cell types with notably low or high ancestral linkages are labeled. (**J**) Mean pairwise ancestral linkage across all cell types, plotted as in (H). Cell types are annotated on both axes; color blocks along the axes indicate developmental domain (legend, bottom right). (**K**) Count, (**L**) mean number of descendants, and (**M**) commitment timing of cell type-restricted clades, colored by developmental domain.

For each internal node in our reconstructed trees, we calculated a fate distribution based on the germ layer composition of its descendant cells (Fig. 3A). When plotted against inferred developmental time, ancestral fate distributions were initially consistent with pluripotency, producing all three germ layers, before becoming increasingly biased toward specific germ layers during the window associated with gastrulation (~E6.5 to ~E7.5) (Fig. 3B). To quantify fate restriction dynamics, we defined a fate bias index based on the normalized entropy of each node’s fate distribution across states (Methods), which revealed highly reproducible specification trajectories across timepoints and embryos (Figs. 3B,C). Significant germ layer fate bias first appeared at ~E4.0–around the time of implantation but well before the onset of gastrulation–suggesting that factors such as spatial organization and signaling cues in the epiblast contribute to early fate skewing prior to differentiation (Figs. 3B,C, fig. S7A).

We next defined ancestral nodes for which >90% of descendants belonged to a single fate–here, any one germ layer–as “fate-restricted” nodes (Fig. 3A). This allowed us to quantify the number of fate-restricted clades contributing to each germ layer, the size of their descendant cell populations, and the timing of fate restriction. Across sampled timepoints, we observed consistent increases in both the number of clades and the number of descendant cells within each clade, particularly for ectoderm and mesoderm (Figs. 3D, E, fig. S7B). Notably, the number of germ layer fate-restricted nodes at a given depth in the tree was consistent across independently-sampled triplicate embryos from E7.5 to E9.5, as were the general rates of each germ layer’s emergence. On average, endodermal fate restriction reproducibly occurred earliest, around E7.0 (Fig. 3F)^15^. In contrast, ectodermal and mesodermal fate restriction often occurred later and over a broader interval, driven by continued emergence of clades contributing to a subset of cell types (Fig. 3F, figs. S7B,C).

To understand why ectodermal and mesodermal fate restriction span a broader developmental window than endoderm, we asked whether uncommitted progenitors–defined transcriptionally as epiblast cells at E7.5 and axial progenitors at later timepoints–maintain preferential lineage relationships with specific germ layers over time. To quantify this, we defined an ancestral linkage metric based on the lineage distance from any source cell to the nearest member of a specified target population, normalized against a permuted background expectation (Methods)^33,58^. Positive values indicate more recent than expected shared ancestry, whereas negative values indicate earlier fate separation. We computed the ancestral linkage of all cells to uncommitted progenitors and to each germ layer (Fig. 3G, fig. S7D). Across timepoints, posterior ectodermal and mesodermal populations showed the strongest linkage to uncommitted progenitors.

To capture broad trends in lineage relationships across germ layers and uncommitted progenitors, we computed mean pairwise ancestral linkage (Methods) between these populations at each timepoint (Fig. 3H). At E7.5, the only significant association was between ectoderm and the uncommitted progenitors. However, as development progressed, both ectoderm and mesoderm became increasingly linked to the uncommitted progenitors, while endoderm diverged from the other populations. These relationships were highly reproducible across replicates (Fig. 3H). For both ectodermal and mesodermal derivatives, mean fate restriction time was strongly correlated with ancestral linkage to uncommitted progenitors (Fig. 3I). This correlation reflected a clear anterior-posterior (A-P) organization: the most posterior cell types, such as presomitic mesoderm and sacral spinal cord, maintained the strongest linkage to uncommitted progenitors, while anterior populations diverged earliest (Fig. 3I).

### Lineage architecture of cell type diversification

Having established the dynamics of germ layer specification, we next sought to resolve how individual cell types emerge from these broad developmental compartments. To do this, we computed mean pairwise ancestral linkage across 77 cell types at E9.5, generating a matrix that captures patterns of shared ancestry. Clustering this matrix revealed blocks of closely related cell types, as well as additional structure within and between blocks that reflects lineage history and spatial patterning across the embryo (Fig.3J). These relationships were remarkably consistent (less than six-hour variance) across triplicate E9.5 embryos. We also performed linkage analysis at the cell subtype level to map patterns of shared ancestry within clusters and across sampled timepoints (figs. S4, S5).

Neural ectodermal cell types formed a large cluster structured primarily by position along the anterior-posterior (A-P) axis (Fig. 3J). Anterior populations–including forebrain, midbrain, and hindbrain–were strongly linked, while posterior spinal cord populations generated during axial elongation^15,59,60^ occupied a distinct region of the map. Differentiated neurons clustered according to their regional identity, with spinal cord inter and motor neuron subtypes further organizing according to dorsal-ventral (D-V) position along the axis from floor to roof plate (fig. S5D).

Mesodermal cell types segregated into two distinct blocks: an anterior block comprising head, pharyngeal, and cardiac mesoderm, and a posterior block containing lateral plate and paraxial derivatives including dermomyotome and sclerotome (Fig. 3J). Endothelial cell types were associated with both the anterior and posterior clusters, reflecting their broad mesodermal origin and subsequent diversification into region-specific subtypes^61^. Further, among anterior cell types, ancestral linkage organizes paraxial, intermediate, and lateral plate derivatives along the mediolateral axis. Notably, the most posterior mesodermal populations–presomitic mesoderm and nascent somites–were more strongly linked to axial progenitors and neural ectoderm than to other mesodermal cell types, consistent with axial progenitors contributing to both neural and mesodermal lineages during axis elongation^59,60^.

Surface ectoderm and neural crest each formed discrete yet tightly associated clusters (Fig. 3J). Neural crest cell types also exhibited close linkage to the neural ectoderm, consistent with its origin at the boundary between neural and surface ectoderm in the developing neural plate^62–66^. These relationships were reinforced at the level of individual cell types: roof plate cells clustered with neural crest, reflecting their spatial proximity to the neural plate border, and sacral neural crest grouped with posterior, axial progenitor-derived ectodermal populations. Within the surface ectoderm, sensory neurons clustered tightly with their associated placodal populations, consistent with recent reports of their shared ancestry^67^.

Finally, a subset of cell types showed minimal off-diagonal linkage (Fig. 3J). Endodermal cell types–including foregut, midgut, hindgut, hepatoblast, and enteroendocrine progenitors–formed a cohesive cluster organized along the A-P axis with minimal off-diagonal signal, in line with the early lineage separation observed in our germ layer analyses. Notably, notochord cells exhibited linkage to both endoderm and floor plate, despite their classification as mesoderm, reflecting their unique developmental origin and signaling role.

### Quantification of cell fate specification

We next sought to resolve how cell fate specification unfolds over time by asking when fate biases toward specific cell types first emerge, when these fates become restricted, and the size and number of distinct clades contributing to each cell type.

To determine when cell type fates begin to diverge, we calculated fate distributions for ancestral nodes in our E9.5 lineage trees and quantified deviations from the background distribution over time. Similar to our germ layer analyses, significant fate bias first emerged at ~E4.0, a result we corroborated using a cell type label-independent measure of transcriptional distance (figs. S7E,F). To dissect the drivers of these early biases, we clustered early ancestral nodes by the fate distribution of their descendants and identified 15 recurrent fate bias motifs present before E7 (figs. S7G–J). These motifs ranged from a minimally biased, early-arising motif to one specific to the telencephalon and eye field (figs. S7K,L). The earliest detectable biases were driven primarily by mesodermal fates and some anterior ectodermal and neural crest cell types (fig. S7M).

Having defined when fate biases first emerged, we next quantified when these biases resolve into fate-restricted clades, how many clades contribute to each cell type, and the overall output of these clades. Among the earliest fate-restricted cell types were extraembryonic-derived amniotic ectoderm, megakaryocytes, and macrophages, as well as primordial germ cells, which despite their embryonic origin are known to arise as a specific population within the posterior embryo at the earliest stages of epiblast patterning (Figs. 3K–M)^68,69^. Because of their early specification, these populations showed minimal linkage to other developmental domains (Fig. 3J).

Across all metrics, cell fate determination dynamics were highly consistent across triplicate E9.5 embryos, despite each embryo comprising independent clonal populations with distinct founding cells. We observed similar cell type abundances and use of early fate bias motifs across independent embryos (figs. S7N–Q). This reproducibility indicates that the programs driving cell type fate restriction are consistent features of mammalian development. Together, these analyses provided a quantitative framework for cell fate specification and motivated a closer examination of specific structures within the developing embryo. Below, we provide a more in-depth examination of the notochord, heart, and neural crest.

### Early fate restriction of the notochord

**Fig. 4.**
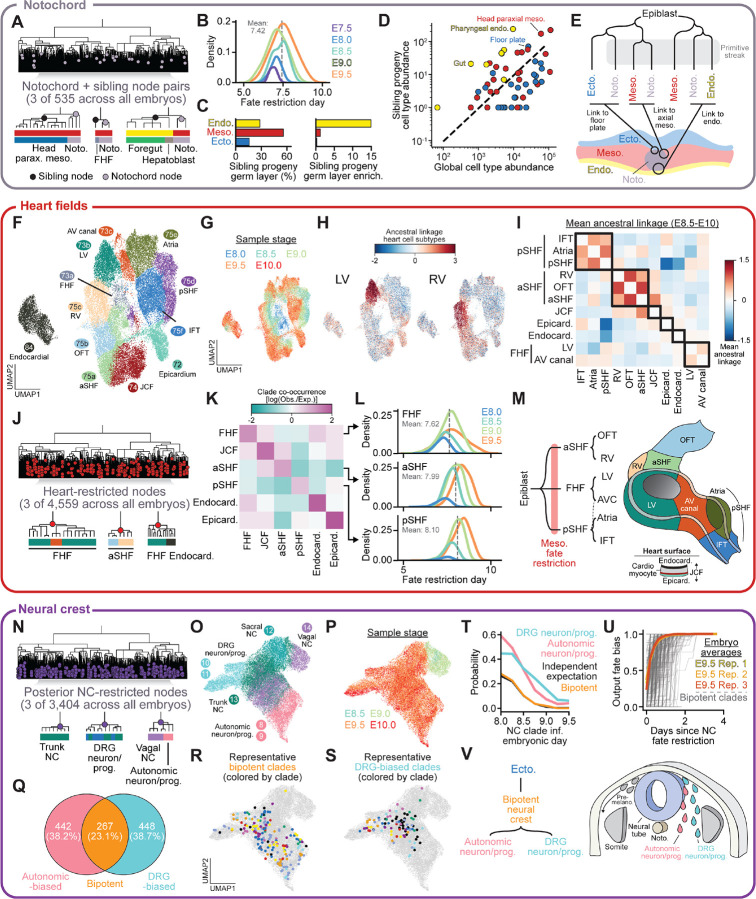
Cell fate specification dynamics in the developing notochord, heart fields, and neural crest. (**A-E**) Notochord: (**A**) Example lineage tree (E9.5 replicate 2, clone 1; E9.5-R2-C1) with notochord-restricted ancestral nodes marked (grey circles). Inset trees depict 3 of 535 annotated notochord sibling pairs across all embryos. Color bars beneath these trees indicate the cell type identity of descendant cells from notochord-restricted (grey circles) and sibling (black circles) ancestral nodes. (**B**) Distribution of inferred notochord fate restriction timing across triplicate sampled timepoints (E7.5-E9.5). Dashed line indicates the mean. (**C**) Left, germ layer composition of notochord sibling clades. Right, fold enrichment of germ layer representation relative to expected background. (**D**) Sibling clade cell type abundance versus global cell type abundance. Points above the diagonal are enriched among notochord sibling clades; selected cell types are labeled. Colors indicate germ layer. (**E**) Model of notochord development. Notochord clades are frequently specified directly from the epiblast, with sibling clades adopting alternative fates prior to and during primitive streak ingression. (**F-M**) Heart fields: (**F**) UMAP of 12 annotated cardiac cell subtypes from E8.0-E10.0 timepoints colored by cell type, (**G**) sample stage, and (**H**) ancestral linkage to the left ventricle (LV) and right ventricle (RV) for all annotated heart cells. FHF = first heart field, AV canal = atrioventricular canal, aSHF = anterior second heart field, pSHF = posterior SHF, IFT = inflow tract, JCF = juxtacardiac field, OFT = outflow tract. (**I**) Mean pairwise ancestral linkage across cardiac cell types (E9.0-E10.0; see figs. S8G,H for E8.0 and E8.5). Black boxes highlight FHF, aSHF, and pSHF heart fields. Epicard. = epicardium, Endocard. = endocardial cells. (**J**) Example lineage tree (E9.5-R2-C1) with heart-restricted nodes (red circles) marked. Inset trees depict 3 of 4,559 heart-restricted nodes across all embryos. Color bars indicate cell type identity of descendant cells. (**K**) Heart field co-occurrence [log(observed/expected)] within heart-restricted clades across sampled timepoints (E8.0-E9.5). (**L**) Distribution of inferred FHF, aSHF, and pSHF fate restriction timing across sampled timepoints (E7.5-E9.5). Dashed line indicates the mean. (**M**) Model of heart cell type fate restriction. FHF, aSHF, and pSHF are sequentially restricted from distinct pools of mesodermal progenitors. (**N-V**) Neural crest (NC): (**N**) Example lineage tree (E9.5-R2-C1) with posterior NC-restricted ancestral nodes (purple circles) marked. Inset trees depict 3 of 3,404 annotated nodes across all embryos. Color bars beneath these trees indicate the cell type identity of descendant cells. DRG = dorsal root ganglia, prog. = progenitor. **(O)** UMAP of posterior NC cells colored by cell type and (**P**) sample stage. (**Q**) Venn diagram of posterior NC-restricted clades producing DRG-biased, autonomic-biased, or bipotent outputs at E9.5. (**R**) Representative bipotent and (**S**) DRG-biased trunk NC clades shown on the UMAP in (O). 10 colors indicate 10 randomly sampled clades. (**T**) Probability of an E9.5 clade containing DRG, autonomic, and bipotent outputs as a function of NC-restricted clade depth (inferred embryonic day). Black line indicates the expected bipotent frequency under statistical independence between DRG and autonomic fate occurrence. (**U**) DRG versus autonomic fate bias by time since NC fate restriction for individual bipotent clades (gray) and averaged across E9.5 replicates (colored). (**V**) Model of bipotent trunk NC cells resolving to DRG or autonomic fates.

The notochord is a transient axial structure that forms along the embryonic midline during gastrulation and functions as a key signaling center for dorsal-ventral patterning of the overlying neural tube and adjacent paraxial mesoderm^70^. In agreement with its early developmental role, the notochord was among the earliest specified cell types in our dataset (Fig. 3M). The average time of notochord fate restriction was before E7.5 across all triplicate embryonic timepoints, often concurrent with the initial specification of mesodermal fate (Figs. 4A, B, fig. S8A). To understand the developmental origins of notochord cells, we examined the sibling clades of notochord-restricted nodes in our lineage trees and quantified their outputs (Fig. 4A). The majority of sibling descendants were mesodermal (55.8%), but notably 28.4% were endodermal, a 15.8-fold enrichment relative to their overall frequency (Fig. 4C). This relationship is further supported by the transcriptional similarity between these populations in the E7.5 to E8.0 window, as exemplified by shared *FoxA2* expression (figs. S8B,C). Indeed, classical fate maps show that definitive endoderm and notochord arise from the anterior primitive streak^10,71^, presumably explaining the clonal relationships observed here. Moreover, notochord fate restriction timing was not correlated with sibling germ layer fate, suggesting that these relationships reflect spatial associations in the epiblast, rather than differences in the timing of specification among notochord subpopulations (fig. S8D).

At the cell type level, sibling clade outputs were enriched for gut and paraxial mesoderm, tissues that develop alongside the notochord at the embryonic midline (Fig. 4D). Notably, among ectodermal outputs of sibling clades (15.8% of all sibling descendants), the predominant cell type was floor plate, which emerges within the ventral neural tube in close spatial proximity to the notochord. Together, these data support a model in which the notochord is often specified directly from the epiblast, with shared ancestry across germ layers reflecting the alternative fates taken by sibling descendants during and after primitive streak ingression according to their spatial position (Fig. 4E). The complex origins of the notochord observed here are consistent with historical studies documenting its clonal relationships with both the definitive endoderm and floor plate^72,73^.

### Lineage architecture of the developing heart

To resolve lineage relationships within the developing heart, we annotated 12 cardiac cell subtypes spanning the major cardiac compartments from E8.0 to E10.0 (Figs. 4F,G, fig. S8E). These annotations capture both early progenitor states and more differentiated populations, enabling resolution of how cardiac cell types emerge and diversify throughout development. To understand lineage relationships between cardiac cell types, we computed ancestral linkage between each annotated population and all other heart cells. Notably, these analyses uncovered lineage relationships between cardiac cell types that were not apparent from transcriptional data alone; for example, despite shared cardiomyocyte programs (*e.g., Myh7*, *Myl3* expression), right ventricular (RV) and left ventricular (LV) populations are not closely related (Fig. 4H, figs. S8E,F), matching their origins from the second and first heart fields, respectively^74,75^.

Mean pairwise ancestral linkage between all cardiac cell types resolved the first heart field (FHF) and two distinct second heart field (SHF) populations–anterior SHF (aSHF; also called the anterior heart field) and posterior SHF (pSHF) (Fig. 4I, figs. S8G,H)^76–79^. Beyond these heart fields, we also recovered lineage relationships between other cardiac cell types. For example, atrioventricular canal (AV canal) cells showed strong linkage to both FHF and pSHF, reflecting their position at the atrial-ventricular boundary^80^. In contrast, epicardial and endocardial populations were largely segregated, with epicardium retaining some linkage to inflow tract-associated lineages, in line with its origin from the proepicardium (PE) at the base of the cardiac inflow tract^81^.

The early lineage separation of the aSHF and pSHF motivated a more detailed analysis of the 4,559 heart-restricted clades in our dataset. We quantified the degree of heart field co-occurrence across clades relative to expectation (Methods) and observed minimal overlap in the clades contributing to aSHF and pSHF (Figs. 4J,K, fig. S8I). These heart fields were also sequentially fate-restricted, with FHF clades becoming specified earliest, followed by aSHF and then pSHF (Fig. 4L). Notably, when we computed clade co-occurrence at the level of mesoderm-restricted ancestral nodes–where mesodermal fate specification precedes that of the heart fate by approximately one day–we again observed minimal overlap between aSHF and pSHF (figs. S8J–L). Together, these data align with conventional marker-driven lineage tracing results demonstrating the early emergence of the FHF and that aSHF and pSHF construct distinct parts of the heart^82,74,75^.

Given that cardiac lineage relationships are established early and maintained through heart morphogenesis, we asked whether additional lineage-related structure defines sub-domains within individual cardiac cell types. Indeed, for 38.7% of heart-restricted clades, descendant cells were significantly more transcriptionally similar to one another than randomly sampled cells from the same cell type, revealing an array of sub-domains nested within broader cardiac cell type identities (figs. S8M,N). Many transcriptionally diverse clades contained one or more endocardial cells, in agreement with the emergence of this cell type from multiple heart fields^83^ (figs. S8O,P). Overall, 68.7% of endocardial cells derived from endocardial-biased clades while the rest derived from clades biased toward other heart fields, particularly the FHF (figs. S8P,Q). Together, these data support a model in which the distinct lineage identities of cardiac populations are established alongside mesodermal fate restriction, well before transcriptionally-defined heart cell types are detected (Fig. 4M).

### Fate restriction in the neural crest

The neural crest is a transient, migratory population of multipotent progenitors that contribute to diverse embryonic structures, including the peripheral nervous system, cardiovascular system, and the craniofacial skeleton^84,85^. To understand how these multipotent cells become restricted to specific fates, we identified 11,622 neural crest-restricted ancestral nodes and quantified cell type co-occurrence across the descendant clades (fig. S9A). As with many cell types that emerge along the anterior-posterior axis, we observed clear separation between cranial and posterior neural crest (vagal, trunk, and sacral) populations (fig. S9B)^86^.

Fate restriction dynamics within the posterior neural crest–particularly the trunk neural crest, which gives rise to both sensory and autonomic (sympathetic and parasympathetic) outputs–provided a framework to examine how these distinct fates are specified. Focusing on the 3,404 posterior neural crest-restricted clades, we resolved differentiated dorsal root ganglion (DRG) and autonomic neurons alongside their progenitors (Figs. 4N–P). Vagal, trunk, and sacral neural crest showed sequential fate restriction (mean E8.19, E8.47, and E8.70, respectively), reflecting progressive specification along the anterior-posterior axis (fig. S9C)^64,65^. Clades from early-sampled embryos were predominantly composed of *Sox10*^+^ neural crest progenitors, while E9.5 clades additionally contained DRG and autonomic outputs (figs. S9D–H). Trunk neural crest clades contained both DRG and autonomic cells, whereas vagal and sacral clades were enriched for autonomic outputs.

We next classified E9.5 posterior neural crest clades as DRG-biased, autonomic-biased, or bipotent (both DRG and autonomic) based on the composition of descendant cells (fig. S9I). These annotations represent observed fates at E9.5 rather than developmental potential, as the remaining *Sox10*+ progenitors within these clades may differentiate into DRG, autonomic, or melanocyte cells later in development. Clades containing differentiated outputs were split equally between DRG- and autonomic-biased, with 23.1% of clades being bipotent (Figs. 4Q–S). The probability of a clade containing either DRG or autonomic outputs decreased with later restriction time, likely reflecting progressive neural crest specification and differentiation along the A-P axis (Fig. 4T). Notably, the frequency of bipotent clades closely tracked what would be expected from statistically independent occurrence of DRG and autonomic fates. However, once differentiation began, subclades rapidly resolved to either DRG or autonomic fate (Fig. 4U). We observed similar trends for cranial neural crest fate restriction to mesenchymal and trigeminal outputs (figs. S9J–V). Together, these data are compatible with a model in which restriction to DRG and autonomic fates is not pre-specified at the time of trunk neural crest fate specification, but resolves over the course of their migration (Fig. 4V)^64^. However, since delamination is not directly measured, the precise timing of fate restriction relative to this process cannot be determined.

### Gene expression drivers of cellular diversification

**Fig. 5.**
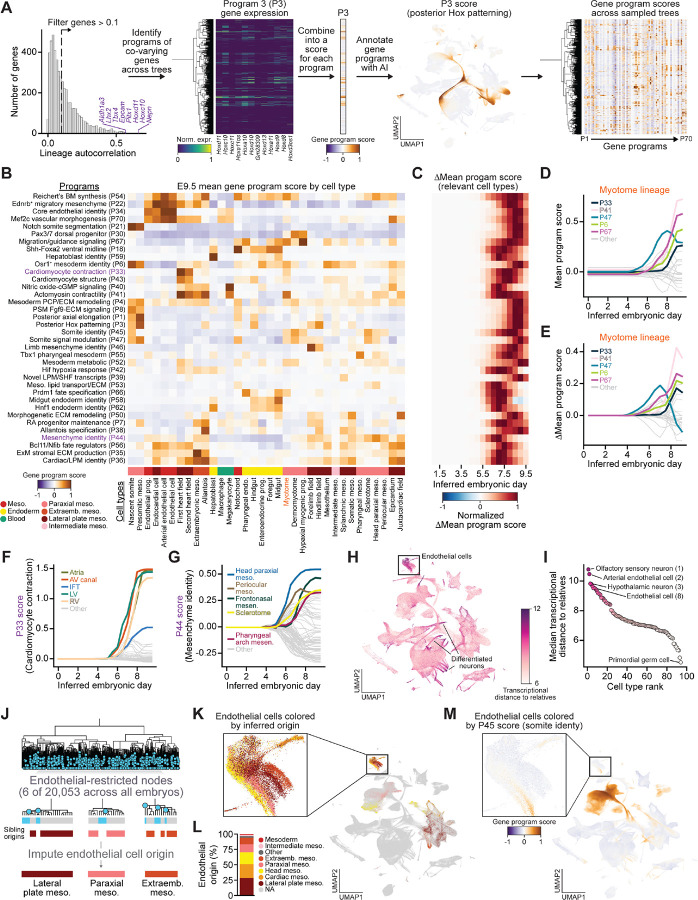
Gene regulatory programs underlying cell fate specification. (**A**) Overview of gene program identification process. Far left: Genes exhibiting heritable expression (scaled local autocorrelation >0.1; dashed line) were identified and grouped into 70 coherent programs based on pairwise local autocorrelation (Hotspot; DeTomaso and Yosef, 2021). Middle left: Normalized (norm.) gene expression for genes grouped into program 3 (P3, posterior Hox patterning) are shown. Middle: P3 score across all cells across an example lineage tree (E9.5-R2-C3) and Middle right: UMAP of sampled cells colored by P3 score. Far right: All 70 program scores visualized on an example lineage tree (E9.5-R2-C3). **(B)** Mean score for each program (rows) across mesodermal and endodermal cell types (columns) in E9.5 embryos. Cell type developmental domain assignments are indicated by colored bars along the bottom. Highlighted programs in purple: P33 (cardiomyocyte contraction) and P44 (mesenchyme identity). Highlighted cell type in orange is myotome. For a full list of gene regulatory program names, abbreviations, and descriptions of their assignment see Table S3. **(C)** Change in mean program score (ΔMean program score) over inferred embryonic day in E9.5 lineage trees for relevant cell types (mean >0.5 in at least one timepoint), normalized per program. Programs are ordered as in (B). **(D)** Mean program score and (**E**) change in mean program score over inferred embryonic day for selected programs within the myotome lineage. Programs include: P33 (cardiomyocyte contraction), P41 (actomyosin contractility), P47 (somite signal modulation), P6 (Osr1+ mesoderm identity), and P67 (migration/guidance signaling). **(F)** P33 (cardiomyocyte contraction) program score and (**G**) P44 (mesenchyme identity) program score by inferred embryonic day across all cell types (grey). Cardiac cell types are colored in (F); head paraxial mesoderm, periocular mesoderm, frontonasal mesenchyme, sclerotome, and pharyngeal arch mesenchyme colored in (G). All other cell types colored in grey. **(H)** UMAP of all sampled cells colored by mean transcriptional distance to their closest relatives (most recent common ancestor, MRCA within 1.5 days) in reconstructed trees with endothelial cells and differentiated neurons are labeled. **(I)** Mean transcriptional distance to relatives ranked across all cell types, with points colored by the same metric. **(J)** Model of endothelial origin imputation. Example lineage tree (E9.5-R2-C1) with endothelial-restricted ancestral and leaf nodes marked (light blue circles). Inset trees depict 6 of 20,053 fate-restricted nodes along with their siblings. The cell type composition of siblings is used to infer the developmental origin of each endothelial cell (e.g., lateral plate, paraxial, or extraembryonic mesoderm). **(K)** UMAP of all sampled cells with endothelial cells and their siblings colored by inferred developmental origin with inset showing endothelial cells. **(L)** Proportion of endothelial cells assigned to each inferred developmental origin, including lateral plate, intermediate, paraxial, extraembryonic, head, and cardiac mesoderm. **(M)** UMAP and inset from (K) colored by P45 (somite identity) program score, showing retention of somite identity signatures among paraxial mesoderm-derived endothelial cells.

Having resolved the lineage architecture underlying cell fate specification, we next sought to understand the gene regulatory programs that drive these transitions. We identified genes with heritable expression patterns and used Hotspot^87^ to detect lineage-associated covariation in expression and to group genes into 70 coherent programs based on pairwise local autocorrelation (Fig. 5A, fig. S10A; Methods). These programs–which were annotated using an AI-assisted approach based on their component genes and expression patterns–captured major axes of developmental regulation, including spatial patterning, cell type specification, and signaling responses (Fig. 5A, figs. S10B,C). We assessed gene program activity across all cells and timepoints and imputed activity at ancestral nodes (Methods), enabling reconstruction of program dynamics along lineage trajectories, where node scores reflect the average activity of their descendant cells (Figs. 5B,C, fig. S10, fig. S11).

These analyses revealed two complementary modes of gene program activity: lineage-restricted deployment within individual cell types and recurrent activation across cell types. Within certain cell types, gene programs were activated in defined temporal sequences. For example, in the myotome, somite signal modulation (P47) was activated earliest, followed by actomyosin contractility (P41) (Fig. 5D). Changes in mean program score highlighted these shifts in program utilization across inferred embryonic time (Fig. 5E).

Across cell types, some programs were activated within specific lineages and deployed in a temporally ordered manner. For example, the cardiomyocyte contractility program (P33) was activated across cardiac cell types, with earliest activation in the FHF, followed by SHF derivatives as expected from our estimates of sequential heart field specification described above (Fig. 5F; see Figs. 4F–M). In contrast, other programs were reused across developmentally distinct but functionally related cell types. The mesenchymal program (P44), for example, was deployed across multiple mesenchymal populations, with early activation in head paraxial and periocular mesoderm followed by later activation in frontonasal mesenchyme, sclerotome, and pharyngeal arch mesenchyme (Fig. 5G). Similar patterns were observed across ectodermal lineages, including rapid activation of neuronal specification, differentiation, and maturation programs (figs. S10D–G).

The rapid activation of neuronal programs suggested that some cell types undergo sharp transcriptional transitions over short developmental intervals, including the activation of convergent functional programs from independent progenitor pools. To quantify this phenomenon across all cell types, we measured the mean transcriptional distance between cells and their closest relatives in reconstructed trees (Fig. 5H). Using this framework, we saw clear transcriptional divergence of neural subtypes from their progenitors, as well as notable signal from endothelial cells, which must be deployed across the entirety of the developing embryo to establish the vasculature (Fig. 5I).

### Convergent dynamics of endothelial fate induction

Prompted by this observation, we identified 20,053 endothelial-restricted clades across all trees and inferred their developmental origins by examining the sibling clades of each fate-restricted node (Fig. 5J). Endothelial cells arose from across the mesoderm as previously reported in classical lineage tracing studies (Figs. 5K,L)^64,88^. Notably, a substantial fraction of endothelial cells derived from paraxial mesoderm, confirming a long-held hypothesis that somitic (paraxial) mesoderm can also produce endothelial cells (Figs. 5K,L)^89–92^. Although endothelial cells from diverse mesodermal sources converged on a shared core endothelial program (Fig. 5B), they retained detectable transcriptional signatures of their tissue of origin. For example, paraxial mesoderm-derived endothelial cells maintained residual expression of the somite identity program (P45) (Fig. 5M). Together, these results indicate that while multiple mesodermal progenitors give rise to endothelial cells, each subtype retains a transcriptional memory of its distinct ancestral mesodermal origins.

### Spatiotemporal patterning of the neural ectoderm

**Fig. 6.**
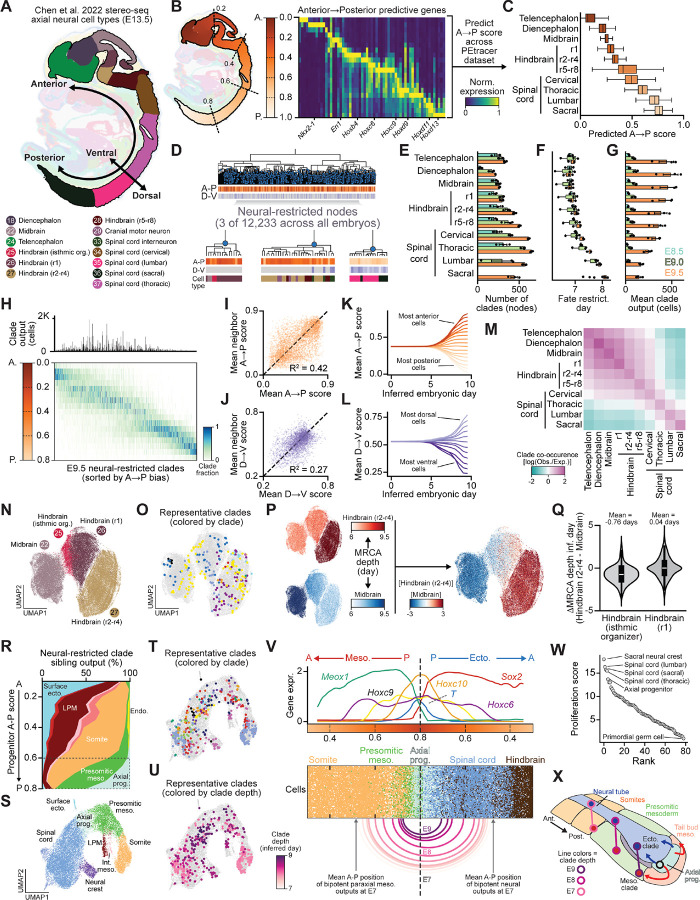
Establishment of spatial axes, regionalization, and axis elongation. (**A**) Spatial transcriptomics data from an E13.5 mouse embryo cross-section (Stereo-seq; Chen et al., 2022). Neural ectoderm is outlined and colored by axial region, while other tissues are shown in the background for reference. Cell type colors and numbers correspond to Fig. 2. Anterior-posterior (A-P) and dorsal-ventral (D-V) axes are indicated. Org. = organizer. (**B**) Left: Cross-section from (A) with neural ectoderm colored by A-P position. Right: Expression along the A-P axis of 61 patterning genes used to predict A-P position. (**C**) Predicted A-P score distribution for axial regions. Boxes indicate mean and interquartile ranges. Colors correspond to mean A-P score for each region. (**D**) Example lineage tree (E9.5-R1-C1) with neural-restricted nodes (blue circles) marked. Inset trees depict 3 of 12,233 neural-restricted nodes across all embryos. Color bars indicate A-P score, D-V score, and cell type identity of descendant cells. (**E**) Number of neural-restricted clades contributing to each region by sampled timepoint (E8.5-E9.5). (**F**) Mean fate restriction day for neural-restricted clades by region. (**G**) Mean number of descendant cells per clade for each region. For (E-G), bars represent the mean of three biological replicate embryos ± standard error of the mean. (**H**) Heatmap showing the distribution of A-P positions (rows) for neural-restricted clades in E9.5 embryos with >10 descendants (columns), ordered by mean A-P score. Clade output (number of cells) shown above. (**I**) Mean A-P score of E9.5 neural-restricted clades versus that of their nearest neural-restricted lineage neighbor. Dashed line shows y = x. (**J**) Same as (I) for D-V score of spinal cord clades. (**K**) Mean A-P score of ancestors over inferred time for neural cells grouped and colored by their observed A-P position at E9.5. (**L**) Same as (K) for D-V score of spinal cord cells. (**M**) Axial region co-occurrence [log(observed/expected)] within neural-restricted clades at E9.5. (**N**) UMAP of E9.5 midbrain, hindbrain (isthmic organizer), hindbrain (r1), and hindbrain (r2-r4) cells colored by cell type and (**O**) clade where the 10 colors indicate 10 randomly sampled clades. (**P**) Left: Inferred time (day) of the most recent common ancestor (MRCA) between each cell and hindbrain (r2-r4) cells (top) or midbrain cells (bottom). Right: Difference (Δ) between these values, showing relative lineage relatedness. (**Q**) Distribution of ΔMRCA depths (hindbrain r2-r4 minus midbrain) for hindbrain isthmic organizer and hindbrain (r1) cells. (**R**) Sibling clade output composition for E9.5 neural-restricted clades, ordered by mean A-P score (y-axis). Colors denote developmental domain of sibling descendants with paraxial mesoderm split into somitic and presomitic groups. Dashed box indicates the posterior region expanded in (S-V). (**S**) UMAP of posterior neural-restricted clades and their siblings colored by developmental domain from (R). (**T,U**) Representative neural-restricted clades with sibling outputs, (**T**) colored by clade identity and (**U**) colored by clade depth (inferred day). (**V**) Top: Gene expression profiles of regional markers along the A-P axis showing the transcriptional continuum from somite (*Meox1*^+^), through presomitic mesoderm and *T*^+^ axial progenitors, to spinal cord (*Sox2*^+^) and hindbrain cells. Middle: Cells colored by developmental domain as in (S), ordered by A-P position. Bottom: Bipotent clades grouped and colored by depth (E7-E9) with arcs indicating the mean A-P position of mesoderm and ectoderm outputs for each group. Dashed line indicates the posterior end of the embryo. (**W**) Proliferation scores (calculated as mean number of cells sharing a common ancestor within the previous 24 hours of inferred time) ranked across cell types in the E9.5 dataset. Selected cell types with high and low proliferation scores are labeled. (**X**) Model of axial progenitor dynamics during tailbud elongation. Bipotent clades (outlined circles; lines colored by depth) give rise to descendants where mesodermal (red) or ectodermal (blue) progeny follow distinct paths in the growing tailbud, producing a consistent spatial offset between neural and mesodermal outputs.

Spatial patterning is a central driver of cell type specification in the developing mouse embryo, particularly within the neural ectoderm, which generates diverse neuronal populations along the A-P and D-V axes^63,93,94^. To understand the interplay between spatial position and cell fate, we used published spatial transcriptomics data to identify spatially predictive genes (predominantly Hox family genes) and inferred each cell's A-P position from their expression (Figs. 6A,B, fig. S12A–F; Methods)^95–97^. Further, because D-V patterning is well resolved in the spinal cord^98^, we applied an analogous approach to infer D-V positions in this region (figs. S12G–L). These inferred coordinates recapitulated established spatial organization of cell types across the developing brain and spinal cord (Fig. 6C, fig. S12M).

To understand how neural lineage specification varies along the A-P axis, we identified 12,233 neural-restricted clades across all embryos (Fig. 6D). The number of clades, their mean depth in the tree, and their output varied systematically with A-P position (Figs. 6E–G). Anterior brain regions–including the telencephalon, diencephalon, and midbrain–were seeded around E7.0 by a relatively small number of early-restricted clades whose output expanded substantially over time^99^. In contrast, posterior spinal cord populations continued to acquire new fate-restricted clades throughout development^15^. These patterns suggest two distinct modes of neural expansion: anterior growth driven by clonal amplification of a small number of high-output, early-specified progenitors, and posterior growth sustained by ongoing specification of a large number of lower-output clades from axial progenitors during tailbud elongation (Figs. 3J, 6H). These analyses quantify the distinct developmental origins of anterior and posterior CNS populations^100,59,101^.

We next examined how anterior and posterior clades differ in their spatial distribution throughout the developing embryo. In E9.5 lineage trees, anterior clades tended to span broader A-P domains and contribute to multiple regions, in line with their early specification, whereas posterior clades were restricted to more limited spans along A-P axis (Fig. 6H, fig. S13A,B). Within the spinal cord, clades were often biased toward specific D-V positions, although the within-clade span along the D-V axis was often greater than along the A-P axis (fig. S13C). Clade specification timing showed weak positive correlation with the mean position of descendants along the A-P axis (R^2^ = 0.21), but not with D-V position (R^2^ = 0.01) (figs. S13B,D).

Finally, we assessed the heritability of spatial identity across E9.5 lineage trees by comparing the mean spatial position of each clade to that of its nearest neighbor in the tree (Figs. 6I,J). A-P position showed substantial heritability (R^2^ = 0.42), while D-V position was less strongly correlated (R^2^ = 0.27). To determine when spatial biases first arose, we inferred the positions of ancestral nodes based on the mean spatial scores of their descendants. A-P bias emerged ~E5.0, with ancestors of cells in the most anterior and posterior regions already beginning to skew towards those directions (Fig. 6K). D-V bias arose at a similar time; however, unlike the symmetric pattern observed along the A-P axis, ventral bias was initially more pronounced than dorsal (Fig. 6L). This asymmetry is consistent with neurulation, in which ventral positions are specified earlier at the base of the neural groove^102^. Together, these results indicate that spatial organization is established prior to neural fate specification, particularly along the A-P axis.

### Regionalization of the neural ectoderm

The neural ectoderm is organized along the A-P axis into morphologically and transcriptionally defined domains, including the telencephalon, diencephalon, midbrain, hindbrain rhombomeres, and spinal cord^103^. We quantified how often neural-restricted clades contribute cells across molecularly distinct brain regions (Fig. 6M). Clades frequently spanned multiple regions, including across the forebrain-midbrain and midbrain-hindbrain boundaries. This extensive overlap is consistent with the broad A-P span of anterior clades and indicates that lineage relationships do not strictly follow regional boundaries.

We next asked whether spatially and transcriptionally defined brain regions are nonetheless defined by cell lineage despite this lack of clear clade-level restriction. We focused on the first rhombomere (r1), a well-defined interface between midbrain and hindbrain that can be distinguished from the adjacent *Otx2*^+^ midbrain, more posterior *Hoxa2*^+^ rhombomeres (r2-r4), and the *En1*^+^ and *Fgf8*^+^ isthmic organizer at the midbrain-hindbrain boundary (Fig. 6N, figs. S13E–H)^104–106^. Since many neural-restricted clades span r1, we asked when r1 cells become lineage-restricted relative to adjacent regions (Fig. 6O). We inferred the timing of each r1 cell’s most recent common ancestor (MRCA) with either midbrain or hindbrain (r2-r4) cells, and used the difference between these times as a measure of relative lineage separation (Fig. 6P). Cells within the isthmic organizer were more closely related to the midbrain, whereas cells across the remainder of r1 showed comparable lineage relationships to both midbrain and posterior hindbrain regions with a gradient corresponding to A-P position (Figs. 6P,Q). Lineage relationships between adjacent regions persist until approximately E8.0 (fig. S13I), consistent with regional identity being established from a progenitor field based on A-P position rather than early specification of region-specific progenitors, even at the midbrain-hindbrain boundary.

### Identification of bipotent axial progenitors

The neural tube is generated by two distinct developmental mechanisms along the A-P axis: anterior regions are formed via primary neurulation, in which the neural ectoderm folds and closes, whereas the posterior neural tube is generated through secondary neurulation from axial progenitors during axis elongation^15,59,101^. To characterize how these modes of neurulation cooperate to construct the neural A-P axis, we analyzed the sibling clades of neural-restricted nodes and quantified their outputs at E9.5 (Fig. 6R). Anterior sibling clades contributed to all three germ layers, with an enrichment for surface ectoderm, in agreement with an epiblast origin. In contrast, posterior sibling clades were enriched for both paraxial mesoderm and axial progenitors, supporting the presence of bipotent progenitors in the growing tailbud^59,60,107^. Consistent with this observation, cells sharing a recent ancestor with the caudal neural ectoderm formed a transcriptional continuum from *Sox2*^+^ neural ectoderm to *Meox1*^+^ paraxial mesoderm, with *T*^+^ axial progenitors positioned at the interface (Fig. 6S, figs. S13J–M).

We next examined the lineage relationships among these posterior embryonic outputs. Many clades contained both ectodermal and mesodermal descendants, and cells in more recent clades were often more transcriptionally similar to axial progenitors, linking bipotent clades to the *T*^+^ axial progenitor state (Figs. 6T,U). To determine when ectodermal and mesodermal fates diverge along the A-P axis, we calculated the fraction of neural cells that have a common ancestor with the mesoderm at different tree depths (E7.0-E9.0). Posterior neural cells were much more likely to have recently diverged from the mesoderm, with 58% of the most posterior cells sharing an ancestor at E8.0 (fig. S13N). Among these posterior bipotent clades, 42% contained an axial progenitor at E9.5, with the remainder likely reflecting progenitors that have fully differentiated into ectoderm and mesoderm or incomplete sampling (fig. S13O).

Given that axial progenitors are maintained at the posterior end of the embryo throughout axis elongation (fig. S13P), we reasoned that the A-P position of bipotent clades should correlate with their depth in the lineage tree. We therefore applied our A-P scoring framework to the paraxial mesoderm, where these scores reflect Hox gene expression rather than absolute anatomical position, given that Hox expression domains in the mesoderm are modestly shifted relative to those in the neural tube^108^. Consistent with our prediction, bipotent clades were ordered along the A-P axis according to their depth within the lineage tree (Fig. 6V), with more anteriorly positioned cells generated by earlier differentiation events. Moreover, these dynamics closely paralleled the *Sox2*^+^:*T*^+^:*Meox1*^+^ transcriptional continuum described above. Within individual clades, mesodermal descendants showed slightly more posterior Hox identities than their ectodermal siblings (Fig. 6V, fig. S13Q).

Finally, we examined how axial progenitors sustain posterior growth despite their relatively small population size. We estimated proliferation rates using the number of close relatives in the lineage tree, defined as cells sharing a common ancestor within the previous 24 hours (fig. S13R). Axial progenitors and posterior neural populations exhibited the highest proliferation scores–more than an order of magnitude greater than those of primordial germ cells, which had the lowest rates within the embryo during this interval (Fig. 6W).

Together, these findings support a model in which highly proliferative bipotent axial progenitors are maintained at the posterior end of the embryo and continuously generate both neural and mesodermal lineages during axis elongation, explaining the recent shared ancestry in our global analysis (Figs. 3J, 6X)^59,60,107^. In this model, progenitors progressively acquire posterior Hox identities while residing in the tailbud, and their descendants inherit these identities upon exit from the posterior progenitor pool. Neural and mesodermal derivatives diverge from this shared pool with distinct temporal dynamics: neural descendants tend to exit earlier and differentiate within the neural tube, whereas mesodermal descendants remain in the posterior growth zone longer than neural descendants before contributing to the presomitic mesoderm. This temporal asymmetry provides a parsimonious explanation for the consistent offset in Hox identity and A-P position between neural and mesodermal lineage outputs, as has been observed in mouse and zebrafish fate-mapping studies^15,109–111^.

## Discussion

Here we map cell fate specification from pre-gastrulation through early organogenesis in the mouse by integrating cell state and lineage measurements at organismal scale. Using PEtracer to continuously install heritable lineage marks, we reconstruct lineage trees for 16 embryos comprising >1.5 million cells sampled at half-day intervals from E7.5 to E10.0. We resolved approximately 75% of all divisions throughout this developmental window (>2.1 million cell divisions)–enabling measurement of the timing, trajectories, and reproducibility of fate restriction. Near-complete cellular sampling preserves the structure of lineage trees and captures how cell fate decisions unfold throughout development, including rare and late fate restriction events, while deep transcriptional profiling links lineage architecture to precise cell type identities. Together, these data represent a key step toward a comprehensive fate map of mouse embryogenesis.

The combined scale and density of this dataset provide a framework for generating hypotheses and contextualizing more focused studies of fate restriction dynamics in mouse development. Classic fate-mapping approaches have provided deep, mechanistic insight into specific lineages and remain foundational for defining progenitor potential and tissue relationships^17,112,113^. More recent barcoding and lineage recording methods have extended these approaches, enabling broad coverage across tissues and developmental time, but are often limited by subsampling, restricting analysis to aggregate lineage relationships^33,2,114,39^. By reconstructing densely sampled, high-resolution lineage trees that resolve the majority of cell divisions in profiled cells, we directly quantified progenitor-progeny relationships across embryogenesis without requiring prior knowledge of lineage-defining markers or when to introduce lineage barcodes. Each internal node represents an inferred ancestral cell at a defined time, allowing us to infer the timing of fate bias and restriction. The near-comprehensive nature of these measurements allows us to quantify how cell types emerge, including number of contributing clades, their output, and the lineage relationships between cell types. Within each embryo, we captured many instances of each cell fate decision, providing the full distribution of outcomes in the context of concurrent developmental programs. Further, our ability to generate replicate trees at each timepoint using the same cell line provides controlled empirical estimates of inter-embryo reproducibility. These data enable characterization of each fate decision by its frequency, consistency, and variance across independently derived embryos and create a unified view of embryogenesis and elucidate key principles of fate specification.

First, we find that the overall lineage architecture is highly reproducible across embryos. Despite the well-established non-deterministic nature of mammalian development^1,115^, lineage dynamics were strikingly consistent across timepoints and embryos comprising clonal populations with unique founding cells. Fate restriction trajectories, cell type ancestral linkages, progenitor clade numbers, clonal outputs, and commitment timing also showed minimal variation across replicate embryos, with pairwise linkage estimates across developing cell types differing by less than six hours. These results reveal that, despite its regulative flexibility, mammalian development is governed by tightly constrained programs that produce robust fate outcomes.

Second, germ layer restriction begins before gastrulation and extends beyond it. Detectable bias in epiblast fate distributions is evident as early as ~E4.0 based on branch length estimates–around the time of implantation but well before the onset of gastrulation–indicating that spatial organization and signaling in the post-implantation epiblast already begin to bias progenitor outputs at this early stage. These early spatial biases leave a lasting imprint on lineage architecture: A-P and D-V relationships established in the epiblast remain detectable days later, with A-P identity in particular showing strong heritability. By contrast, late-arising fate restriction governs the elongation of the tailbud, where highly proliferative, bipotent axial progenitors continue to generate additional mesodermal and ectodermal progenitors through E9.5.

Third, cell types can converge on shared transcriptional programs from distinct lineage origins. Endothelial cells arise from multiple mesodermal sources–including lateral plate, intermediate, and paraxial mesoderm–yet converge on a shared core transcriptional program while retaining detectable signatures of their tissue of origin. A similar pattern is observed in the heart, where atrial and ventricular cardiomyocytes share common transcriptional programs, yet descend from essentially non-overlapping lineage compartments from the first, anterior second, and posterior second heart fields. These examples illustrate how cell state convergence can mask underlying lineage differences, which would be obscured by approaches that infer lineage from transcriptional similarity alone, and underscore the importance of direct lineage measurements for resolving the origins of cell type diversity^23^.

Finally, transcriptional and regional structures in the embryo do not explicitly correspond to lineage boundaries. Spatial context and local signaling can outweigh lineage history in determining fate. For example, at the midbrain-hindbrain boundary, clades frequently span both regions despite sharp transcriptional differences, suggesting that regional boundaries in the brain arise from later molecular specification rather than early lineage compartmentalization. Similarly, trunk neural crest-restricted progenitors retain the capacity to generate both DRG and autonomic neurons at the time of fate restriction, yet their descendants rapidly resolve into distinct fates, likely in response to local cues encountered during migration; however, whether commitment occurs before or after delamination cannot be definitively resolved from our data^64,66^. Together with the preceding findings, these observations highlight how the varied influences of cell state, lineage, signaling, and positional cues shape cell fate restriction across tissues.

This work provides a foundation for future studies that integrate lineage, transcriptional, and spatial information at whole-embryo scale. PEtracer is directly compatible with imaging-based spatial profiling approaches such as MERFISH^42,45^, enabling work to map lineage relationships onto tissue architecture, revealing how local signaling environments, morphogen gradients, and cell-cell interactions shape fate decisions *in vivo*. The unbiased and embryonic-scale dissociative approach taken here was a deliberate first step, providing the cell type annotations, marker genes, developmental staging, and lineage scaffolds needed to design and interpret future spatially resolved experiments. Extension to later timepoints and application of genetic or environmental perturbations with our chimeric embryo strategy will further expand this framework across the full arc of organogenesis and enable direct dissection of the molecular mechanisms governing cell fate determination. Beyond embryogenesis, these approaches can be used to dissect progenitor hierarchies and clonal dynamics in adult tissue homeostasis, regeneration, and disease–contexts in which developmental fate decisions are reactivated, repurposed, or subverted^112,116,117^.

Beyond these experimental extensions, the time-resolved, near-complete lineage maps generated here provide an unprecedented training set for AI models of embryogenesis. A central challenge in learning developmental dynamics from discrete snapshots is inferring continuous trajectories between timepoints, a problem that lineage information directly constrains by encoding ancestral relationships between cells^118,23^. We aim to leverage our joint lineage and transcriptional measurements across timepoints to build models that learn the probabilistic rules governing cell fate transitions, toward the ultimate goal of a virtual model of embryogenesis that can be interrogated, perturbed, and used to predict developmental outcomes *in silico*^119^.

Our data establish a common lineage coordinate system that naturally complements focused mechanistic studies such as prospective labeling, genetic and environmental perturbations, and *in vitro* differentiation. The near-comprehensive resolution of cell fate dynamics captured here also offers a roadmap for producing physiologically relevant cell types, and can help unify gene regulatory programs with the signals that initiate and sustain cell fate decisions. Critically, these data create an opportunity to move from a descriptive to a predictive understanding of development, enabling models that infer the rules governing cell fate transitions and forecast developmental outcomes across genetic and environmental contexts. Extending these experimental and computational platforms to later developmental stages, postnatal tissues, and perturbation-coupled analyses will further enable dissection of how cell state and lineage dynamics govern tissue assembly, maintenance, regeneration, and their disruption in disease. Overall, this work represents an important step toward a lineage-resolved, embryo-scale reference for cell fate specification across mouse development.

### Limitations of the study

Reconstructed lineage trees are not a perfect record of developmental history. Unmarked divisions and incomplete cellular sampling mean that some progenitor-progeny relationships are missed in reconstructed trees. Branch lengths rely on a molecular clock assumption in which edit accumulation is approximately proportional to elapsed time; although PEmax expression and lineage mark installation rates were consistent across cell types, individual time estimates should be interpreted as approximate. Importantly, the trees reconstructed here capture the natural developmental fates of cells in their native context, not their developmental potential–cells observed to adopt a particular fate *in vivo* may retain the capacity to generate other cell types if placed in an ectopic environment or removed from their normal signaling context^120^. These constraints highlight the importance of orthogonal experimental approaches as we explore the hypotheses generated with these data.

Further, cell type annotations were assigned manually based on marker gene expression and integration with existing atlases, and are therefore limited by the resolution of current references. Rare or transitional states may be under-resolved, and boundaries between closely related cell types may not reflect discrete biological categories. Similarly, spatial coordinates along the A-P and D-V axes were imputed from spatially predictive genes rather than measured directly, and may be less accurate in regions where spatial gene expression gradients are weaker.

The embryos profiled here are chimeras seeded by 2–6 donor mESCs rather than derived from a single zygote, which may introduce slight differences in developmental timing and progenitor allocation relative to wild-type embryos. While host-derived wild-type cells in each embryo serve as an internal control for developmental competence, and overall cell type distributions closely match established references, the earliest fate decisions should be interpreted in this engineered context. Additionally, while we observe no overt effects of lineage tracing on embryonic development and PEtracer chimeric embryos can develop normally to term, subtle effects of PEtracer components on cell state cannot be fully excluded.

## Materials and methods

### Cloning of the Rosa26 PEmax-T2A-GFP knock-in cassette

Gibson assembly was used to assemble DNA fragments encoding PEmax-T2A-GFP into a digested backbone designed for knock-in of genetic cargos into the mouse genomic Rosa26 locus (Addgene plasmid #61408)^121^. Briefly, backbone plasmid was digested with MluI (NEB) and BstBI (NEB) according to manufacturer’s recommendations. PEmax-T2A-GFP was amplified using Q5 High-Fidelity 2X Master Mix (NEB). Fragments were assembled into complete plasmids using 5 μL of NEBuilder 2X HiFi DNA Assembly master mix (NEB) combined with 0.05pmol backbone and 0.15pmol of each insert fragment brought to a final volume of 10 μL with ultrapure nuclease-free water. Assemblies were incubated for 60 minutes at 50°C prior to transformation into NEB stable competent *E. coli* (C3040, NEB). Transformation included a 15-minute incubation of 50 μL of competent cells and 5 μL of assembly mix prior to a 30-second heat shock at 42°C and a subsequent 5-minute incubation on ice before plating on 100 μg/mL carbenicillin-containing agar plates.

### Cloning of upstream U6-containing 24-mer pegArrays

24-mer pegArrays were cloned as previously described^42^, substituting a novel backbone that includes two U6 promoters that do not express any gRNA upstream of the 24-mer array. This strategy was used to minimize positional effects that decrease the expression levels of the first two pegRNAs in our arrays. Briefly, 24-mer assemblies require four pieces of DNA that include a PaqCI digested 24-mer backbone and a PaqCI digested fragment for each edit site 8-mer prepared as described previously. PaqCI digestions were performed overnight using 4 μL of CutSmart buffer, 4 μL of PaqCI activator, 2 μL of PaqCI, and 30 μL of the plasmid of interest. These digested fragments were gel extracted using the QIAquick Gel Extraction Kit (Qiagen) following manufacturer’s recommendations. Digested fragments were assembled into 30 μL PaqCI golden gate assemblies with 3.33 nM of the PaqCI digested 8-mer fragments (typically 1 μL of the ~4,000nt fragment diluted to 150 ng/μL), 1 μL of PaqCI pre-digested 24-mer acceptor backbone diluted to 25 ng/μL, 2.5 μL PaqCI enzyme, 1.25 μL PaqCI activator, 10 μL NEBridge Ligase Master Mix (M1100L), and water to bring the reaction to 30 μL total volume. Assemblies were carried out following manufacturer’s recommendations, carrying out 99 cycles of 5 minutes at 37°C and 5 minutes at 16°C before a 10-minute 60°C incubation and subsequent 4°C hold. These reactions were transformed into NEB Stable Competent *E. coli* (NEB, C3040), incubating on ice for 20 minutes prior to a 30 second heat shock at 42°C. After heat shock, bacteria were incubated on ice for 5 minutes prior to plating on 100 μg/mL carbenicillin-containing agar plates. Plates were incubated overnight at 30°C prior to picking colonies into 5 mL of 100 μg/mL carbenicillin-containing terrific broth, outgrowth overnight at 30°C, and DNA isolation using the Promega PureYield DNA Miniprep System. Some bacteria were expanded into 100 mL cultures overnight while isolated plasmids were diluted to 100 ng/μL and submitted for whole plasmid sequencing. DNA was isolated from these larger cultures using the ZymoPure II Plasmid Midiprep Kit. Plasmids were sequence confirmed by whole plasmid sequencing and further validated to have the correct insert by gel electrophoresis to be the correct size using restriction enzyme digestion.

### Cloning of a library of non-silencing PEtracer lineage tracing cassettes

We cloned our previous library of PEtracer lineage tracing cassettes (LTCs) into a backbone compatible with PiggyBac transposition, which leveraged recent developments in non-silencing promoter elements derived from Addgene plasmid #239046^57^. The cloned parent backbone was digested with PacI and the LTC library was introduced via Gibson assembly as previously described^42^. Libraries were transformed into MegaX electrocompetent cells as previously described, and DNA was isolated using the ZymoPure II plasmid midiprep kit (Zymo Research). The resulting plasmid library was sequence confirmed by whole plasmid sequencing (Plasmidsaurus, Quintara) prior to introduction into mouse embryonic stem cells (mESCs).

### Plasmid availability

The cloned plasmids and LTC library listed above will be available through Addgene:

- Rosa26 PEmax-T2A-GFP knock-in cassette
- Upstream U6 24-mer acceptor backbone
- Non-silencing mESC piggybac LTC library

### General Mouse Embryonic Stem Cell Culture Conditions

Mouse embryonic stem cells (mESCs) were maintained on mitotically inactivated DR4 mouse embryonic fibroblast (MEF) feeders in mESC medium, as previously described^122^. Briefly, cells were cultured in DMEM (Gibco) supplemented with 10% fetal bovine serum (HyClone), 1,000 units/mL leukemia inhibitory factor (ESGRO; Sigma), 2 mM L-glutamine (MP Biomedicals), nonessential amino acids (Gibco), penicillin-streptomycin (VWR), and β-mercaptoethanol. Cultures were maintained at 37°C in 5% CO_2_.

MEFs were mitotically inactivated by gamma irradiation and cryopreserved prior to use. For ESC culture, dishes were coated with 2% gelatin at room temperature for ≥30 min. MEFs were plated 24–48 hours before ESC seeding at the following densities (cells/well): 6-well: 5 × 10^3^; 12-well: 2.5 × 10^3^; 48-well: 6.25 × 10^3^; and 10-cm dish: 3 × 10^6.^

Dissociation of ESCs was carried out every 2–3 days by using 0.25% trypsin (LIFE technologies) following a wash with HEPES-buffered medium (in-house HEPES buffer was prepared containing 18.4 mM HEPES, 133.5 mM NaCl, 5.2 mM KCl, 5.3 mM dextrose, 0.42 mM KH_2_PO_4_, and 0.29 mM Na_2_HPO_4_·7H_2_O, phenol red, pH 7.25–7.35, 285–295 mOsm/kg) and incubation at 37°C for 5–10 min. Trypsinization was quenched with serum-containing medium (2x or more the volume of trypsin), and cells were triturated to a single-cell suspension before being pelleted by centrifugation (200 RCF, 5 min).

For cryopreservation, an equal volume of 2x ESC freezing medium (50% FBS, 30% mESC medium, 20% DMSO) was added to the cell suspension. Cells were transferred to −80°C in a slow-cool chamber (Nalgene), for at least 24 hours before storage in liquid nitrogen.

### Generation of PEmax-T2A-GFP mESCs

PEmax-T2A-GFP mESCs were generated by homology-directed repair using two plasmids: (i) a Rosa26-PEmax-T2A-GFP knock-in donor plasmid (described above) and (ii) a plasmid encoding S. pyogenes Cas9 and a Rosa26-targeting sgRNA to induce a double-strand break at the Rosa26 locus. The Cas9/sgRNA plasmid was derived from Addgene plasmid #62988, into which the Rosa26 sgRNA sequence (GACTCCAGTCTTTCTAGAAGA) was cloned using BbsI-HF restriction sites. Low passage (p17) V6.5 (129SvJae × C57BL/6)^123^ ESCs were thawed three days prior to nucleofection. On the day of the nucleofection, cells at 60–70% confluency were dissociated using trypsin (see above) and pre-plated on 2% gelatin-coated dishes for 30 min to deplete MEFs. Non-adherent ESCs were collected, counted, and 1 × 10^6^ cells were pelleted for nucleofection. For nucleofection, cell pellets were resuspended in 100 μL of P3 Nucleofector solution (Lonza, V4XP-3024) and mixed with 0.5 μg of the Cas9/sgRNA plasmid and 1.5 μg of the Rosa26-PEmax-T2A-GFP donor plasmid. Nucleofection was performed using a 4D-Nucleofector X Unit (Lonza) with program CG-104. Immediately after nucleofection, cells were plated at clonal density (1,000–5,000 cells per 10-cm dish) onto DR4 MEF feeders. Selection with G418 (300 μg/mL) was initiated 48 hours post-nucleofection and maintained for 6 days. Individual colonies (n = 48) were manually picked and expanded in 96-well plates.

### Genotyping of PEmax-T2A-GFP mESCs

Colonies were isolated following HDR engineering and selection using neomycin at 300 μg/ml. Individual colonies were expanded and genomic DNA was extracted using QuickExtract DNA Extraction Solution (Biosearch Technologies, QE09050) following manufacturer’s protocols; specifically, a 15-minute incubation at 65°C followed by a 5-minute incubation at 95°C followed by a hold at 12°C. Genotyping was carried out using the Phusion Green Hot Start II High-Fidelity PCR 2x Master Mix with the following cycle conditions: 98°C for 2 minutes, [98°C for 10 seconds, 65°C for 20 seconds, 72°C for 60 seconds]x34, 72°C for 2 minutes. Multiple primer pairs were used to check the integrity of multiple portions of the knocked-in sequence.

1. To check the presence of the PEmax insert: 363 bp amplicon with CTCTGTGGGCTGGGCC + ATGGTGGGGTACTTCTCGTG
2. To check the integrity of the left homology arm sequence: 283 bp amplicon with AAAGTCGCTCTGAGTTGTTAT + GGCTATGAACTAATGACCCCG
3. To check the status of a Rosa26 genomic knock-in: 607 bp if there is no knock-in or a heterozygous knock-in; no amplification if homozygous knock-in with AAAGTCGCTCTGAGTTGTTAT + GGAGCGGGAGAAATGGATATG
4. To check the status of the recombination cassette: 1550 bp if LSL is intact, 882 bp if LSL is recombined with GGGCAACGTGCTGGTTATTG + ATGGTGGGGTACTTCTCGTG
5. An additional primer pair to check the status of the recombination cassette: 1271 bp if not recombined, no amplification if recombined with CCTCCCCCTGAACCTGAAAC + ATGGTGGGGTACTTCTCGTG

### Mice

All procedures were performed in specialized specific-pathogen-free facilities, following all relevant animal welfare guidelines and regulations, with mice kept on a 12 h light-dark cycle from 6 am to 6 pm, and provided a standard diet ad libitum. All mouse experiments described in this study were approved by the Massachusetts Institute of Technology Institutional Animal Care and Use Committee (IACUC, protocol numbers 2311000598 and 2208000407), the Yale University Committee on the Use of Animals in Research and Teaching (IACUC, Protocol #2026–20357) or by the LaGeSo Berlin (Protocol #G0098/23).

### Creation of a homozygous PEmax-T2A-GFP mouse strain

mESC clones G3 and F3, confirmed to be heterozygous for PEmax expression, were used for blastocyst injection as previously described^13^. Briefly, ESC clones were thawed and cultured for 3 days prior to dissociation for microinjection. Donor embryos were obtained from B6D2F1[BDF1] females (C57BL/6J x DBA2, Charles River) following superovulation induced by pregnant mare serum (7.5 IU PMS, ProSpecBio), followed 48 hours later by human chorionic gonadotropin (7.5 IU hCG, ProSpecBio), and subsequent overnight mating with BDF1 males. Eight-cell stage embryos were collected at embryonic day 2.5 (E2.5) and cultured overnight in EmbryoMax^®^ KSOM Advanced Embryo Medium (MilliporeSigma) under oil to the blastocyst stage. For injections, 4–8 ESCs were introduced into the blastocoel cavity of each embryo. A total of 20 injected blastocysts were transferred into the uterine horn of each pseudopregnant CD1 recipient female at day 2.5 post coitum.

### Isolation of homozygous PEmax-T2A-GFP mESCs

Morula-stage embryos were isolated from PEmax-T2A-GFP homozygous mice that had been superovulated (5 IU PMS followed by 5 IU hCG 48h later) and paired with homozygous males. Embryos were harvested 2 days post coitum and cultured overnight to the blastocyst stage in EmbryoMax^®^ KSOM Advanced Embryo Medium (MilliporeSigma) under oil. ESC derivation was performed as previously described^124^. Briefly, blastocysts were exposed to Acid Tyrode’s solution (MilliporeSigma) for 30–60 seconds to remove the zona pellucida, followed by two to three washes in serum-containing mESC medium, from now on referred to as mESC medium. Individual embryos were then transferred into wells of a 96-well plate pre-seeded with MEFs. Embryos were cultured undisturbed for 5 days in serum-free medium supplemented with 15% KnockOut Serum Replacement (KSR) and 5 μM of the Mek1 inhibitor PD98059 (Cell Signaling Technology) from now on referred to as mouse ESC derivation medium (mESCD medium). On day 5, outgrowths were identified and dissociated following a single wash with HEPES-buffered medium, incubation with trypsin (5 min), and quenching with mESC medium. The entire cell suspension was transferred to a 48-well plate containing derivation medium and cultured until ESC colonies emerged. Three to four days later, ESCs were passaged onto one or two wells of a 12-well plate, depending on cell density, and subsequently maintained in mESC medium. Established cell lines were cryopreserved for later use and injection into blastocysts to generate chimeras. Clone sex was determined by PCR (primer sequences and conditions provided below), and male clones were selected for karyotyping.

### Karyotyping analysis

Cells/clones were thawed into T25 flasks covered with DR4 MEFs and cultured for 4–5 days in mESC Medium in order to prepare for submission. Cell lines were submitted to Cell Line Genetics according to supplier’s protocols for karyotyping analyses.

### Establishing PEmax-T2A-GFP mESCs with high numbers of integrated LTCs

PEmax-T2A-GFP mESCs were thawed 3 days prior to nucleofection. After dissociation, 2 × 10^6^ cells were resuspended in 100 ul P3 Nucleofector solution (Lonza, V4XP-3024) containing 5 μg of donor plasmid and 1 μg of Super PiggyBac transposase expression vector. Nucleofection was carried out using a 4D-Nucleofector X Unit (Lonza; program CG-104) and all cells were immediately plated in a single 6-well covered with MEFs. Cells were passaged 1:1 to a 10cm plate 2 days after. After nucleofection, cells were cultured for 7 days with passaging every 2–3 days to allow recovery and expansion. The top 5% of mCherry^+^ cells were isolated by fluorescence-activated cell sorting (FACS; BD FACSAria II, BD Biosciences). Sorted cells (2,000 per 10-cm dish; three dishes per clone) were plated onto MEF feeders and cultured for 5–7 days to allow colony formation. Individual colonies were manually picked into 96-well plates containing MEFs. The following day, cells were passaged onto fresh 96-well plates seeded with half-density MEFs and cultured for 2 days, followed by a 1:3 split and further expansion for 3 days. Three replicate plates were cryopreserved, two frozen for future expansion and genomic DNA was isolated from the third.

### Screening for Sorted PEmax-T2A-GFP mESCs with high numbers of integrated LTCs

Genomic DNA was isolated from mESC clones using QuickExtract DNA isolation solution according to manufacturer’s protocols (Millipore Sigma). The number of integrated LTCs per clone was determined following amplification and bulk sequencing as previously described^42^. Briefly, two successive rounds of PCR were used to amplify lineage tracing cassettes with 2 μL of gDNA and 500 nM primer mix using the Phusion Green Hot Start II High-Fidelity PCR 2x Master Mix with the following cycle conditions: 98°C for 2 minutes, [98°C for 10 seconds, 65°C for 20 seconds, 72°C for 30 seconds]x28, 72°C for 2 minutes. PCR1 was conducted using a pool of forward and reverse primers where each primer was pooled in an equimolar fashion to enable sequencing on an Illumina sequencer with minimal need for added PhiX. All PCR1 amplicons were all checked on 2% agarose (VWR) gels with 1 μL of ethidium bromide (Thermo Fisher Scientific) per 10 mL of 1x Tris-Acetate-EDTA (TAE) buffer. PCR2 amplification was carried out using Nextera adapter PCR primers (listed in Table S4) with the following conditions: 98°C for 2 minutes, [98°C for 10 seconds, 61°C for 20 seconds, 72°C for 30 seconds]x8, 72°C for 2 minutes. 5 μL of PCR1 products were used for each PCR2 reaction and 500 nM final primer concentrations were used. Following PCR2, all reactions for a given genomic locus were pooled together, run on a 2% agarose gel, and gel extracted using the QIAquick gel extraction kit (Qiagen). Concentrations of purified libraries were determined using a Qubit double-stranded DNA high sensitivity Kit (Thermo Fisher Scientific) according to the manufacturer’s instructions. Libraries were diluted to appropriate concentrations for sequencing on an Illumina MiSeq, NextSeq, or NovaSeq.

**LTC Forward primer pool:** TCGTCGGCAGCGTCAGATGTGTATAAGAGACAG-[1N to 4N]- GAATCCAGCTAGCTGTGCAGC
**LTC Reverse primer pool:** GTCTCGTGGGCTCGGAGATGTGTATAAGAGACAG-[1N to 4N]- CCTTAGCCGCTAATAGGTGAGC

Clones with desired integration levels were thawed and subsequently expanded (48-well to 12-well to 6-well format), cryopreserved, and subjected to karyotype analysis.

### Generation of tracing-competent PEtracer mESCs

PEmax-T2A-GFP homozygous mESCs harboring 35 unique LTCs using PiggyBac transposition were karyotyped prior to introducing slow, intermediate, and fast kinetics 24-mer pegArrays. Electroporations were conducted as above, where 2 × 10^6^ cells were nucleofected with 5 μg of transfer plasmid and 1 μg of Super PiggyBac Transposase Expression Vector (System Biosciences, PB210PA-1) using the CG104 protocol on the Lonza 4D-electroporation system. Cells were allowed to recover and expand over two weeks prior to FACS enrichment for the top 10% BFP-expressing cells. Individual clones were subsequently isolated and karyotyped prior to further use. Further, clones were evaluated by bulk sequencing for the presence and relative abundance of each pegRNA in the 24mer array. These PCRs were conducted as described above using two successive rounds of PCR with 2 μL of gDNA and 500 nM primer mix using the Phusion Green Hot Start II High-Fidelity PCR 2x Master Mix with the following cycle conditions: 98°C for 2 minutes, [98°C for 10 seconds, 70°C for 20 seconds, 72°C for 45 seconds]x29, 72°C for 2 minutes. PCR1 was conducted using a pool of forward and reverse primers where each primer was pooled in an equimolar fashion. All subsequent steps were carried out as described above in “Screening for Sorted PEmax-T2A-GFP mESCs with high numbers of integrated LTCs“.

**pegArray Forward primer pool:** TCGTCGGCAGCGTCAGATGTGTATAAGAGACAG -[1N-to-4N]-GAAAAAGTGGCACCGAGTCGGTG
**pegArray Reverse primer pool:** GTCTCGTGGGCTCGGAGATGTGTATAAGAGACAG- [1N-to-4N]-NGTTGGTTTAACGCGTAACTAGATAGAACCG

### Long-read whole genome sequencing of homozygous PEmax-T2A-GFP, high-copy LTC mESCs

Following karyotype analysis, we sought to further characterize our lead line with 36 integrated LTCs using long-read whole genome sequencing. This line was thawed, expanded, and >10^6^ mCherry^+^ cells were isolated by FACS prior to genomic DNA isolation using the PacBio NanoBind CCB kit (102-301-900). Genomic DNA libraries for Oxford Nanopore sequencing were prepared using the Ligation Sequencing Kit V14 (SQK-LSK114; Oxford Nanopore Technologies) according to the manufacturer’s protocol. Briefly, high-molecular-weight genomic DNA was first assessed for quality and quantity using NanoDrop, and samples were normalized to the recommended input mass (2ug). DNA underwent end-repair and dA-tailing. Following a bead-based cleanup step to remove enzymes and short fragments, sequencing adapters supplied in the kit were ligated to the prepared DNA. Adapter-ligated libraries were purified using magnetic beads, and the final library was eluted in an elution buffer provided in the kit. Prepared libraries were loaded onto a PromethION flow cell with R10 nanopores and sequenced on the Oxford Nanopore P2 Solo platform. Sequencing runs were conducted using default parameters in MinKNOW software, with real-time basecalling enabled to generate FASTQ files for downstream analysis.

### Mapping LTCs using long-read whole genome sequencing

FASTQ files were aligned to the mouse reference genome (GRCm39) using a long-read aligner, and resulting alignments were processed to identify LTC integration sites. We leveraged the characteristic size of the LTC (~7 kb) to detect insertions directly from individual long-read alignments. Specifically, we scanned CIGAR strings to identify large insertion events (6.9–7.3 kb) that were flanked on both sides by aligned sequence, ensuring confident anchoring of the insertion to the reference genome. For each such event, we recorded the genomic insertion position, extracted the inserted sequence from the read, and retained the longest qualifying insertion per read. Extracted insertion sequences were oriented to a consistent strand and parsed to recover the integration barcode sequence by identifying sequence motifs flanking the barcode region. The inferred sequences were then error-corrected by matching to a predefined intBC whitelist. This approach enabled direct linkage of individual LTC integrations to genomic coordinates. Final insertion calls were manually inspected using IGV to confirm alignment quality and validate integration sites.

### *In vitro* activation and collection for bulk sequencing

PEtracer mESCs were nucleofected and FACS-sorted for mCherry and GFP double-positive populations as described below (see “Generation of PEtracer mESC-derived chimeric embryos“). Following sorting, cells were plated back to culture instead of being used for aggregation. For time-course collection, independent nucleofections were performed for each biological replicate (n = 3). From each nucleofection batch, cells were cultured in parallel, with routine passaging every 2 to 3 days, and harvested at days 0, 2, 4, 6, and 8 post-sorting. At each time point, MEF-depleted cell pellets were collected for bulk sequencing. Genomic DNA was extracted from flash-frozen cell pellets using the DNeasy Blood and Tissue Miniprep kit (Qiagen, 69504), and LTCs were amplified and subjected to bulk sequencing as described above (see “Screening for Sorted PEmax-T2A-GFP mESCs with high numbers of integrated LTCs“).

### Generation of PEtracer mESC-derived chimeric embryos

PEtracer mESCs (1–2 million cells) were nucleofected with 10 μg pCAG-Cre plasmid (Addgene plasmid #13775) using the P3 Primary Cell 4D-Nucleofector^®^ X Kit L (Lonza, V4XP-3024) and the CG-104 program on a Lonza 4D-Nucleofector X system, following the manufacturer’s instructions and as previously described^125,126^. At 24 hours post-nucleofection, cells were trypsinized and sorted for mCherry and GFP double-positive populations using a BD FACSAria^™^ II Cell Sorter (BD Biosciences) operated with BD FACSDiva software (v8.0.1). Cells were first gated to exclude MEFs, debris, and doublets based on forward and side scatters. Fluorescence was detected using a 510/20 filter for GFP and a 615/20 filter for mCherry. GFP-positive cells were defined relative to non-transfected control cells to establish background fluorescence, and the top approximately 80% of GFP-expressing cells within the mCherry^+^/GFP^+^ double-positive population were sorted and maintained on ice until aggregation. Subsequently, 6–8 healthy PEtracer mESCs were injected into precompaction 4- to 8-cell stage embryos derived from natural matings of CD1 mice for high-efficiency incorporation into the inner cell mass ~24 hours later^127–129^. For tetraploidization experiments, 2-cell stage embryos were tetraploidized by electrofusion as described previously using the CF150/B Cell Fusion instrument (BLS; Poueymirou et al., 2007). Injections were performed using ES blastocyst injection pipettes (BioMedical Instruments; VESsp-15-25-1–55; inner diameter 15 μm, bent angle 25°, bent length 1 mm, pipette length 55 mm; glass: BM100T-10P), in conjunction with an H.T. Laser 6 system (software version 6.5.1) and a CellTram injection system. Injected embryos were cultured overnight in EmbryoMax^®^ Advanced KSOM Embryo Medium (Sigma-Aldrich, MR-101-D), overlaid in pretested embryo-grade mineral oil (Sigma-Aldrich M5310), and transferred into the uteri of pseudopregnant CD-1 strain females around 18 hours post-injection. Embryos were subsequently collected at the indicated developmental stages for downstream chimerism scoring and analyses.

### Bulk sequencing analysis of chimeric E9.5 mouse embryos

Genomic DNA was isolated from intact chimeric E9.5 embryos using the DNeasy Blood and Tissue Miniprep kit (Qiagen, 69504). LTCs were amplified and sequenced as described above (see “Screening for Sorted PEmax-T2A-GFP mESCs with high numbers of integrated LTCs“). Edit saturation and LM distributions were calculated as previously described^42^.

### Imaging-based documentation of experimental embryos

All whole embryo images captured prior to scRNA-seq were performed using a Zeiss Observer 7 with a 5x or 10x air objective.

### scRNA-seq of experimental embryos

Whole embryos (E7.5-E10.0, 12 hr increments) were isolated and cleaned of maternal and extraembryonic tissue in a petri dish with 1x DPBS, then serially washed through several drops to remove maternal tissue. Complete embryos were initially screened by fluorescence imaging to identify high-grade chimeras, followed by documentation at 10x magnification. Following this initial selection, each embryo was individually transferred into a 200–500 μl drop of 0.2% BSA/PBS and dissociated into single cells by replacing the 0.2% BSA/PBS with an equal volume of TrypLE (Invitrogen) and incubated for 10 minutes at 37°C. After the incubation, embryos were mechanically dissociated using a P200 pipette and incubated >2x at 5 minute intervals. The cell suspension was quenched by serially adding and collecting 200 μl of 0.2% BSA/PBS and carefully resuspending cells collected into a LoBind Eppendorf tube until the final volume was ~1 mL (Eppendorf). A 1x Red Blood Cell lysis solution (Miltenyi Biotec) was used to treat cells from E8.5-E10.0 embryos. Cells were filtered into a new LoBind Eppendorf tube using a Scienceware Flowmi cell strainer, 40–70 μm (VWR) and centrifuged for 5 min at 1200 rpm at 4°C. Following centrifugation, ~250 μl of supernatant was removed and cells were resuspended. This process was repeated to concentrate the cell pellet. Cells were resuspended in 0.2% BSA/PBS.

Approximately 5% of the cell suspension was then used to determine the overall percent chimerism of candidate embryos by flow analysis using a BD Celesta according to pre-defined gating parameters. Embryos with >70% chimerism were chosen as high-quality candidates for full embryo sequencing, followed by an estimation of the total cell count using a hemocytometer. For each embryo, the entire cell suspension was processed for single-cell RNA sequencing (10x Genomics, Chromium GEM-X Single Cell 3' Chip Kit v4), distributing cells across multiple reactions to remain within the maximum recommended input of cells per reaction. During optimization, multiple PEtracer lines with distinct editing rates were tested and sequenced with recovery targets of ~10–20,000 cells using the 10x Chromium NextGEM v3.1 3’UTR capturing technology (10x Genomics, Chromium Single Cell NexGEM v3.1). Single-cell libraries were prepared following the manufacturer’s protocol with cDNA (Step 2) and indexing amplification (Step 3) PCR cycle numbers of 10 each. Primary cDNA and final libraries were examined on a D5000 and D1000 TapeStation System, respectively (Agilent Technologies).

### Target site library preparation

Following Step 2 of the 10x Genomics GEM-X workflow, a portion of amplified cDNA was used to generate target site libraries using pooled equimolar staggered forward primers and a common reverse primer (Table S4). The optimal PCR1 cycle number was first determined by qPCR on a 1:8 cDNA dilution using KAPA HiFi HotStart ReadyMix (Roche, 07958935001) and SYBR Green (Invitrogen, S7563; 95°C 3 min, [98°C 20 sec, 65°C 15 sec, 72°C 15 sec]x40, 72°C 1 min); PCR1 was then performed on undiluted cDNA using the same primer set and cycling conditions for two fewer cycles than the qPCR plateau (typically 22 cycles). A second PCR on a 1:25 dilution of the PCR1 product incorporated Nextera i7 indices and sequencing adapters (Table S4; 95°C 3 min, [98°C 20 sec, 72°C 30 sec]x14, 72°C 1 min). The final libraries were purified by 0.9x SPRI bead cleanup and eluted in Buffer EB. Libraries were quantified by BioAnalyzer (Agilent) to assess the size and purity of final libraries and Qubit double-stranded DNA high sensitivity Kit (Thermo Fisher Scientific) to determine the concentrations. Libraries were diluted to 4 nM and sequenced on a MiSeq, NextSeq 2000, or NovaSeq X Plus where appropriate (Illumina). All PCRs were first monitored to determine optimal cycle number by qPCR by adding 0.6x SYBR Green (Thermo) to the PCR reactions.

### scRNA-seq data processing

All scRNA-seq data were processed using Cell Ranger (v9.0.1). Gene expression and lineage tracing libraries were each aligned to a custom reference genome built by appending 2,171 possible LTC sequences to the mouse GRCm38 assembly, enabling joint recovery of transcriptional and lineage information from the same cells. Gene expression was quantified against GENCODE vM37 gene annotations. Integration barcodes (intBCs) and lineage marks (LMs) were called from LTC-aligned reads using a custom Python script, as described previously^42^.

### scRNA-seq quality control

To generate a high-quality dataset with accurate lineage trees, we applied a multistep quality control workflow to remove low-quality cells, low-confidence LTC reads, ambient RNA-derived signal, and doublets. Because the abundance and quality of both transcriptome and LTC reads varied across captures, we used adaptive thresholding wherever possible. Specifically, when defining data-driven cutoffs, we fit a two-component Gaussian mixture model to the relevant one-dimensional distribution within each capture and removed observations corresponding to the lower peak of the distribution.

#### Removal of low-quality cells

We first filtered low-quality cells using transcriptome-derived QC metrics. For each cell, we calculated total UMI counts, total mitochondrial counts, and the fraction of mitochondrial transcripts. Within each capture, adaptive thresholding was applied to the distribution of mitochondrial counts among cells with detectable mitochondrial signal, and then to the distribution of total UMI counts among cells passing the mitochondrial filter and having less than 2% mitochondrial reads. Cells falling below either threshold were removed. A minimum total UMI cutoff of 5,000 was enforced across all captures, except for the shallowly sequenced preliminary data, where a threshold of 100 UMIs was used.

#### Removal of ambient LTC reads and PCR artifacts

We next filtered LTC alleles to remove low-support calls consistent with PCR artifacts or ambient RNA. For each allele, we calculated reads per UMI and retained only alleles with at least 5 UMIs and at least 4 reads per UMI. Within each cell–integration barcode (intBC) pair, we then removed alleles with weak support relative to the dominant allele. Specifically, we retained alleles only if their reads-per-UMI value was at least 25% of the maximum observed for that intBC in that cell, unless they had at least 10 reads per UMI, and we further required their UMI count to be at least 25% of the maximum UMI count within that same cell–intBC pair.

#### Identification of donor, host, and doublet cells

We then distinguished donor- and host-derived cells based on LTC expression and single-nucleotide polymorphisms (SNPs) detected in transcriptome reads. For each cell, total LTC UMI counts were summed and normalized within each capture by the median value among retained cells. Donor and host seed populations were defined from the upper and lower tails of the LTC UMI distribution, respectively. Host-specific SNPs were identified by comparison between host and donor seed populations and used to quantify host-like signal in each cell. Donor cells were then called using adaptive thresholding on the ratio of normalized LTC counts to normalized transcriptome counts. Cells classified as donor but showing excess host SNP signal were labeled as donor–host doublets. In captures where the host-specific SNP signal was weak and host cells were female, Xist expression was used as an orthogonal marker to identify donor–host doublets and validate our general thresholds.

We next used lineage information to identify donor–donor doublets based on conflicting LTC alleles. For each allele, we calculated the fraction of UMIs it contributed within its cell-intBC pair and used this information to select a dominant allele. We then quantified allele conflict rates as the fraction of reads assigned to non-dominant alleles. Baseline conflict rates were estimated across low-conflict cells and intBCs, and per-cell conflict fractions were recalculated using only integrations with low background conflict. Cells with allele conflict fractions greater than 3% were classified as donor–donor doublets.

Finally, we identified host-host doublets using an approach based on total UMI counts, as these cannot be resolved using SNPs or lineage information. Donor cells previously classified as singlets or doublets were ordered by normalized transcriptome counts, and the local doublet frequency was estimated using a rolling window. The normalized count threshold at which the local donor doublet frequency exceeded 50% was then used to define a cutoff for host doublets. Host cells above this threshold were labeled as host–host doublets. These and all previously identified doublets were removed from the dataset.

### Lineage tree reconstruction

Lineage trees were reconstructed independently for each embryo using the set of donor cells with >75% intBC detection (95% of cells passed this threshold). For each cell, LTC alleles were converted into a character matrix in which rows corresponded to cells and columns corresponded to editable sites across intBCs. A scalable hybrid approach was used to construct the >100,000-cell lineage trees presented in this study, combining a greedy algorithm that resolves high-confidence splits (supported by ≥3 edits) at the top of the trees with a heuristic neighbor-joining algorithm to reconstruct subtrees within these clades.

To identify high-confidence splits, we applied a greedy recursive partitioning procedure based on shared mutations. Within each set of cells, candidate mutations were enumerated and ranked by prevalence. A seed mutation was selected, and additional mutations with highly overlapping cell sets (Jaccard similarity >0.9) were grouped to define a provisional split. This mutation set was refined by identifying mutations that were highly prevalent within the resulting subset of cells (present in >90% of cells, treating missing data as compatible), and cells matching this refined mutation set were assigned to a child clade. This procedure was applied iteratively, with each split required to be supported by ≥3 shared edits and to contain ≥1% of cells, ensuring that only well-supported divisions were resolved. Recursive splitting was limited to a maximum depth of 3 to prevent over-partitioning and to ensure that downstream reconstruction operated on sufficiently large clades. A small number of cells that did not match any high-confidence clade were removed as likely doublets.

Within each high-confidence clade, lineage trees were reconstructed using the heuristic neighbor-joining algorithm implemented in FastTree 2 (v2.2)^130^. This approach is highly scalable because it avoids explicit computation of the full pairwise distance matrix and has been shown to accurately reconstruct cellular lineage trees^131^. To run FastTree, lineage marks were encoded as amino acid states. Maximum-likelihood optimization and support-value estimation were disabled, such that topology was inferred using the underlying heuristic neighbor-joining procedure. A custom edit-distance matrix was supplied to reflect the structure of lineage recording data, in which only unedited-to-edited transitions occur, and missing values were retained during inference. Only characters detected in >50% of cells and with no single lineage mark represented in >95% of cells were used for subtree reconstruction.

Subtrees were rooted using a character-based outgroup strategy. For each clade, we identified the most frequent non-missing edited state across all characters and treated cells carrying this state as a proxy outgroup. For each edge in the unrooted tree, we evaluated the partition it defined by comparing the overlap between this proxy outgroup and the sets of descendant cells on either side of the edge using a Jaccard-like criterion. The tree was rooted at the edge that maximized this overlap, yielding a root placement consistent with the most globally shared mutation pattern.

Clade-level subtrees were subsequently combined to generate embryo-scale lineage trees. Because each embryo contained multiple independently seeded donor clones, the resulting trees naturally comprised multiple top-level branches corresponding to these clones. To enable clone-resolved analyses, embryo-level trees were partitioned into clone-level trees by splitting at the root, with each direct descendant of the root defining an independent clone-specific lineage tree.

### Branch length estimation

For each clone-level tree, ancestral lineage mark (LM) states were reconstructed using the Sankoff algorithm as previously described^42^. Following ancestral state inference, edges without any inferred mutations were collapsed to simplify the tree topology. This step removes branches that are not supported by any edits, merging nodes with identical inferred states. As a result, the trees contain multifurcations, where a single node gives rise to more than two descendants. These multifurcations reflect unresolved branching order due to insufficient mutational information and ensure that only biologically meaningful branches supported by at least one edit are retained.

Branch lengths were estimated using a maximum-likelihood framework implemented in ConvexML^132^, which models mutation accumulation as an independent exponential process across sites. Under this model, edits arise along each branch according to a Poisson-like process, such that the expected number of mutations is proportional to branch length scaled by site-specific mutation rates, providing a molecular clock–like interpretation in which branches with more edits correspond to longer durations. To account for heterogeneity in editing kinetics, we specified relative mutation rates (0.3, 0.4, and 1.2, repeated across sites). A pseudocount of 1 mutation per edge was included to stabilize estimates for branches with sparse signal. Missing and unedited states were explicitly incorporated into the model, and branch lengths were inferred jointly across the entire tree using convex optimization. Finally, branch lengths were rescaled such that the total depth of each tree matched the known sampling time of the embryo, and each internal node was assigned and inferred embryonic day.

### Number of extant cells over time

The number of extant cells over time was calculated by traversing each reconstructed lineage tree and counting the number of branches present at each inferred timepoint. For each edge, the branch was considered extant from the time of the parent node (inclusive) until the time of the child node (exclusive). These counts were then summed across all branches to obtain the number of extant lineages at each timepoint.

### Percent of branches marked by an edit

We quantified the fraction of lineage branches supported by at least one edit by comparing the number of observed branches in the reconstructed trees to the total number of branches expected under a fully bifurcating topology. In the collapsed trees, each observed edge corresponds to a branch supported by one or more inferred edits. However, collapsing mutationless edges introduces multifurcations, in which a node has more than two children. Each multifurcation with k descendants implies the presence of *k-2* unresolved (hidden) branches that are not supported by detectable edits. The total number of branches was therefore defined as the sum of observed branches and these inferred hidden branches, and the overall fraction of branches marked by an edit was calculated as the ratio of observed edges to total branches. These and other tree statistics are reported in Table S1.

To examine how this fraction varies over developmental time, we additionally computed the fraction of marked branches within discrete time intervals. Observed branches were assigned a time corresponding to the midpoint between their parent and child nodes. Hidden branches arising from multifurcations were similarly assigned approximate times based on the parent node and the distribution of child node times, with multiple hidden branches distributed between these values to reflect their implied ordering. Within each time bin, the number of observed (marked) and hidden (unmarked) branches was counted, and the percent of branches marked by an edit was calculated as the fraction of observed branches among all branches in that interval.

### Normalization and highly-variable gene selection

Single-cell analysis was performed using Scanpy (v1.11.5)^133^. Gene expression counts were normalized by scaling total UMIs per cell to 20,000 followed by log1p transformation, and cell cycle scores were computed using curated S phase and G2/M gene sets. To identify highly variable genes while minimizing confounding effects, we excluded genes exhibiting strong bias with respect to donor versus host identity, developmental stage, cell cycle, or sex. Donor- and host-biased genes were identified by differential expression (t-test), and the top 500 genes ranked by absolute effect size were removed. For each developmental stage, the top 20 stage-enriched genes were also excluded. In addition, all cell cycle genes and genes located on the X and Y chromosomes were removed, and genes expressed in fewer than 1,000 cells were filtered out. Highly variable genes were then selected from this filtered gene set using a variance-based approach that accounts for mean expression, selecting the top 5,000 genes while controlling for batch effects across developmental stages; these genes were used for downstream dimensionality reduction and clustering analyses.

### Embedding with scVI and UMAP

Cells were embedded using scVI^134^, which learns a low-dimensional latent representation of gene expression while accounting for technical and biological variability. The model was trained using 50 latent dimensions, 2 hidden layers of 1024 hidden units, 15 epochs, and a batch size of 512, with developmental stage specified as a batch covariate. UMAP embeddings were generated using RAPIDS single-cell^135^ by constructing a 50-nearest neighbor graph from the scVI latent space and applying UMAP with default parameters and a fixed random seed for both global and subcluster visualizations.

### Transcriptional similarity between timepoints and with reference datasets

We measured transcriptional similarity between datasets by estimating distributional distances between cell populations in an integrated scVI latent space. We first co-embedded datasets using the same scVI parameters as above, with the dataset label specified as a batch covariate. We then computed transcriptional similarity using Sinkhorn similarity, defined as a normalized Sinkhorn divergence, where the minimum divergence across all pairs of embryos was scaled to 1 and the maximum divergence was scaled to 0. Sinkhorn divergence was estimated using p=2 and regularization of ε = 0.025, and the mean across 50 minibatches of size 512. We used the Sinkhorn implementation from geomloss^136^.

### Cell type annotation

Cell types were annotated at the level of cell subtypes using a combination of reference-based label transfer, manual curation, and automated approaches. Initial annotations were obtained by transferring labels from published mouse embryo reference atlases, including Pijuan-Sala et al., 2019 and Qiu et al., 2024, based on similarity in gene expression profiles. These labels were refined through manual inspection and cluster-level analysis using cellxgene (v1.3.0), guided by canonical marker genes and literature review. For each cluster, marker genes were identified in two categories: “global” markers distinguishing the cluster from all other cells, and “local” markers distinguishing the cluster from the three most transcriptionally similar cell subtypes. In parallel, automated annotation was performed using Claude Opus 4.5 by providing cluster-level marker genes as input. Final subtype annotations were determined by integrating evidence from label transfer, manual curation, and AI-based predictions. Descriptions and rationale for each annotated subtype, reported in Table S2, were generated using Claude Opus 4.5. Subtypes were subsequently grouped into broader cell types and further annotated with germ layer and developmental domain information.

### Calculating cell fate bias

Cell fate was defined operationally as the distribution of cell states among descendant cells within the reconstructed lineage trees. For each internal node, a fate distribution was computed by tallying the number of descendants belonging to each annotated cell type (or other fate category, such as germ layer). To characterize how these fate distributions evolve over developmental time, we split the tree into discrete time bins (0.5-day intervals for all analyses) and assigned each node’s contribution across these bins based on the temporal span of its lineage. Specifically, each node contributes its fate distribution to all timepoints between the time of its parent (inclusive) and its own time (exclusive). This reflects the biological interpretation that a branch defined by a node persists over the interval from its birth until a division event gives rise to child nodes. Thus, this approach captures how fate bias accumulates along continuous developmental trajectories rather than at discrete branching points.

Fate bias was quantified using a normalized entropy-based score applied to each node’s fate distribution. Specifically, for a node with descendant proportion vector *p*, the fate bias score was defined as *1 - H(p)/H(q)*, where *H(p)* is the entropy of the node’s distribution and *H(q)* is the entropy of the root distribution for the corresponding tree, both of which are estimated via a plug-in estimator. This score ranges from 0 (no bias relative to the root distribution) to 1 (complete restriction to a single fate). To summarize trends over time, a weighted mean fate bias was calculated within each time bin, where each node was weighted in proportion to its number of descendant cells. Statistical significance of fate bias at each timepoint was assessed using permutation testing with 100 permutations: descendant fates were resampled under a null model that preserves total descendant counts per node and the overall root distribution, and weighted mean fate bias was recomputed for each permutation. Observed values were then compared to this null distribution to obtain p-values, providing a measure of when lineage-wide fate bias becomes significantly elevated above what would be expected by random subsampling. The black line in plots with permutation testing indicates the mean across all permutations.

### Identifying fate-restricted clades

Fate-restricted clades were identified from reconstructed lineage trees based on the fate distribution of each node. For each fate category, such as a given cell type or germ layer, we defined a node as fate-restricted if at least 90% of its descendant cells belonged to that fate. To avoid overcounting nested clades, fate-restricted clades were selected using a two-pass procedure applied separately for each fate. First, nodes were evaluated from the leaves upward to identify all nodes whose descendant composition met the 90% threshold. Second, nodes were traversed from the root downward, and the highest qualifying node for each fate was selected; once a node was selected, its descendants were excluded from further consideration for that same fate. This yielded the earliest detectable ancestor of each fate-restricted clade while preventing descendant nodes from being counted repeatedly. The 90% threshold was used to account for minor labeling and tree reconstruction errors – in practice, the vast majority of fate-restricted clades are 100% pure for the given fate.

Multifurcations were handled explicitly because they reflect unresolved branching order rather than true simultaneous multi-way divisions. For nodes with more than two children, if multiple child branches independently qualified for the same fate, the parent node was identified as the fate-restricted ancestor for those children. Biologically, this assumes the most parsimonious scenario—that fate restriction occurred once at an unresolved split, rather than independently in each child branch. This approach avoids artificially inflating the number of fate-restricted clades due to limited lineage resolution, but may miss true cases where multiple independent fate-restriction events occur within a multifurcation. Accordingly, the number of fate-restricted clades should be interpreted as a lower bound on the number of progenitors for a given fate.

### Ancestral linkage analysis

To assess how closely each cell was related to a given target category (e.g., a cell type or germ layer), we computed an ancestral linkage score based on lineage distance within the reconstructed trees^58^. For each cell, we identified the most recent common ancestor (MRCA) shared with the nearest cell belonging to the target category and used the depth of this ancestor as a measure of relatedness, with deeper MRCAs indicating more recent shared ancestry. To account for differences in the abundance and distribution of the target population within the tree, this value was normalized by subtracting a permuted background expectation generated by randomly reassigning non-target labels while preserving the target population. The resulting ancestral linkage score therefore reflects how much more closely related a given cell is to the target population than expected by chance. To summarize these relationships at the population level, per-cell linkage scores were aggregated by grouping cells according to a source category of interest (e.g., cell type) and computing the mean linkage within each group.

To provide a global view of lineage relationships across categories (e.g., cell types or germ layers), this framework was extended by iteratively treating each category as the target and computing mean ancestral linkage from all source categories to that target, yielding a pairwise category-by-category matrix. As these relationships are inherently asymmetric, the resulting matrix was symmetrized by averaging reciprocal source–target values. Statistical significance was assessed using permutation testing (100 permutations), applying the same non-target shuffling scheme described above. To quantify variability, pairwise linkage was computed independently for each embryo, and variation was evaluated across biological replicates from the same developmental timepoint.

### Fate bias motif analysis

To characterize recurrent patterns of early fate bias, we analyzed cell type–level fate distributions at ancestral nodes within reconstructed lineage trees. For each node, descendant cell types were aggregated to generate a count vector, which was then normalized to proportions to define the node’s fate distribution. These distributions were expanded across developmental time by assigning each node to all time bins spanned by its incoming branch, as described above, using half-day time intervals. Analyses were restricted to nodes from E9.5 embryos, focusing on early developmental timepoints (≤ E7.0) and requiring a minimum of 50 descendant cells per node to ensure robust estimation of fate composition.

For each selected node, fate distributions were used as feature vectors to construct a nearest-neighbor graph (k = 20), followed by dimensionality reduction using UMAP and clustering using the Leiden algorithm (resolution = 0.5). Clusters identified in this space represent recurrent “fate bias motifs,” defined as groups of ancestral nodes with similar descendant fate compositions. Motif identities were assigned based on Leiden cluster membership and analyzed to identify common and distinct patterns of early fate bias across embryos.

To quantify the temporal dynamics of fate bias within each motif, we computed a weighted fate bias score across time. For each node, the fate bias score was multiplied by the number of descendant cells to obtain a weighted contribution, reflecting both the strength of bias and the size of the corresponding lineage. These values were then summed across all nodes within each motif and time bin and normalized to the maximum observed value across motifs. This analysis captures how strongly and how extensively each motif contributes to fate bias over developmental time, enabling comparison of the timing and magnitude of bias emergence across distinct fate bias motifs.

### Sibling output analysis

To investigate the fates of sibling lineages, we analyzed the outputs of clades adjacent to fate-restricted nodes within the reconstructed lineage trees. For each fate-restricted clade, the sibling clade was defined as the set of descendant cells arising from the nearest branching point not assigned to that fate. For directly identified clades, sibling clades were defined as the descendants of the parent node excluding the focal clade, whereas for clades identified at unresolved multifurcations, the sibling clade corresponded to the descendants of the same node. To ensure comparable lineage depth and to avoid inclusion of overly broad clades, sibling groups with substantially larger descendant populations than the focal clade were excluded. The composition of sibling clades was then quantified in terms of cell type or germ layer identity and compared to the overall background distribution across the dataset. This analysis provides a measure of how frequently specific fates co-occur within closely related lineages, offering insight into shared developmental origins and spatial or lineage coupling between emerging cell populations.

### Clade co-occurrence

To quantify the extent to which distinct categories (e.g., cell subtypes or heart fields) co-occur within fate-restricted clades, we computed a pairwise co-occurrence statistic. Each clade was treated as a unit, and for each clade we recorded the presence or absence of each category of interest. This yielded a binary clade-by-category matrix indicating whether a given category was represented among the descendants of each clade. Co-occurrence was quantified using pointwise mutual information (PMI), which measures the deviation of observed co-occurrence frequency from that expected under independence. Specifically, for each pair of categories *i* and *j*, PMI was defined as

$$\text{PMI}\left(i,j\right)=\text{log}\frac{P\left(i,j\right)}{P\left(i\right)P\left(j\right)}$$

where *P(i,j)* is the probability that both categories are present within the same clade, and *P(i)* and *P(j)* are their marginal probabilities across clades. Positive PMI values indicate that two categories co-occur more frequently than expected by chance, whereas negative values indicate mutual exclusivity.

### Quantifying within-clade transcriptional similarity

To assess whether heart-restricted clades exhibit additional transcriptional structure beyond annotated cell subtypes, we quantified the similarity of descendant cells within each clade using a low-dimensional embedding. For each clade, pairwise transcriptional similarity between cells was computed from the scVI latent space using cosine similarity, and the mean pairwise similarity across all cell pairs within the clade was used as a summary statistic.

To determine whether observed within-clade similarity exceeded that expected by chance, we performed a stratified permutation test. For each clade, a null distribution was generated by randomly sampling sets of cells of equal size from the most common cell subtype within the clade. Mean pairwise similarity was computed for each permuted set, and the observed value was compared to this null distribution to obtain a p-value and z-score. Clades with significantly higher similarity than expected (FDR-corrected across all clades) were interpreted as exhibiting additional transcriptional substructure, consistent with the presence of lineage-defined sub-domains within cell subtype identities.

### Quantifying neural crest fate restriction dynamics

To characterize fate restriction dynamics within the neural crest (NC), we analyzed trunk and cranial NC-restricted clades and quantified the emergence of differentiated outputs over time. For each NC-restricted clade, descendant cells were annotated by differentiated output (DRG versus autonomic for trunk NC, and trigeminal versus mesenchymal for cranial NC), and clades were binned by inferred restriction time using half-day intervals. Within each time bin, we calculated the probability that a clade contained each differentiated output, as well as the probability of producing multiple outputs (bipotency), defined as the fraction of clades containing both categories. To assess whether co-occurrence of distinct outputs deviated from statistical independence, we compared the observed frequency of bipotent clades to the expected frequency under a model assuming independent occurrence of each output type, calculated as the product of their marginal probabilities.

To further quantify the dynamics of fate resolution within bipotent progenitors, we examined changes in fate bias over time relative to the inferred time of fate restriction. For each bipotent clade, we calculated a fate distribution for differentiated outputs (excluding *Sox10*^+^ progenitors) and used this to compute a normalized entropy-based fate bias score as described above. These distributions were expanded across time bins as described previously, and for each clade, time was recentered relative to the time of fate restriction to account for progressive NC specification along the A-P axis. Mean fate bias trajectories were then computed by averaging across clades within each E9.5 embryo. This analysis captures how initially multipotent neural crest progenitors progressively resolve toward specific lineage outputs following restriction.

### Identifying gene programs with Hotspot

Lineage-associated gene programs were identified using Hotspot (v1.1.3)^87^. Neighbor weights were computed for the five nearest neighbors in the lineage tree using an exponential kernel with σ = 2.0 applied to inferred tree distances. Normalized expression values were used to calculate gene-level local autocorrelation under the ‘normal’ model with 30 parallel jobs. Here, C denotes a normalized, correlation-like measure of the extent to which a gene’s expression covaries among neighboring cells in the lineage tree, with higher values indicating stronger positive local autocorrelation. Genes with local autocorrelation C >0.1 were retained for pairwise analysis.

Pairwise local correlations were then computed among retained genes using Hotspot. Genes were grouped into coherent programs based on their local correlation Z-scores. Coarse clusters were first identified by hierarchical clustering of the Z-score matrix, and gene programs were subsequently defined within each cluster using an agglomerative procedure in which genes were merged when their mean pairwise Z-score was ≥80. Programs containing at least four genes were retained. This approach identified lineage-associated gene programs whose expression covaries among closely related cells in the lineage tree.

Gene program activity was scored in each cell as described in Tirosh et al., 2016. For each program, the score was computed as the average expression of program genes minus the average expression of expression-matched control genes sampled from 25 expression bins. Program scores were used for downstream visualization and lineage-based analyses.

Gene program annotation was performed in a fully automated manner using Claude Opus (v4.6) by providing, for each program, the constituent genes and the set of cell types in which activity was enriched. The model generated a concise name and a brief biological description summarizing the dominant functional theme of each program. These annotations, reported in Table S3, were not manually curated or experimentally validated and should be considered provisional, model-derived summaries intended to aid interpretation rather than definitive biological assignments.

### Inferring ancestral program scores and rates of change

Gene program activity was imputed for internal nodes of the E9.5 lineage trees as the mean score of descendant cells, enabling reconstruction of program dynamics along lineage trajectories. To quantify these dynamics, 100 cells per cell type were randomly sampled, and program scores were computed across half-day intervals from root to leaf, with each internal node assigned a score spanning the interval between its parent and itself, as described above. Mean program activity over time was then obtained by aggregating trajectories within each cell type.

Rates of change in program activity were estimated using a smoothed finite difference of program scores over time. Scores were first smoothed with a centered rolling mean (window = 2 bins), and derivatives were then estimated at each time point as the difference between the subsequent and preceding smoothed values divided by the elapsed time between them (i.e., a centered two-point slope). To generate the rate-of-change heatmap, mean rates were computed across cell types in which a given program reached a maximum score >0.5; if no cell types met this threshold, the highest-scoring cell type was used. For comparability across programs, rate profiles were normalized by their maximum value.

### Estimating transcriptional divergence

To quantify how rapidly transcriptional states diverge among closely related cells, we measured the mean distance in scVI latent space between each cell and its nearest lineage neighbors, defined as cells sharing a common ancestor within a fixed temporal window (maximum lineage distance of 3, corresponding to approximately 1.5 days). For each cell, Euclidean distances to these neighbors were averaged to obtain a transcriptional divergence score, where lower values indicate that closely related cells remain similar and higher values indicate rapid divergence following lineage separation. This metric reflects local dispersion in transcriptional state among related cells rather than directional change. To compare divergence across cell types, per-cell scores were aggregated by taking the median within each annotated cell type.

### Inferring spatial positions using reference spatial-transcriptomics

To infer spatial positions of cells along the anterior-posterior (A-P) and dorsal-ventral (D-V) axes, we leveraged a published spatial transcriptomics dataset from a sagittal section of an E13.5 mouse embryo^97^. This section was selected because it represents a midline plane encompassing the full extent of the axial neural ectoderm. Although the developmental stage does not exactly match that of our dataset, key spatial patterning programs–particularly Hox gene expression–are established early and remain broadly stable across stages.

Within the spatial dataset, brain regions were manually annotated based on anatomical definitions from the Allen Brain Atlas^138,139^. A continuous midline trajectory was defined by tracing the center of the neural ectoderm from anterior to posterior. Each spatial location was projected onto the nearest point along this trajectory to obtain an A-P coordinate, defined as cumulative distance along the path and normalized to the interval [0,1]. D-V coordinates were computed as the signed perpendicular distance from each cell to the midline, with sign determined by the local normal vector; these values were also normalized to [0,1].

To extend these spatial coordinates to single-cell data, we trained regression models to predict A-P and D-V positions from gene expression using the spatial reference. Spatially informative genes were selected based on prior knowledge of developmental patterning systems: A-P prediction relied primarily on Hox and other axial patterning genes, while D–V prediction used canonical dorsal–ventral markers in the spinal cord. For each axis, a random forest regressor was trained with five-fold cross-validation, and performance was evaluated using cross-validated R^2^. The trained models were then applied to the single-cell dataset to infer spatial coordinates for each cell. These inferred positions recapitulated known spatial organization across the developing brain and spinal cord and were used for downstream analyses of spatial patterning and lineage relationships.

### Heritability and bias for spatial positions

To quantify the heritability of spatial identity, we assessed the similarity of inferred spatial positions between closely related clades within reconstructed lineage trees at E9.5. For each internal node corresponding to a neural-restricted clade, we identified its nearest neighboring internal node (also restricted to neural clades) using a graph-based nearest-neighbor approach on the tree. We then compared the mean inferred A–P and D–V positions of the descendant cells for each clade to those of its nearest neighbor. Heritability was quantified as the correlation (R^2^) between these paired values, providing a measure of how strongly spatial identity is preserved across nearby branches in the lineage tree.

To determine when spatial biases first emerge during development, spatial coordinates were inferred for internal nodes by averaging the positions of their descendant cells. These ancestral spatial scores were then expanded across time by assigning each node’s value to all time bins spanning from its parent to itself, as described above. Temporal trends in spatial bias were visualized by grouping nodes according to the spatial position of their descendant cells and computing the mean ancestral position within each time bin. This analysis captures the progressive emergence of spatial organization, revealing when lineage-associated biases along the A–P and D–V axes first become detectable over developmental time. Notably, the most posterior value predicted in our data was ~0.8, consistent with the additional posterior growth that occurs from E10.0 to E13.5.

### Quantifying bipotency in the tailbud

To identify and characterize bipotent axial progenitors, we analyzed lineage relationships and descendant compositions of neural-restricted clades in E9.5 embryos. For each neural-restricted clade, sibling clades were defined as described above, and the distribution of non-neural developmental domains among sibling descendants was quantified. These distributions were examined as a function of the mean A-P position of the corresponding neural-restricted clade to assess how lineage outputs vary along the body axis. To visualize transcriptional relationships among these populations, we constructed a UMAP embedding (as described above) of cells belonging to neural-restricted clades and their sibling lineages. This analysis was restricted to posterior neural clades (mean A-P score >0.6), enabling visualization of the transcriptional continuum linking neural ectoderm, axial progenitors, and mesodermal derivatives.

To quantify bipotency over developmental time and space, lineage trees were partitioned into discrete timepoints between E7.0 and E9.0 using inferred branch lengths (0.2-day intervals). At each timepoint, clades were defined by cutting the tree at the corresponding depth. For each clade containing at least two neural ectoderm cells, we determined whether it also contained mesodermal descendants. Clades containing both neural and mesodermal cells were classified as bipotent. The fraction of bipotent clades was then calculated as a function of both clade depth and the mean A-P position of their neural descendants. Further, to assess the relationship between bipotent clades and transcriptionally defined axial progenitors, we focused on clades identified at E8.0 and calculated the fraction of bipotent clades containing at least one cell annotated as an axial progenitor.

To compare the spatial outputs of bipotent progenitors, we analyzed the A-P positions of neural and mesodermal descendants. Because paraxial mesoderm represents the primary mesodermal output, we focused on these cells and calculated A-P scores as described above. For mesodermal cells, A-P scores primarily reflect Hox gene expression domains rather than precise anatomical position, as these expression domains are modestly shifted relative to those in the neural tube^108^. For each neural and paraxial mesoderm cell, we determined the inferred time of the most recent common ancestor between these two populations. Cells were grouped by this divergence time (E7.0–E9.0, 0.2-day intervals), and the mean A-P positions of neural and mesodermal populations were calculated within each bin. This analysis enabled comparison of spatial positioning between lineage-related neural and mesodermal outputs over developmental time.

### Proliferation score

To estimate relative proliferation rates across cell populations, we quantified the number of close lineage relatives for each cell within reconstructed lineage trees. Close relatives were defined as cells sharing a common ancestor within the previous 24 hours based on branch length estimates. While this metric is noisy at the single-cell level due to imperfect branch length estimation and subsampling, averaging across thousands of cells representing each cell type yields robust estimates of relative proliferation. This metric reflects the net expansion rate of a lineage over the preceding 24 hours, integrating both cell division and cell loss. Thus, populations with higher proliferation scores have generated more surviving close relatives over that interval, whereas lower scores may reflect slower proliferation, greater cell loss, or both.

## Supplementary Material

Supplement 1

1

## Data Availability

Plasmids generated in this study will be made available on Addgene. All code and data will be made available at the time of publication.
