# Supplementary material for: Comprehensive Lineage Tracing Maps the Landscape of Cell Fate Decisions in Mouse Embryogenesis
Source: bioRxiv. 2026 May 9:2026.05.07.722278. Preprint. [Version 1] doi: 10.64898/2026.05.07.722278 (PMC13174701; doi:10.64898/2026.05.07.722278)
Supplement: Supplementary file 1 [file NIHPP2026.05.07.722278v1-supplement-2.pdf]

bioRxiv preprint doi: https://doi.org/10.64898/2026.05.07.722278; this version posted May 9, 2026. The copyright holder for this preprint (which was not certified by peer review) is the author/funder, who has granted bioRxiv a license to display the preprint in perpetuity. It is made available under aCC-BY 4.0 International license.

Supplementary Figures

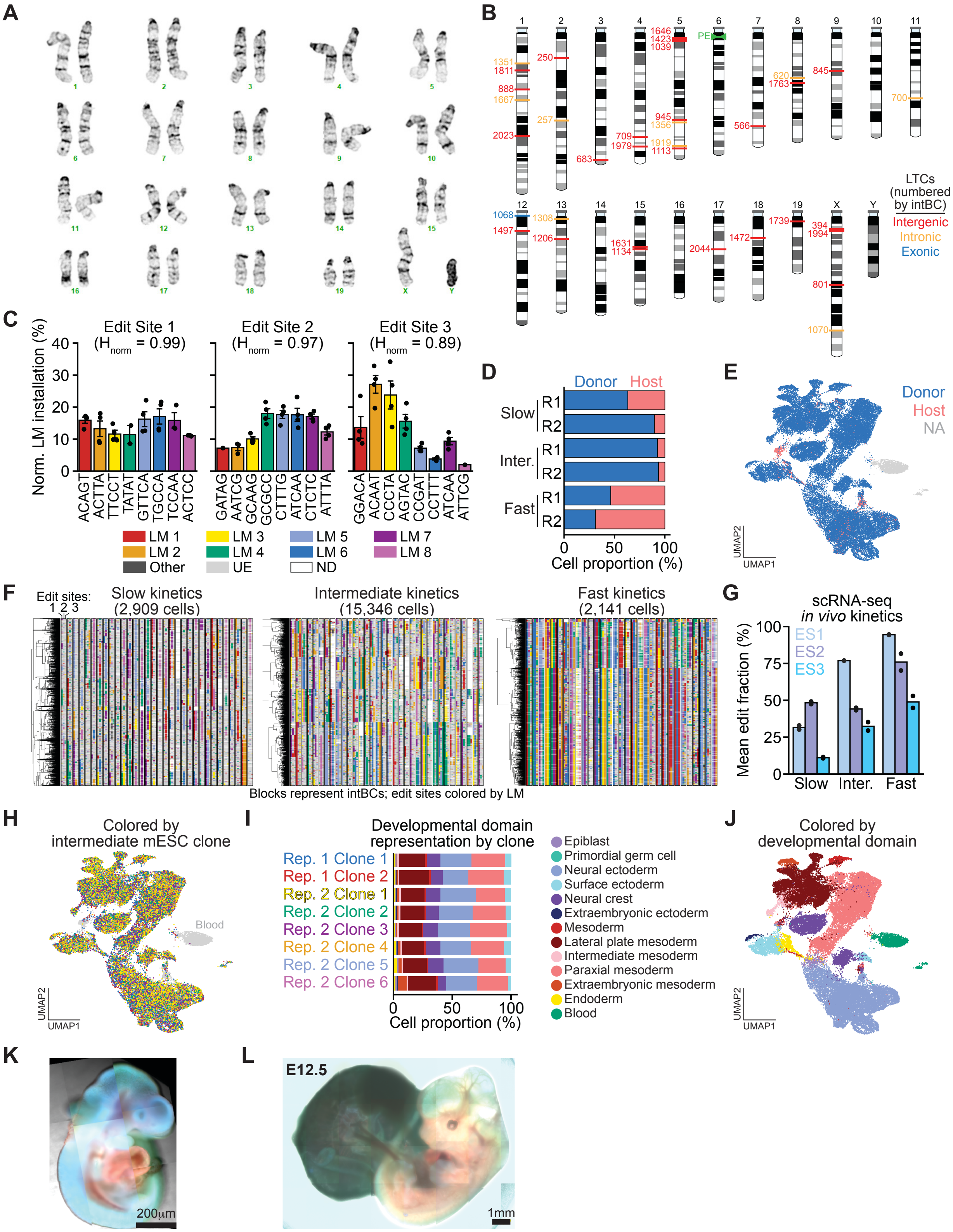

Fig. S1. Validation of PEtracer for mouse developmental studies. (**A**) Karyotype analysis of mouse embryonic stem cells harboring a homozygous Rosa26 knock-in of Cre-activateable

bioRxiv preprint doi: https://doi.org/10.64898/2026.05.07.722278; this version posted May 9, 2026. The copyright holder for this preprint (which was not certified by peer review) is the author/funder, who has granted bioRxiv a license to display the preprint in perpetuity. It is made available under aCC-BY 4.0 International license.

PEmax-T2A-GFP and 35 unique, genomically integrated lineage tracing cassettes (LTCs). (**B**) Genomic positions of integrated LTCs numbered by integration barcode (intBC) and colored by genomic context: intergenic (red), intronic (orange), a single exonic integration in *Ncoa1* (blue), and Rosa26 PEmax-T2A-GFP knock-in on chromosome 6 (green). (**C**) Normalized (Norm.) lineage mark (LM) installation efficiencies for all eight LMs at each of the three edit sites (ESs) in intermediate-kinetics E9.5 embryos from bulk sequencing. Bars represent the mean of four biological replicates ± standard error of the mean; normalized entropy (H_{norm}) values provided for each ES. LMs are colored and ordered by their position in the pegArray. (**D**) Proportion of tracing donor cells (blue) and non-tracing host cells (salmon) assessed by single-cell RNA sequencing (scRNA-seq) for two biological replicates of E9.5 embryos generated from slow-, intermediate- (inter.), and fast-kinetics mESC lines. (**E**) UMAP embedding of two intermediate-kinetics E9.5 embryos colored by donor and host cell identity with primitive erythrocytes excluded. (**F**) Cellular phylogenies and character matrices for representative sub-sampled slow-, intermediate-, and fast-kinetics E9.5 embryos. Character matrix blocks represent LTCs with distinct intBCs and are colored by LM identity at ESs 1-3; cell number is indicated for each tree. (**G**) *In vivo* LM installation rates for donor cells from the two E9.5 embryo replicates in (D). (**H**) UMAP embedding from (E) colored by contributing mESC clone. (**I**) Proportions of cell types in E9.5 embryos derived from the mESC clones in (H). (**J**) UMAP from (E) embedding colored by developmental domain. Representative embryos derived from injection of activated PEtracer cells into tetraploidized host morulae at (**K**) E9.5 and (**L**) E12.5.

bioRxiv preprint doi: https://doi.org/10.64898/2026.05.07.722278; this version posted May 9, 2026. The copyright holder for this preprint (which was not certified by peer review) is the author/funder, who has granted bioRxiv a license to display the preprint in perpetuity. It is made available under aCC-BY 4.0 International license.

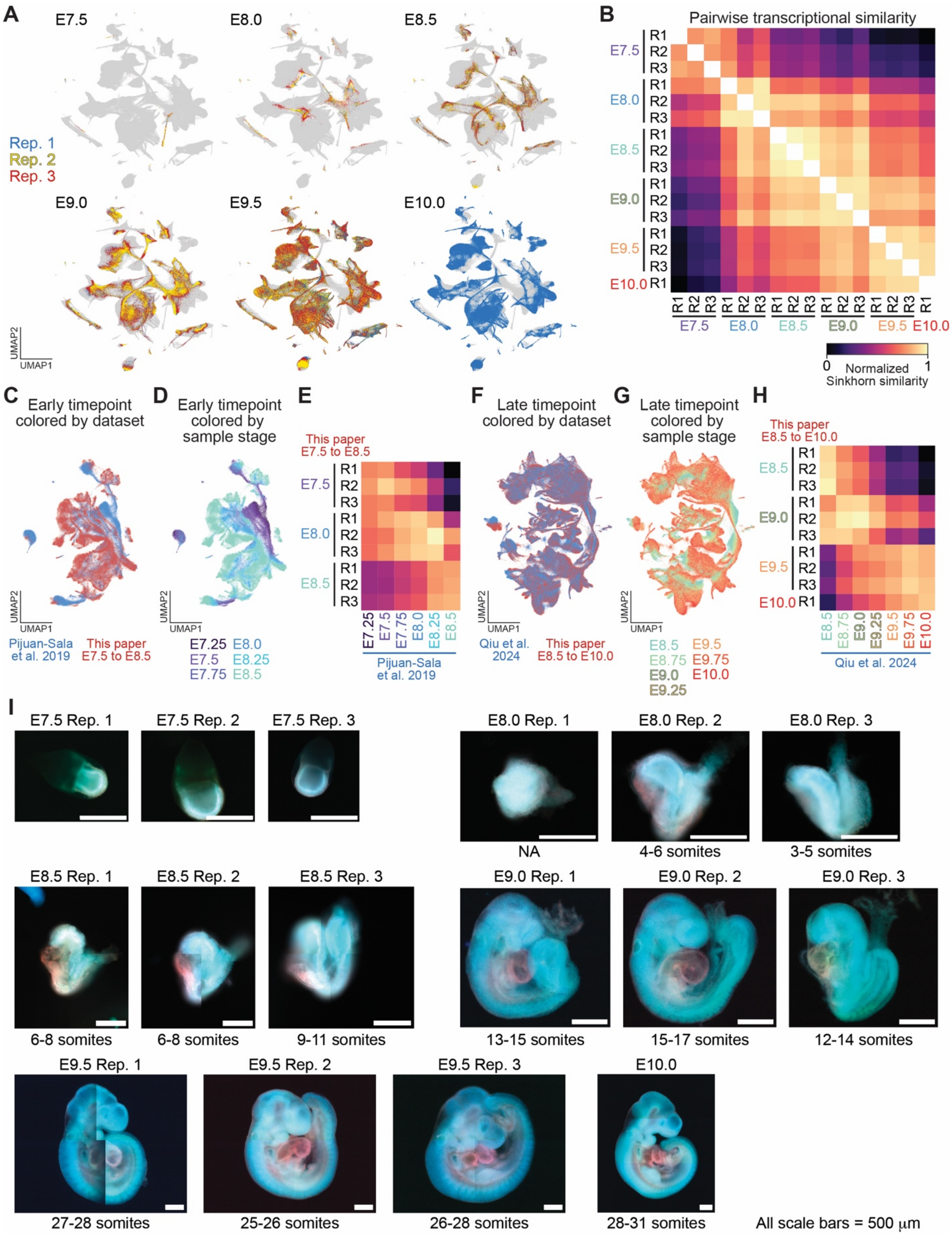

Fig. S2. Staging of sequenced PEtracer embryos. (**A**) UMAP embedding of all 1,792,634 cells from 16 embryos spanning E7.5 to E10.0 (see Fig. 2A). Cells from each of three biological

bioRxiv preprint doi: https://doi.org/10.64898/2026.05.07.722278; this version posted May 9, 2026. The copyright holder for this preprint (which was not certified by peer review) is the author/funder, who has granted bioRxiv a license to display the preprint in perpetuity. It is made available under aCC-BY 4.0 International license.

replicates for the indicated timepoint are colored separately (Rep. 1, blue; Rep. 2, yellow; Rep. 3, red), with cells from the other timepoints shown in gray. (**B**) Heatmap of pairwise transcriptional similarity between all replicates across timepoints, quantified by normalized Sinkhorn similarity. High within-stage similarity and graded between-stage similarity reflect the continuous progression of embryonic development. (**C-E**) Integration of the early timepoints from this study (E7.5-E8.5) with the reference dataset from Pijuan-Sala et al., 2019. (**C**) Joint UMAP colored by dataset of origin (Pijuan-Sala et al., blue; this paper, red). (**D**) The same UMAP colored by developmental timepoint. (**E**) Pairwise Sinkhorn similarity heatmap comparing replicates from this study (rows) to timepoints from Pijuan-Sala et al. (columns). (**F-H**) Analogous integration of the late timepoints from this study (E8.5-E10.0) with the reference dataset from Qiu et al., 2024. (**F**) Joint UMAP colored by dataset. (**G**) The same UMAP colored by developmental timepoint. (**H**) Pairwise Sinkhorn similarity heatmap comparing replicates from this study (rows) to timepoints from Qiu et al. (columns). (**I**) Images of embryos used in this study prior to dissociation and scRNA-seq. Colors represent an overlay of the three core PEtracer components (PEmax-T2A-GFP, mCherry:LTC, and BFP-linked pegArray). Somite counts are indicated below each embryo where determinable (NA, not assessable). E9.5 embryos are additionally shown in Fig. 1D. All scale bars = 500 μm.

bioRxiv preprint doi: https://doi.org/10.64898/2026.05.07.722278; this version posted May 9, 2026. The copyright holder for this preprint (which was not certified by peer review) is the author/funder, who has granted bioRxiv a license to display the preprint in perpetuity. It is made available under aCC-BY 4.0 International license.

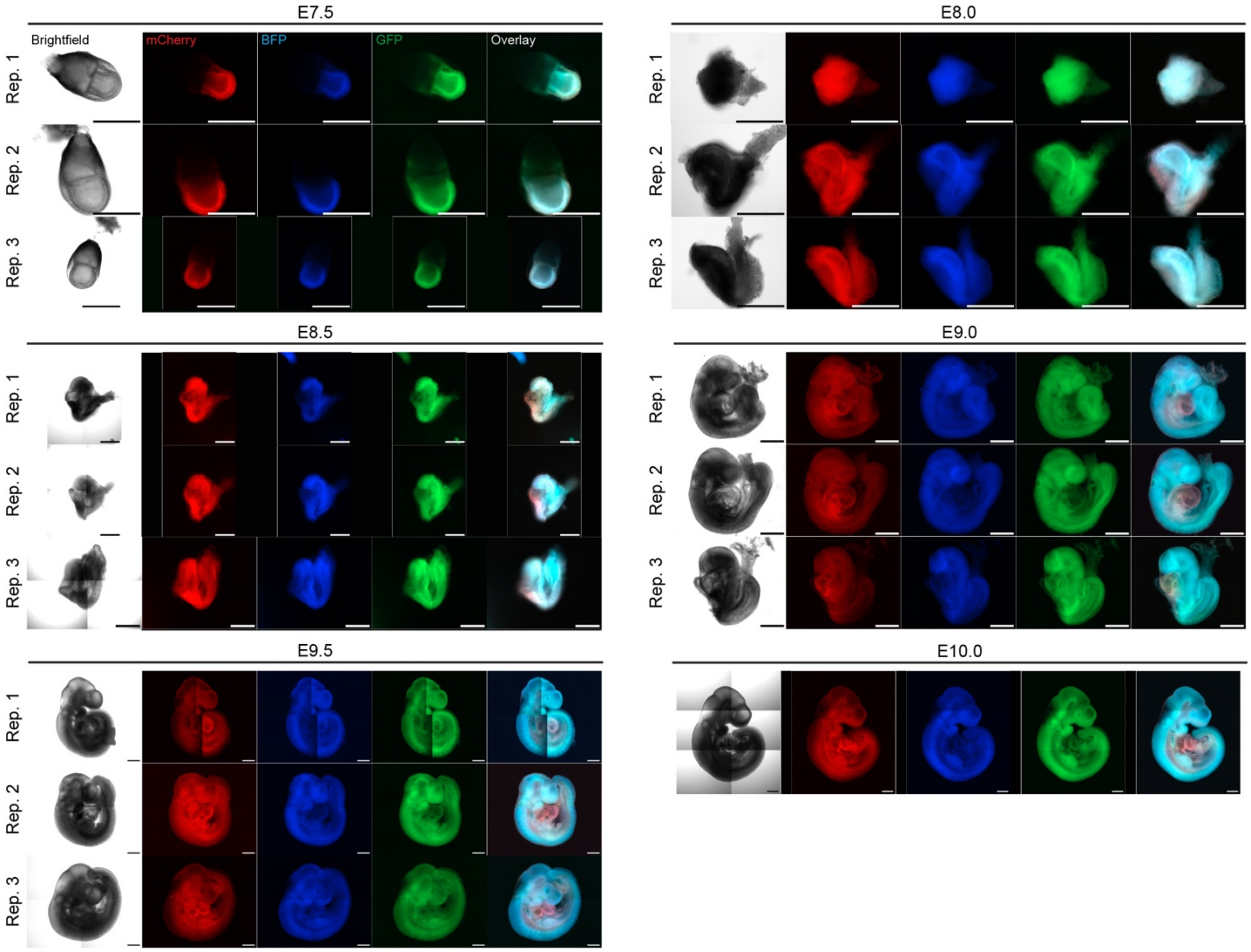

Fig. S3. Fluorescent characterization of PEtracer embryos collected across E7.5-E10.0. Representative imaging of PEtracer embryos spanning E7.5 to E10.0 prior to dissociation and sequencing. For each embryo, four channels are shown: brightfield, PEmax-T2A-GFP (green), mCherry-linked LTCs (red), and BFP-linked pegArray (blue), along with an overlay of all fluorescent signals. Embryos are displayed in chronological order of developmental stage to illustrate progressive changes in morphology and reporter expression across gastrulation and early organogenesis. Biological triplicates were collected for each developmental stage. All scale bars = 500 µm. Overlays match Fig. S2 and E9.5s are also shown in Fig. 1D.

bioRxiv preprint doi: https://doi.org/10.64898/2026.05.07.722278; this version posted May 9, 2026. The copyright holder for this preprint (which was not certified by peer review) is the author/funder, who has granted bioRxiv a license to display the preprint in perpetuity. It is made available under aCC-BY 4.0 International license.

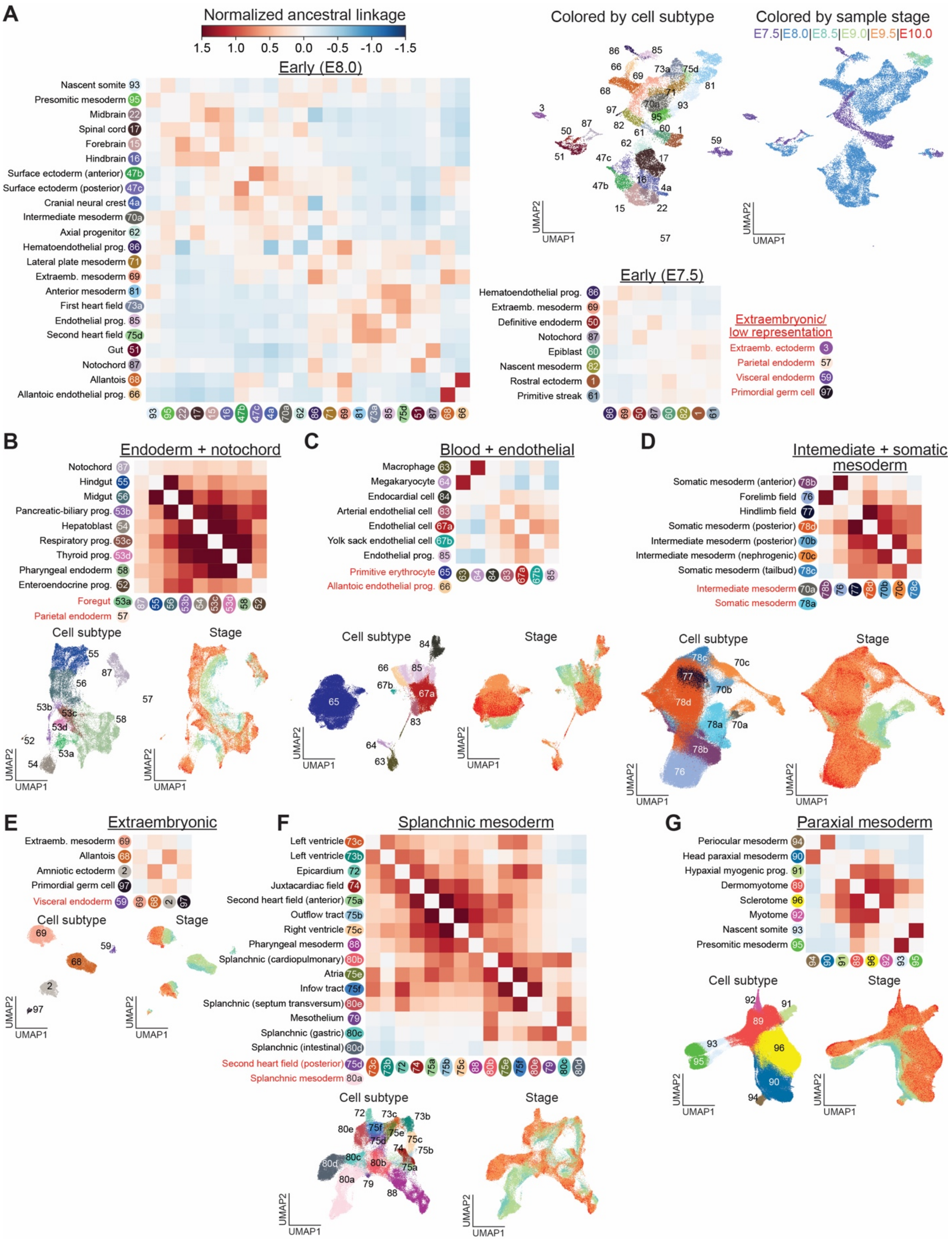

Fig. S4. Cell subtype ancestral linkage and embryonic staging. Ancestral linkage heatmaps with cell subtype labels and UMAP embeddings colored by cell subtype and sample stage for the

bioRxiv preprint doi: https://doi.org/10.64898/2026.05.07.722278; this version posted May 9, 2026. The copyright holder for this preprint (which was not certified by peer review) is the author/funder, who has granted bioRxiv a license to display the preprint in perpetuity. It is made available under aCC-BY 4.0 International license.

following developmental domains: (**A**) Early embryonic timepoints (E7.5 and E8.0), (**B**) endoderm and notochord, (**C**) blood and endothelial, (**D**) intermediate and somatic mesoderm, (**E**) extraembryonic, (**F**) splanchnic mesoderm, and (**G**) paraxial mesoderm. Cell subtypes included in UMAPs but excluded from the ancestral linkage heatmaps due to low representation are colored in red.

bioRxiv preprint doi: https://doi.org/10.64898/2026.05.07.722278; this version posted May 9, 2026. The copyright holder for this preprint (which was not certified by peer review) is the author/funder, who has granted bioRxiv a license to display the preprint in perpetuity. It is made available under aCC-BY 4.0 International license.

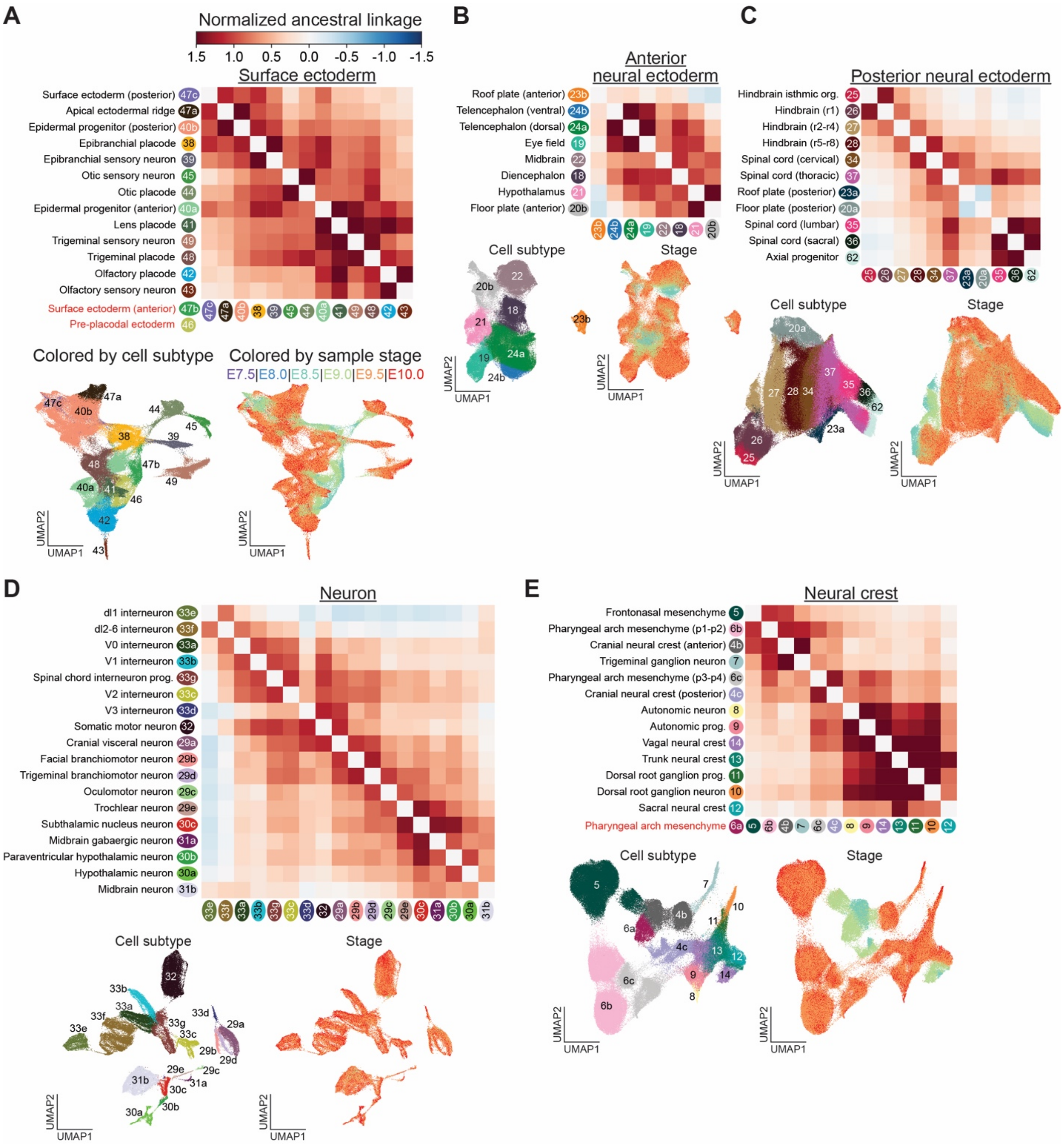

Fig. S5. Cell subtype ancestral linkage and embryonic staging. Ancestral linkage heatmaps with cell subtype labels and UMAP embeddings colored by cell subtype and sample stage for the following developmental domains: (**A**) surface ectoderm, (**B**) anterior neural ectoderm, (**C**) posterior neural ectoderm, (**D**) neurons, and (**E**) neural crest. Cell subtypes included in UMAPs but excluded from the ancestral linkage heatmaps due to low representation are colored in red.

bioRxiv preprint doi: https://doi.org/10.64898/2026.05.07.722278; this version posted May 9, 2026. The copyright holder for this preprint (which was not certified by peer review) is the author/funder, who has granted bioRxiv a license to display the preprint in perpetuity. It is made available under aCC-BY 4.0 International license.

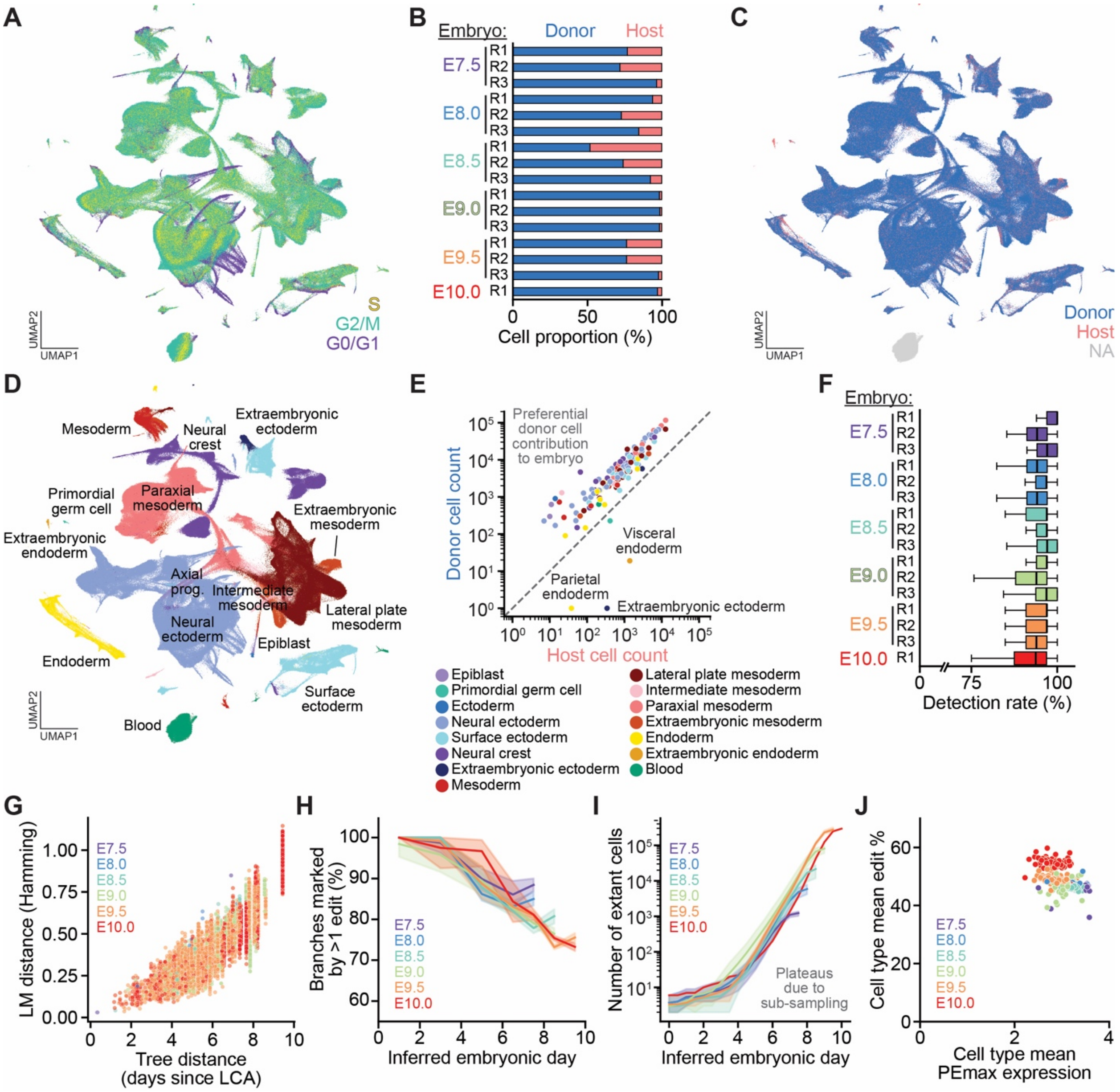

Fig. S6. Validation of PEtracer chimeric embryos. (**A**) UMAP embedding of all 1,792,634 cells from 16 embryos spanning E7.5 to E10.0 (see Fig. 2A), colored by cell cycle phase. Purple striations represent committed neuron populations that have exited the cell cycle. (**B**) Quantification of (B) showing proportion of tracing donor and non-tracing host cells across all 16 embryos (mean 85%, range 52-99%). (**C**) UMAP colored by donor (blue) versus host (salmon) cell identity, excluding primitive erythrocytes. (**D**) UMAP colored by developmental domain. (**E**) Relative contributions of donor and host cells to each developmental domain across all embryos. (**F**) Lineage mark (LM) detection rate by scRNA-seq across all sampled embryos. (**G**) Pairwise LM distance versus phylogenetic distance across all reconstructed trees, colored by embryonic timepoint. (**H**) Mean fraction of branches marked by at least one edit across all sampled embryos, colored by timepoint. (**I**) Mean estimated number of extant cells per embryonic timepoint as a function of inferred time based on branch length estimates. (**J**) PEmax expression versus mean edit fraction of cell types colored by embryonic timepoint.

bioRxiv preprint doi: https://doi.org/10.64898/2026.05.07.722278; this version posted May 9, 2026. The copyright holder for this preprint (which was not certified by peer review) is the author/funder, who has granted bioRxiv a license to display the preprint in perpetuity. It is made available under aCC-BY 4.0 International license.

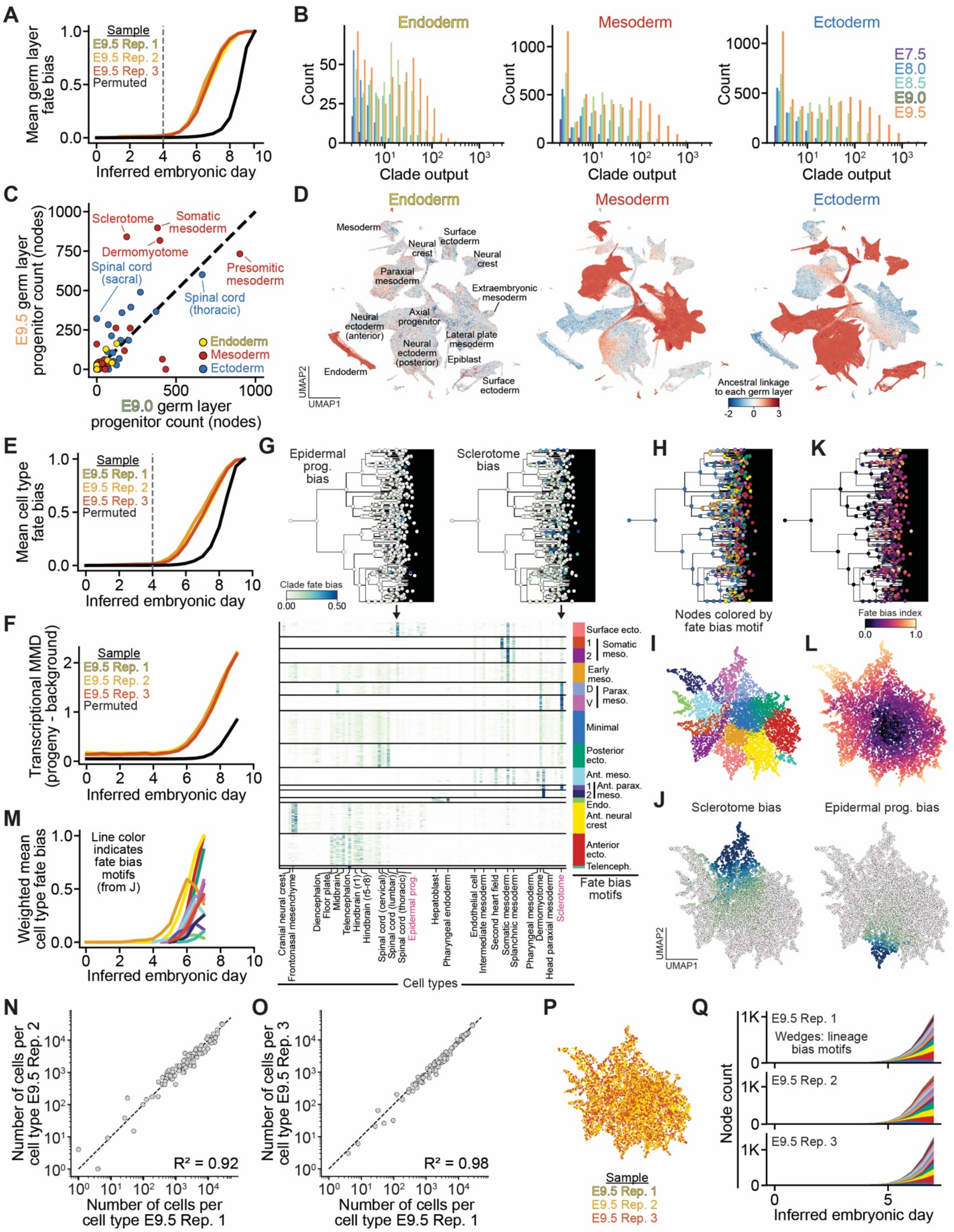

Fig. S7. Quantification of fate bias, progenitor contributions, and reproducibility across germ layers and cell types. (**A**) Mean germ layer-level fate bias over inferred embryonic day for

bioRxiv preprint doi: https://doi.org/10.64898/2026.05.07.722278; this version posted May 9, 2026. The copyright holder for this preprint (which was not certified by peer review) is the author/funder, who has granted bioRxiv a license to display the preprint in perpetuity. It is made available under aCC-BY 4.0 International license.

three E9.5 replicates and a permuted control (black). The dashed line marks the earliest time at which fate bias is statistically significant (permutation test) across all three replicates. (**B**) Distribution of clade outputs (number of descendant cells per fate-restricted node) for endoderm, mesoderm, and ectoderm at each triplicate sampled timepoint (E7.5-E9.5). (**C**) Number of germ layer-restricted clades contributing to each cell type at E9.0 vs. E9.5. Cell types are colored by germ layer assignment. (**D**) UMAP of all sampled cells colored by ancestral linkage to each germ layer (endoderm, mesoderm, ectoderm). Red indicates linkages that are closer than would be expected by chance and blue indicates the opposite. (**E**) Equivalent to (A), except showing mean cell type-level fate bias. (**F**) Mean transcriptional MMD (maximum mean discrepancy) between descendants of internal nodes and the overall distribution, for three E9.5 replicates and a permuted control (black). (**G**) Top: Example lineage tree (E9.5-R1-C2) with early ancestral nodes (inferred day <7 and >50 descendants) from E9.5 replicates colored by fate bias toward epidermal progenitor (left) and sclerotome (right) identities. Bottom: Heatmap representing early ancestral node fate bias (rows) across cell types (columns), grouped by dominant fate bias motif (colored labels, right). (**H**) Example lineage tree with ancestral nodes colored by assigned fate bias motif. (**I**) UMAP embedding of ancestral node fate distributions colored by assigned fate bias motif. (**J**) UMAP embedding from (I) colored by fate bias towards epidermal progenitor (left) and sclerotome (right) identities as in (G). (**K,L**) Example tree and UMAP from (H,I) colored by cell type-level fate bias index. (**M**) Mean cell type-level fate bias for each lineage motif weighted by abundance, with lines colored by fate bias motif (from J). (**N, O**) Cell type abundances between E9.5 replicate 1 and replicate 2 (N, R^2 = 0.92) or replicate 3 (O, R^2 = 0.98). Each point represents a cell type. (P) UMAP embedding of ancestral nodes from (I) colored by E9.5 replicate. (Q) Number of extant ancestral nodes over inferred embryonic day for each E9.5 replicate colored by fate bias motifs (from J).

bioRxiv preprint doi: https://doi.org/10.64898/2026.05.07.722278; this version posted May 9, 2026. The copyright holder for this preprint (which was not certified by peer review) is the author/funder, who has granted bioRxiv a license to display the preprint in perpetuity. It is made available under aCC-BY 4.0 International license.

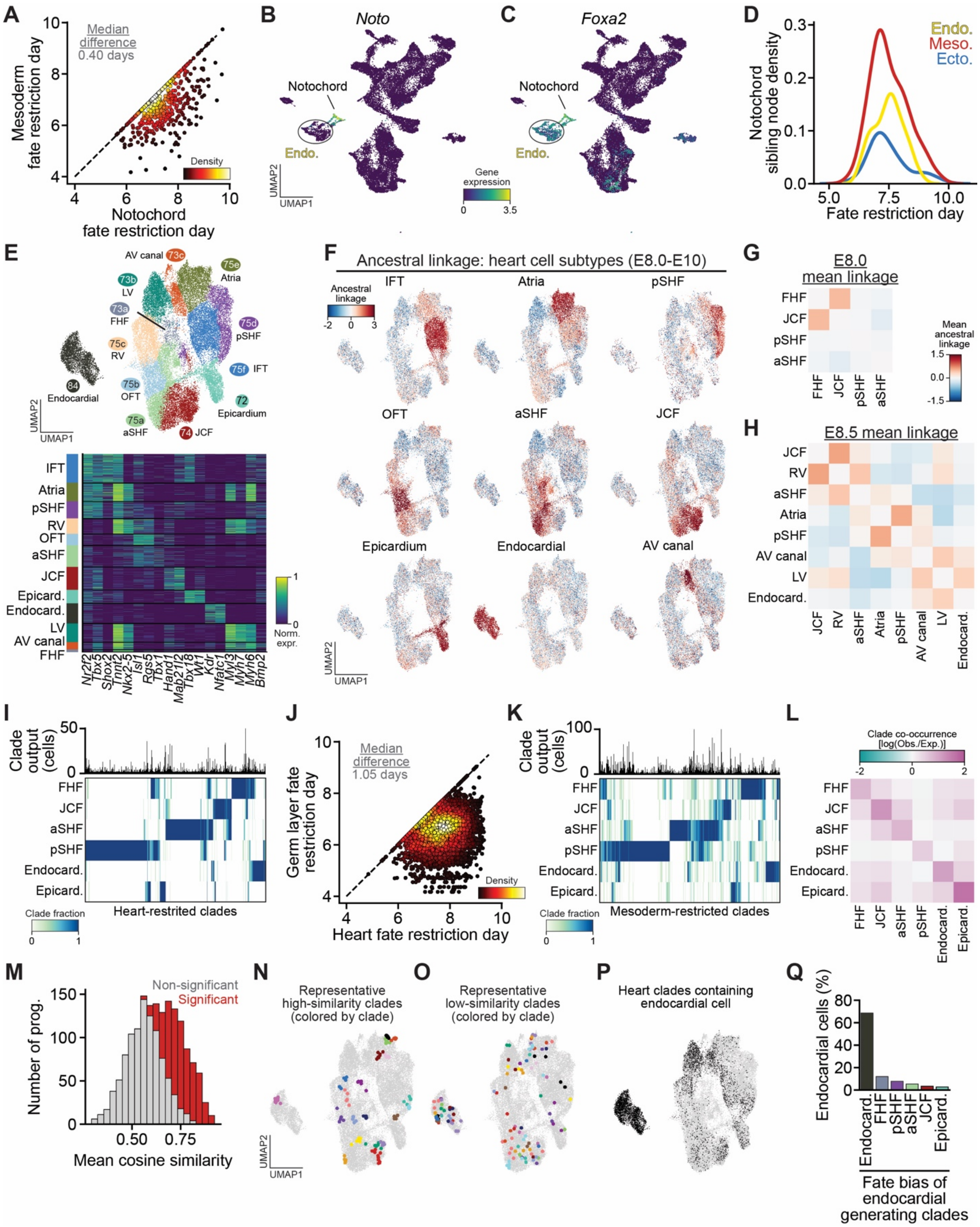

Fig. S8. Supporting analyses for notochord and heart field fate restriction dynamics. (**A-D**) Notochord: (**A**) Scatter plot of mesoderm fate restriction day versus notochord fate restriction day for each notochord-restricted clade. Points are colored by density and points on the diagonal indicate concurrent restriction; the median difference between mesoderm and notochord

bioRxiv preprint doi: https://doi.org/10.64898/2026.05.07.722278; this version posted May 9, 2026. The copyright holder for this preprint (which was not certified by peer review) is the author/funder, who has granted bioRxiv a license to display the preprint in perpetuity. It is made available under aCC-BY 4.0 International license.

restriction is 0.40 days. (**B**) UMAP of E7.5 and E8.0 cells colored by *Noto* expression, and (**C**) *Foxa2* expression. Notochord and endoderm populations are indicated. (**D**) Distribution of notochord fate restriction timing colored by germ layer bias of sibling nodes. (**E-Q**) Heart fields: (**E**) Top, UMAP of 12 annotated cardiac cell subtypes (as in Fig. 4F). Bottom, heatmap of normalized expression of marker genes used for cardiac cell type annotation. IFT = inflow tract, pSHF = posterior second heart field, RV = right ventricle, OFT = outflow tract, aSHF = anterior SHF, JCF = juxtacardiac field, Epicard. = epicardial cells, Endocard. = endocardium, LV = left ventricle, AV canal = atrioventricular canal, FHF = first heart field. (**F)** Cardiac UMAPs colored by ancestral linkage to each cardiac cell type across E8.0-E10.0 embryos. (**G**) Mean pairwise ancestral linkage between cardiac cell types at E8.0 and (**H**) E8.5. (**I**) Clustered heatmap of heart field (rows) representation for individual heart-restricted clades (columns). Clade output (number of cells) shown above. (**J**) Scatter plot of germ layer fate restriction day versus cardiac cell type fate restriction day for each heart-restricted clade. Points are colored by density and points on the diagonal indicate concurrent restriction; the median difference between mesoderm and heart restriction is 1.05 days. (**K**) Clustered heatmap of heart field (rows) representation for individual mesoderm-restricted clades (columns). Clade output (number of cells) shown above. (**L**) Heart field co-occurrence [log(observed/expected)] within mesoderm-restricted clades across sampled timepoints (E8.0-E9.5). (**M**) Histogram of mean cosine similarity of scVI embedding of heart cells within heart-restricted clades across E8.0-E10.0 embryos. Clades with statistically significant within-clade similarity are highlighted in red (permutation test). (**N**) Representative high-similarity and (**O**) low-similarity heart-restricted clades on the UMAP from (E). 10 colors indicate 10 randomly sampled clades. (**P**) Heart-restricted clades containing at least one endocardial cell (black) on the UMAP. (**Q**) Distribution of fate bias assignments for endocardial cell-generating heart-restricted clades.

bioRxiv preprint doi: https://doi.org/10.64898/2026.05.07.722278; this version posted May 9, 2026. The copyright holder for this preprint (which was not certified by peer review) is the author/funder, who has granted bioRxiv a license to display the preprint in perpetuity. It is made available under aCC-BY 4.0 International license.

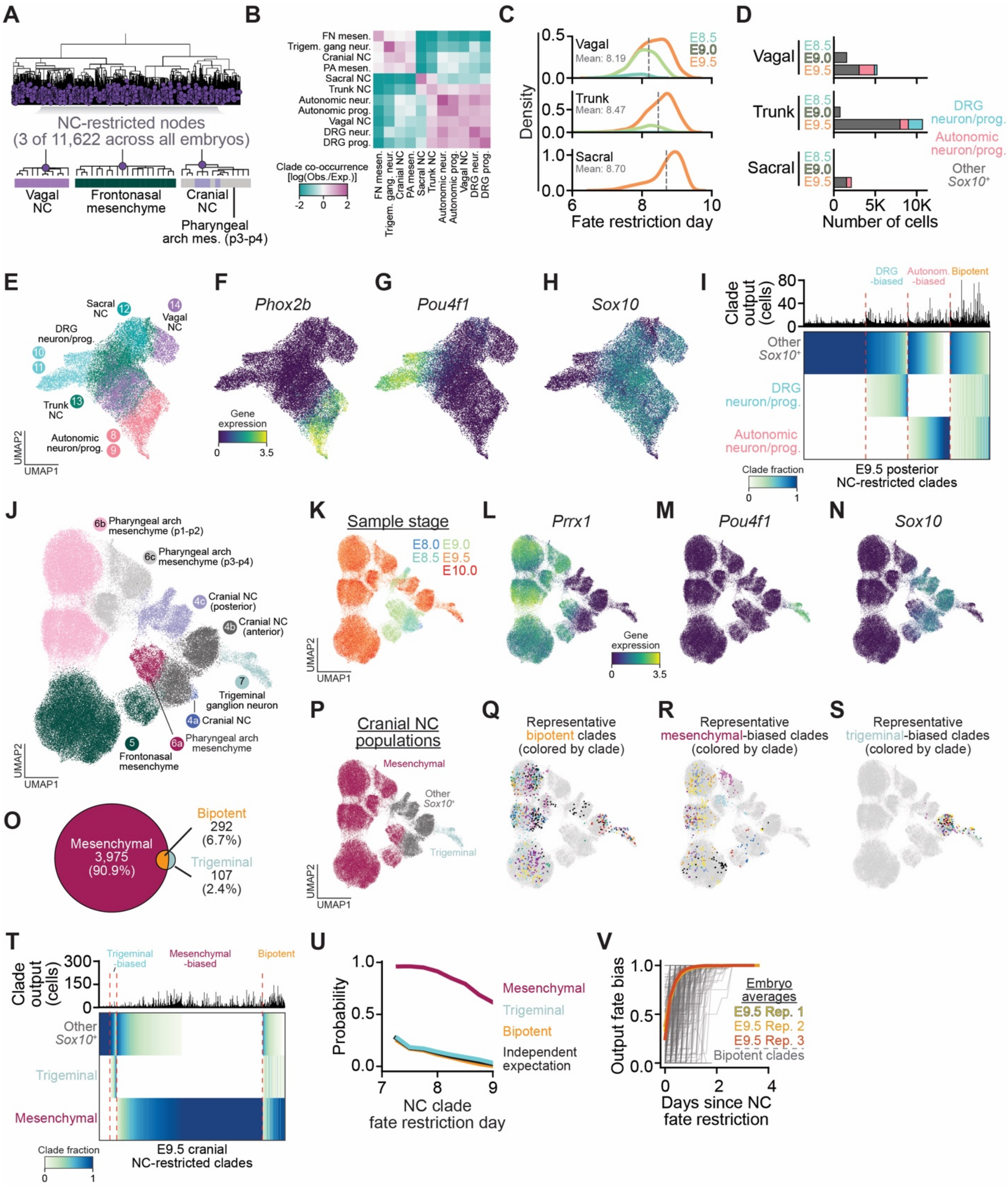

Fig. S9. Supporting analyses for neural crest (NC) fate restriction dynamics. (**A**) Example lineage tree (E9.5 replicate 2, clone 1; E9.5-R2-C1) with NC-restricted ancestral nodes marked (purple circles). Inset trees depict 3 of 11,622 fate-restricted nodes across all embryos. Color bars indicate cell type identity of descendant cells. mes. = mesenchyme. (**B**) Cell type co-occurrence [log(observed/expected)] within NC-restricted clades across embryos at E9.5 timepoint. FN = frontonasal, PA = pharyngeal arch, Trigem. Gang. neur. = trigeminal ganglion neuron, prog. =

bioRxiv preprint doi: https://doi.org/10.64898/2026.05.07.722278; this version posted May 9, 2026. The copyright holder for this preprint (which was not certified by peer review) is the author/funder, who has granted bioRxiv a license to display the preprint in perpetuity. It is made available under aCC-BY 4.0 International license.

progenitor, DRG = dorsal root ganglion. (**C-I**) Posterior NC: (**C**) Distribution of inferred vagal, trunk, and sacral NC fate restriction timing across sampled timepoints (E8.5-E9.5). Dashed line indicates the mean. (**D**) Cell type composition of vagal, trunk, and sacral NC clades across sampled timepoints, colored by DRG neuron/progenitor (teal), autonomic neuron/progenitor (pink), and other *Sox10*$^+$ cells (gray). (**E-H**) UMAP of posterior NC cells colored by cell type, reproduced from Fig. 4O colored by (**E**) cell type and expression of marker genes: (**F**) *Phox2b* for autonomic cells, (**G**) *Pou4f1* for sensory DRG cells, and (**H**) *Sox10* for NC progenitors. (**I**) Clustered heatmap of output type (rows) representation for individual E9.5 posterior NC-restricted clades (columns), grouped into DRG-biased, autonomic-biased, and bipotent categories. Clade output (number of cells) shown above. (**J-V**) Cranial NC: (**J**) UMAP of cranial NC cells and derivatives colored by (**J**) cell type, (**K**) sampled timepoint, and expression of marker genes: (**L**) *Prrx1* for mesenchymal cells, (**M**) *Pou4f1* for sensory trigeminal cells, and (**N**) *Sox10* for NC progenitors. (**O**) Venn diagram of cranial NC-restricted clades producing mesenchymal-biased, trigeminal-biased, or bipotent outputs at E9.5. (**P**) UMAP colored by cranial NC output: mesenchyme, trigeminal, and other *Sox10*$^+$ cells. (**Q**) Representative bipotent, (**R**) mesenchymal-biased, and (**S**) trigeminal-biased clades shown on the UMAP from (J). 10 colors indicate 10 randomly sampled clades. (**T**) Clustered heatmap of output type (rows) representation for individual E9.5 cranial NC-restricted clades (columns), grouped into trigeminal-biased, mesenchymal-biased, and bipotent categories. Clade output (number of cells) shown above. (**U**) Probability of an E9.5 clade containing mesenchymal, trigeminal, and bipotent outputs as a function of cranial NC-restricted clade depth (inferred embryonic day). Black line indicates the expected bipotent frequency under statistical independence between mesenchymal and trigeminal fate occurrence. (**V**) Mesenchymal versus trigeminal fate bias by time since NC fate restriction for individual bipotent clades (gray) and averaged across E9.5 replicates (colored).

bioRxiv preprint doi: https://doi.org/10.64898/2026.05.07.722278; this version posted May 9, 2026. The copyright holder for this preprint (which was not certified by peer review) is the author/funder, who has granted bioRxiv a license to display the preprint in perpetuity. It is made available under aCC-BY 4.0 International license.

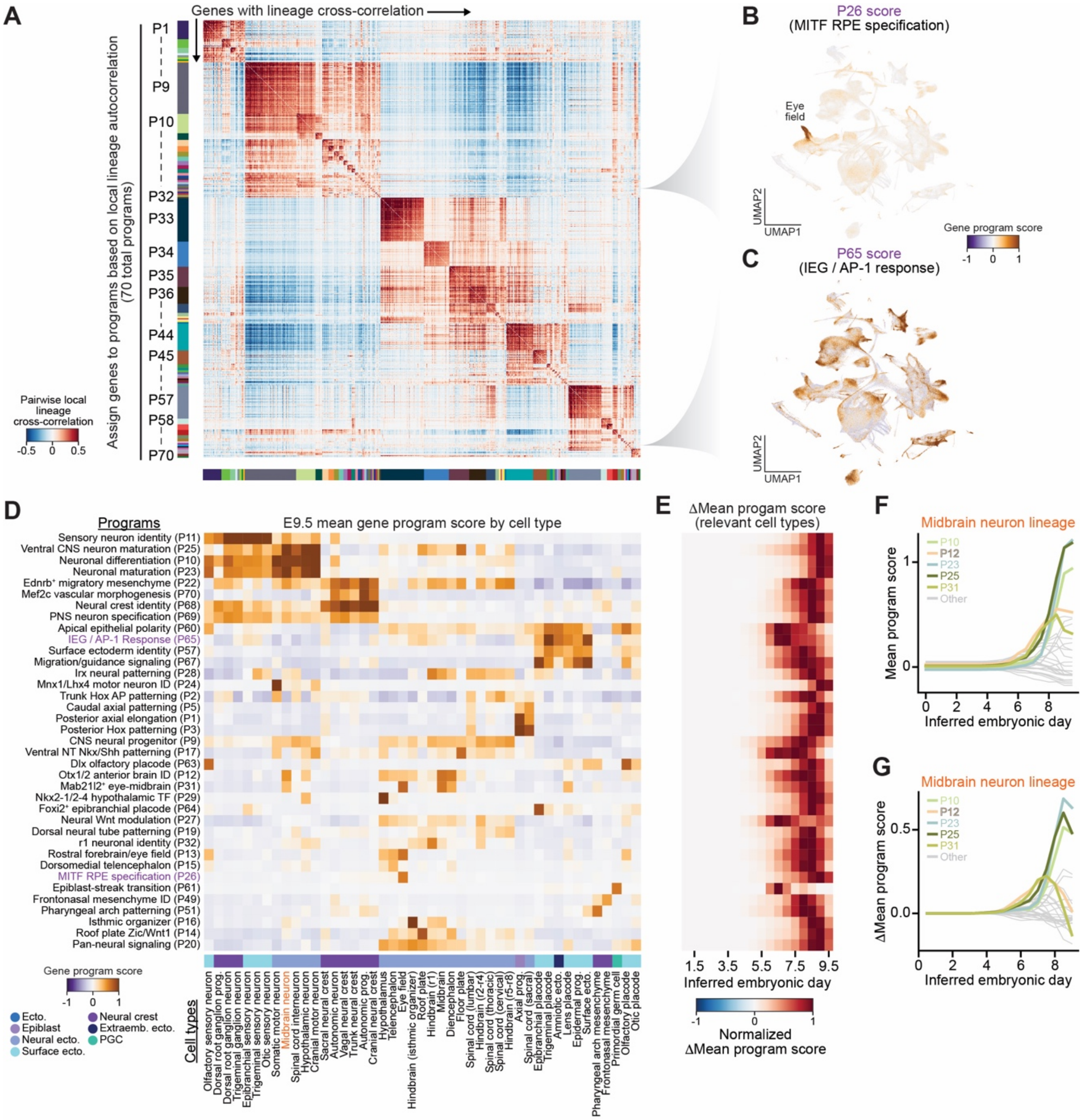

Fig. S10. Gene regulatory program nomination and activity across ectodermal cell types. (**A**) Pairwise local lineage cross-correlation matrix for 1,322 genes assigned to programs across all embryos. Genes are ordered by their hierarchical clustering assignment via Hotspot, revealing blocks of coordinated structure. Colored bars indicate assignment of genes to individual gene programs (P1-P70). (**B**) UMAP of all sampled cells colored by P26 (MITF RPE specification) or (**C**) P65 (IEG/AP-1 response) program scores. Cell type-specific activation of P26 in the eye field is annotated in (B). For a full list of gene regulatory program names, abbreviations, and descriptions of their assignment see **Table S3**. (**D**) Mean score for each program (rows) across ectodermal cell types (columns) in E9.5 embryos. Cell type developmental domain assignments are indicated by colored bars along the bottom. Highlighted programs in purple: P65 (IEG/AP-1 response) and P26 (MITF RPE specification) from (B,C). Highlighted cell type in orange is midbrain neuron. (**E**) Change in mean program score (ΔMean program score) over inferred embryonic day in E9.5 lineage trees for relevant cell types (mean >0.5 in at least one timepoint),

bioRxiv preprint doi: https://doi.org/10.64898/2026.05.07.722278; this version posted May 9, 2026. The copyright holder for this preprint (which was not certified by peer review) is the author/funder, who has granted bioRxiv a license to display the preprint in perpetuity. It is made available under aCC-BY 4.0 International license.

normalized per program. (**F**) Mean program score and (**G**) change in mean program score over inferred embryonic day for gene programs within the midbrain neuron lineage. Programs include P10 (neuronal differentiation), P12 (Otx1/2 anterior brain ID), P23 (neuronal maturation), P25 (ventral CNS neuron maturation), and P31 (Mab21l2^{+} eye-midbrain).

bioRxiv preprint doi: https://doi.org/10.64898/2026.05.07.722278; this version posted May 9, 2026. The copyright holder for this preprint (which was not certified by peer review) is the author/funder, who has granted bioRxiv a license to display the preprint in perpetuity. It is made available under aCC-BY 4.0 International license.

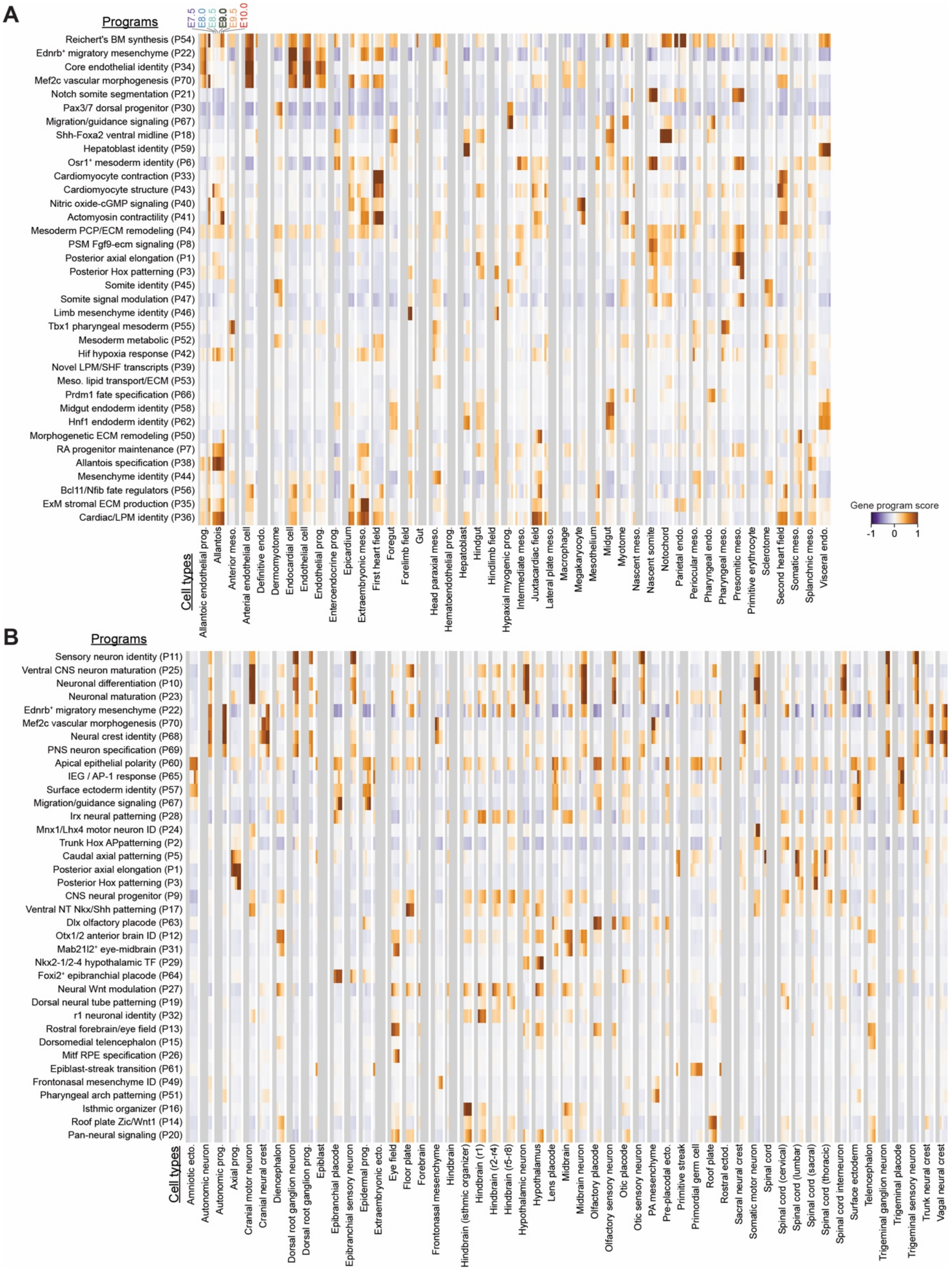

Fig. S11. Gene program activity across cell types over time. Gene program activity (rows) across sampled timepoints from E7.5 to E10.0 for (**A**) mesodermal and endodermal as well as (**B**) ectodermal cell types. Timepoints where cell types are not present are shown in grey.

bioRxiv preprint doi: https://doi.org/10.64898/2026.05.07.722278; this version posted May 9, 2026. The copyright holder for this preprint (which was not certified by peer review) is the author/funder, who has granted bioRxiv a license to display the preprint in perpetuity. It is made available under aCC-BY 4.0 International license.

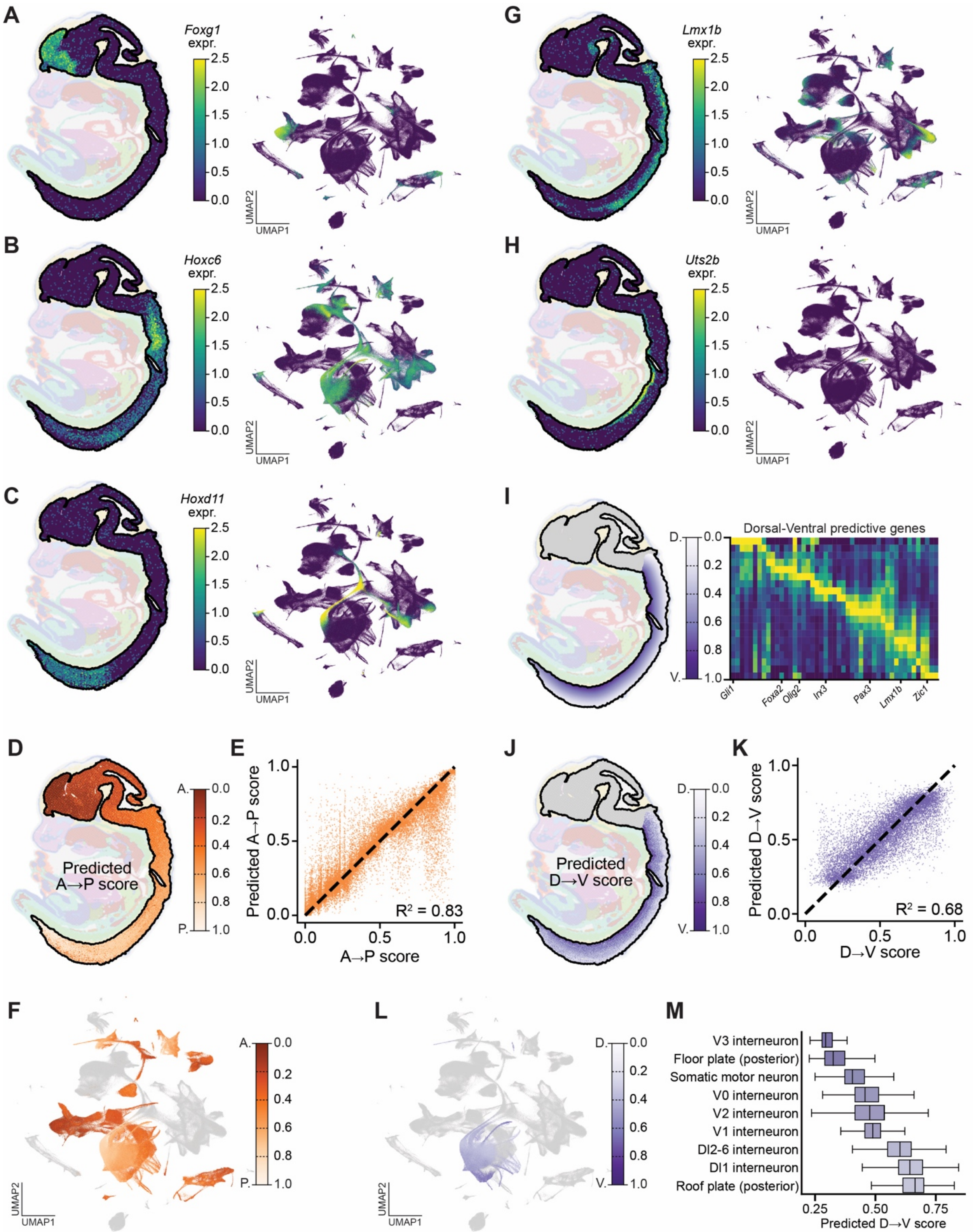

Fig. S12. Spatial transcriptomics-based inference of spatial axes. (**A-C**) Left: spatial transcriptomics data from E13.5 mouse embryo cross-section (Stereo-seq; Chen et al., 2022). Neural ectoderm is outlined and colored by expression of example anterior-posterior (A-P)

bioRxiv preprint doi: https://doi.org/10.64898/2026.05.07.722278; this version posted May 9, 2026. The copyright holder for this preprint (which was not certified by peer review) is the author/funder, who has granted bioRxiv a license to display the preprint in perpetuity. It is made available under aCC-BY 4.0 International license.

patterning genes, while other tissues are shown in the background for reference. Right: UMAP embedding of all cells in our dataset colored by the expression of the same genes: (**A**) *Foxg1*, (**B**) *Hoxc6*, and (**C**) *Hoxd11*. (**D**) Predicted A-P score of stereo-seq spots, using our model (5-fold cross validation). (**E**) Concordance of predicted and inferred A-P position across the stereo-seq dataset (R^2 = 0.83). (**F**) UMAP embedding of all cells in our dataset colored by predicted A-P score. (**G,H**) Same as (A-C) for dorsal-ventral (D-V) patterning genes: (**G**) *Lmx1b* and (**H**) *Uts2b*. (**I**) Left: Stereo-seq cross-section with spinal cord colored by D-V position. Right: Expression along the D-V axis of 54 patterning genes used to predict the D-V position of cells in our dataset. (**J,K**) Same as (D,E) D-V score. (**J**) Predicted D-V score of stereo-seq spots, using our model (5-fold cross validation) and (**K**) Concordance of predicted and inferred D-V position across the stereo-seq dataset (R^2 = 0.68). (**L**) UMAP embedding of all cells in our dataset colored by predicted D-V score. (**M**) Predicted D-V score distribution for spinal cord inter and motor neurons in addition to the roof and floor plates. Boxes indicate mean and interquartile ranges. Colors correspond to mean D-V score for each cell type.

bioRxiv preprint doi: https://doi.org/10.64898/2026.05.07.722278; this version posted May 9, 2026. The copyright holder for this preprint (which was not certified by peer review) is the author/funder, who has granted bioRxiv a license to display the preprint in perpetuity. It is made available under aCC-BY 4.0 International license.

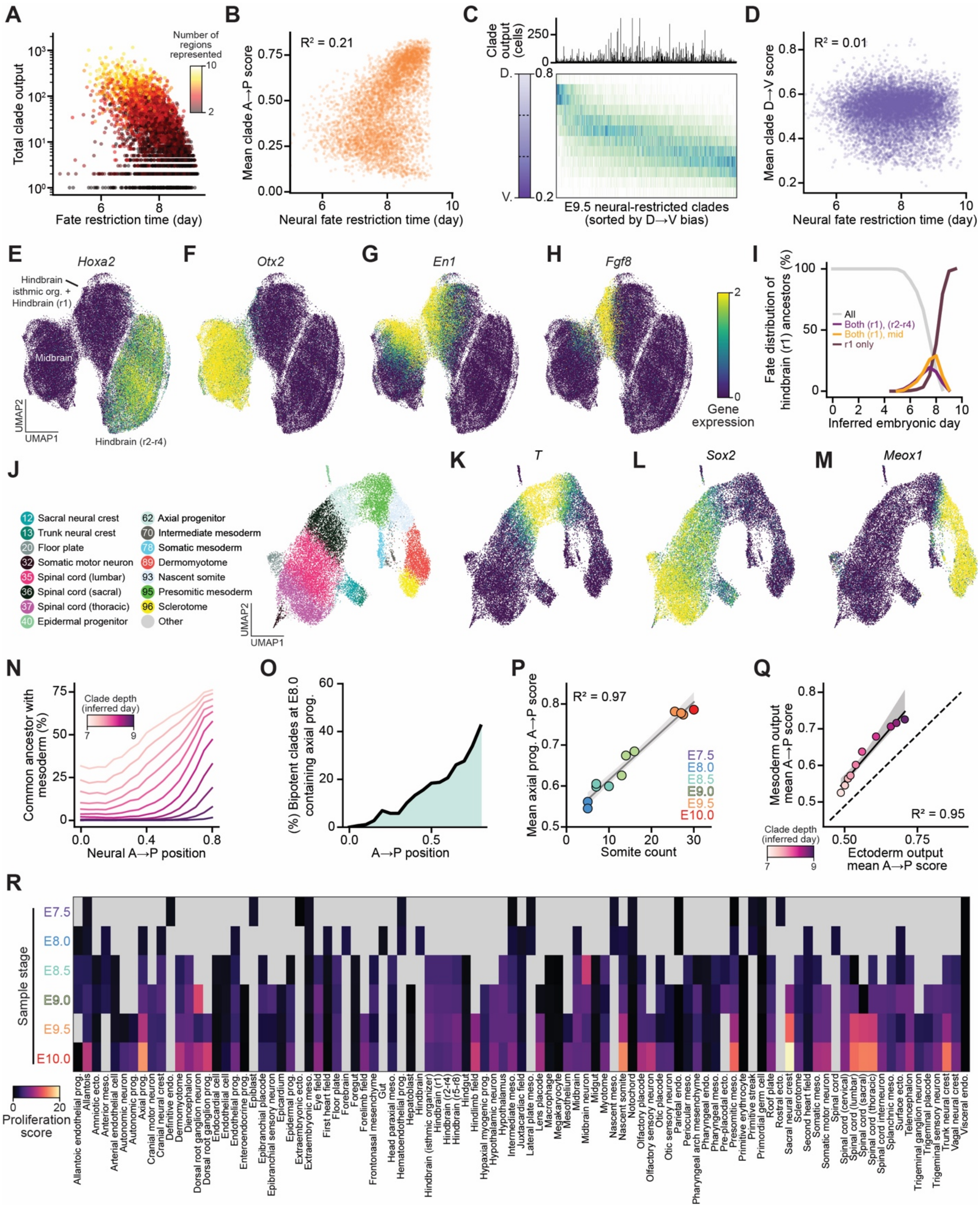

Fig. S13. Supporting analysis for spatial axes, regionalization, and axis elongation. (**A**) Total clade output (number of descendant cells) versus neural fate restriction time. Points are colored by the number of neural regions represented in each clade. (**B**) Mean A-P score of neural-restricted clades versus fate restriction time. (**C**) Heatmap showing the distribution of D-V

bioRxiv preprint doi: https://doi.org/10.64898/2026.05.07.722278; this version posted May 9, 2026. The copyright holder for this preprint (which was not certified by peer review) is the author/funder, who has granted bioRxiv a license to display the preprint in perpetuity. It is made available under aCC-BY 4.0 International license.

positions (rows) for spinal cord clades in E9.5 embryos with >10 descendants (columns), ordered by mean D-V score. Clade output (number of cells) shown above. (**D**) Mean D-V score of spinal cord clades versus fate restriction time. (**E-H**) UMAP of E9.5 midbrain, hindbrain (isthmic organizer), hindbrain (r1), and hindbrain (r2-r4) cells from Fig. 6N colored by expression of: (**E**) *Hoxa2*, (**F**) *Otx2*, (**G**) *En1*, and (**H**) *Fgf8*. (**I**) Percent of ancestors of hindbrain (r1) cells in E9.5 embryos that generate r1 only, both r1 and midbrain (mid.), both r1 and r2-r4, or all three, plotted by depth in the lineage tree. (**J-M**) UMAP of E9.5 posterior neural-restricted clades and their siblings colored by (**J**) cell type (numbering as in Fig. 2), (**K**) *T* expression, (**L**) *Sox2* expression, and (**M**) *Meox1* expression. (**N**) Fraction of neural cells by A-P position sharing a common ancestor with the mesoderm at clade depths from E7.0-E9.0 (inferred embryonic day). (**O**) Fraction of bipotent clades with common ancestor at E8.0 that contain observed axial progenitors at E9.5, plotted across the A-P axis. (**P**) Mean axial progenitor A-P score versus somite number across developmental timepoints. Trend line indicates linear regression fit (R^2 = 0.97). (**Q**) Mean A-P scores of mesodermal versus ectodermal outputs from bipotent clades at depths from E7.0-E9.0 (inferred embryonic day). Trend line indicates linear regression fit (R^2 = 0.93); dashed line indicates y = x. (**R**) Proliferation scores (calculated as mean number of cells sharing a common ancestor within the previous 24 hours) across cell types and developmental timepoints (E7.5-E10.0).
